# Cost‐effectiveness of broadly neutralizing antibody prophylaxis for HIV‐exposed infants in sub‐Saharan African settings

**DOI:** 10.1002/jia2.26052

**Published:** 2023-01-05

**Authors:** Caitlin M. Dugdale, Ogochukwu Ufio, Christopher Alba, Sallie R. Permar, Lynda Stranix‐Chibanda, Coleen K. Cunningham, Genevieve G. Fouda, Landon Myer, Milton C. Weinstein, Valériane Leroy, Elizabeth J. McFarland, Kenneth A. Freedberg, Andrea L. Ciaranello

**Affiliations:** ^1^ Medical Practice Evaluation Center Department of Medicine Massachusetts General Hospital Boston Massachusetts USA; ^2^ Division of Infectious Diseases Department of Medicine Massachusetts General Hospital Boston Massachusetts USA; ^3^ Harvard Medical School Boston Massachusetts USA; ^4^ Department of Pediatrics Weill Cornell Medicine New York New York USA; ^5^ Department of Pediatrics New York‐Presbyterian/Weill Cornell Medical Center New York New York USA; ^6^ Child and Adolescent Health Unit Faculty of Medicine and Health Sciences University of Zimbabwe Harare Zimbabwe; ^7^ Department of Pediatrics University of California Irvine Irvine California USA; ^8^ Department of Pediatrics Children's Hospital of Orange County Orange California USA; ^9^ Department of Pediatrics Duke University Medical Center Durham North Carolina USA; ^10^ Duke Human Vaccine Institute Durham North Carolina USA; ^11^ Division of Epidemiology and Biostatistics School of Public Health & Family Medicine University of Cape Town Cape Town South Africa; ^12^ Department of Health Policy and Management Harvard T.H. Chan School of Public Health Boston Massachusetts USA; ^13^ CERPOP, Inserm Toulouse University Université Paul Sabatier Toulouse France; ^14^ Department of Pediatrics University of Colorado Anschutz Medical Campus and Children's Hospital Colorado Aurora Colorado USA; ^15^ Division of General Internal Medicine Massachusetts General Hospital Boston Massachusetts USA

**Keywords:** broadly neutralizing antibodies, cost‐effectiveness analysis, HIV prevention and control, HIV‐1, infant HIV prophylaxis, vertical HIV transmission

## Abstract

**Introduction:**

Infant HIV prophylaxis with broadly neutralizing anti‐HIV antibodies (bNAbs) could provide long‐acting protection against vertical transmission. We sought to estimate the potential clinical impact and cost‐effectiveness of hypothetical bNAb prophylaxis programmes for children known to be HIV exposed at birth in three sub‐Saharan African settings.

**Methods:**

We conducted a cost‐effectiveness analysis using the CEPAC‐Pediatric model, simulating cohorts of infants from birth through death in Côte d'Ivoire, South Africa and Zimbabwe. These settings were selected to reflect a broad range of HIV care cascade characteristics, antenatal HIV prevalence and budgetary constraints. We modelled strategies targeting bNAbs to only WHO‐designated “high‐risk” HIV‐exposed infants (*HR‐HIVE*) or to all HIV‐exposed infants (*HIVE*). We compared four prophylaxis approaches within each target population: standard of care oral antiretroviral prophylaxis (*SOC*), and *SOC* plus bNAbs at birth (*1‐dose*), at birth and 3 months (*2‐doses*), or every 3 months throughout breastfeeding (*Extended*). Base‐case model inputs included bNAb efficacy (60%/dose), effect duration (3 months/dose) and costs ($60/dose), based on published literature. Outcomes included paediatric HIV incidence and incremental cost‐effectiveness ratios (ICERs) calculated from discounted life expectancy and lifetime HIV‐related costs.

**Results:**

The model projects that bNAbs would reduce absolute infant HIV incidence by 0.3–2.2% (9.6–34.9% relative reduction), varying by country, prophylaxis approach and target population. In all three settings, *HR‐HIVE–1‐dose* would be cost‐saving compared to *SOC*. Using a 50% GDP per capita ICER threshold, *HIVE‐Extended* would be cost‐effective in all three settings with ICERs of $497/YLS in Côte d'Ivoire, $464/YLS in South Africa and $455/YLS in Zimbabwe. In all three settings, bNAb strategies would remain cost‐effective at costs up to $200/dose if efficacy is ≥30%. If the bNAb effect duration were reduced to 1 month, the cost‐effective strategy would become *HR‐HIVE–1‐dose* in Côte d'Ivoire and Zimbabwe and *HR‐HIVE–2‐doses* in South Africa. Findings regarding the cost‐effectiveness of bNAb implementation strategies remained robust in sensitivity analyses regarding breastfeeding duration, maternal engagement in postpartum care, early infant diagnosis uptake and antiretroviral treatment costs.

**Conclusions:**

At current efficacy and cost estimates, bNAb prophylaxis for HIV‐exposed children in sub‐Saharan African settings would be a cost‐effective intervention to reduce vertical HIV transmission.

## 1 INTRODUCTION

Although the scale‐up of universal antiretroviral therapy (ART) in pregnancy and lactation has reduced vertical HIV transmission globally by over 20% since 2015, an estimated 150,000 paediatric HIV infections still occur annually [1]. Substantial barriers to early HIV diagnosis and ART initiation in pregnancy, maternal ART adherence and maternal retention in care contribute to ongoing high rates of vertical HIV transmission [1]. In high‐incidence settings, acute HIV infection during pregnancy or breastfeeding also contributes to residual paediatric HIV infections [2]. Novel biomedical interventions to address these vulnerabilities in the maternal HIV care cascade are needed to eliminate vertical HIV transmission [2].

Infant prophylaxis with long‐acting, injectable broadly neutralizing anti‐HIV antibodies (bNAbs) could provide supplemental protection from vertical HIV transmission throughout breastfeeding [2]. BNAbs have demonstrated excellent efficacy as postnatal prophylaxis in non‐human primates, including efficacy against intrapartum transmission if given within 24–30 hours after birth [2, 3, 4, 5]. In the only human efficacy study published to date, one bNAb, VRC01, did not prevent overall HIV acquisition among at‐risk adults; however, it demonstrated 75% efficacy against the acquisition of VRC01‐sensitive virus [6]. Several bNAbs (e.g. VRC01LS and VRC07‐523LS) have demonstrated safety, tolerability and potentially protective levels for 3 months when given subcutaneously to HIV‐exposed uninfected infants soon after birth and during infancy in clinical trials [7, 8]. There are at least 10 bNAbs moving forward in clinical trials both as prophylaxis and as treatment, although the timeline for regulatory approval is uncertain [9]. Several of these bNAbs, alone and in combination, possess *in vitro* neutralizing activity with greater breadth and potency than VRC01, and are nearing readiness for clinical use [10, 11].

While infant bNAb prophylaxis offers a promising approach to reduce vertical transmission, by potentially offering long‐acting protection throughout breastfeeding, there is concern that its implementation in resource‐limited settings will be cost‐prohibitive. Estimated bNAb production costs are $2–20 per 100 mg dose for infants, approximately 15‐fold lower than adult costs due to lower required doses [8, 12]. However, current formulations require an expensive cold chain of refrigerated storage until the administration and remain at potentially therapeutic levels for only 1–3 months following each dose [7, 13]. Therefore, infant bNAb prophylaxis is likely to be costly, but whether it may offer good value in high‐burden settings remains unclear.

We aimed to evaluate the potential clinical impact, costs and cost‐effectiveness of adding bNAb prophylaxis to existing HIV prevention approaches for known HIV‐exposed infants at birth in three sub‐Saharan African countries and to identify the optimal implementation strategy for bNAbs given the unique HIV epidemic characteristics and cost constraints of each setting.

## 2 METHODS

### 2.1 Study design and model overview

We used the validated Cost‐Effectiveness of Preventing AIDS Complications–Pediatric (CEPAC‐P) microsimulation model to project the clinical and economic impacts of hypothetical national bNAb HIV prophylaxis programmes for children known to be HIV exposed at birth in Côte d'Ivoire, South Africa and Zimbabwe [14, 15, 16]. We selected these countries to reflect a broad range of HIV epidemic characteristics with varying HIV prevalence, ART coverage, breastfeeding duration, early infant diagnosis (EID) uptake, healthcare costs and cost constraints (Table A5). Simulated infants enter the model at birth and face an initial perinatal risk and monthly postnatal risks of acquiring HIV. These risks are based on maternal HIV status, ART use, HIV viral load and breastfeeding practices. Children who acquire HIV draw a CD4% from a user‐defined distribution; without effective treatment, CD4% declines, which increases monthly risks of opportunistic infections (OIs) and AIDS‐related death. All known HIV‐exposed infants encounter opportunities for EID at multiple time points consistent with country‐specific guidelines and can undergo HIV testing after the development of an OI. Modelled children with HIV start ART immediately upon diagnosis and linkage to care; successful treatment leads to CD4% gains and reduced OI and mortality risks (see online Appendix). We followed Consolidated Health Economic Evaluation Reporting Standards in line with best‐practice advisories for cost‐effectiveness analyses (Table A4) [17]. This study was approved by the Mass General Brigham Human Research Committee.

### 2.2 Modelling infant HIV prophylaxis

We added an infant prophylaxis module to the CEPAC‐P model (Figure A1). The impact of prophylaxis on intrapartum transmission is modelled as a multiplier on perinatal transmission risks. For breastfeeding children, prophylaxis eligibility is evaluated each month, based on age and maternal characteristics; eligible children experience a probability of access and adherence to prophylaxis. If a prophylaxis dose is received, an efficacy multiplier reduces postnatal transmission risks for a user‐specified number of months.

### 2.3 Modelled cohorts and strategies

**Figure 1:**
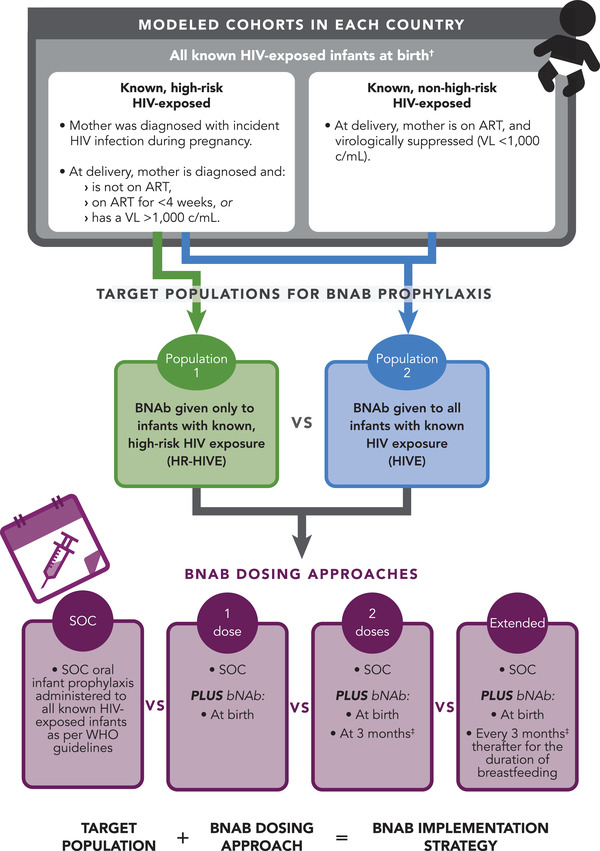
We modelled two separate cohorts of infants known to be HIV exposed at birth: high‐risk infants (by WHO criteria) and non‐high‐risk infants [18]. Outcomes from these two modelled cohorts were weighted by prevalence of high‐risk characteristics to generate overall clinical and economic outcomes for a population of all infants with known HIV exposure in each country setting evaluated (see online Appendix). Considering outcomes for the entire population of infants with known HIV exposure, we evaluated seven bNAb implementation strategies, which reflected assumptions regarding both the target population for bNAb administration (i.e. only high‐risk infants [HR‐HIVE] or all known HIV‐exposed infants [HIVE]) and the bNAb dosing approach (i.e. standard of care [SOC] oral infant prophylaxis without bNAbs, one dose of bNAbs at birth, two doses of bNAbs at birth and 3 months, and extended dosing of bNAbs throughout breastfeeding). In all strategies examined, all known HIV‐exposed infants were assumed to be eligible for SOC oral infant prophylaxis [18]. Abbreviations: ART, antiretroviral therapy; bNAb, broadly neutralizing antibody; SOC, standard of care; VL, viral load; WHO, World Health Organization. ^†^All modelled outcomes are presented for the entire population of all infants with known HIV exposure, reflecting a weighted average of outcomes between the high‐risk and non‐high‐risk modelled cohorts. ^‡^The bNAb effect duration was altered during sensitivity analysis and the frequency of administration was adjusted accordingly.

To reflect outcomes for all known HIV‐exposed children, we modelled two sub‐cohorts in each setting: known HIV‐exposed infants who are “high‐risk” at birth by World Health Organization (WHO) criteria (e.g. infants whose mothers experienced incident HIV infection during pregnancy, received less than 4 weeks of ART before delivery or had an HIV viral load >1000 c/ml near delivery) and known HIV‐exposed infants who are “non‐high‐risk” (Figure 1) [18]. We assumed all infants known to be HIV exposed at birth who are truly high‐risk have been identified as such and varied this assumption in sensitivity analyses. We also did not assume additional costs for identifying high‐risk infants. Outcomes for each sub‐cohort were aggregated and weighted based on sub‐cohort size to produce outcomes for the overall cohort of all children known to be HIV exposed at birth, including children who acquire HIV (irrespective of diagnosed infection) (see online Appendix). To examine the impact of hypothetical bNAb prophylaxis programmes focused on different target populations, we modelled strategies in which bNAbs are offered only to the sub‐cohort of high‐risk HIV‐exposed infants (*HR‐HIVE* strategies) and strategies in which bNAbs are offered to both modelled sub‐cohorts, comprising the entire population of all known HIV‐exposed infants (*HIVE* strategies; Figure 1). All known HIV‐exposed children are eligible for standard of care (SOC) oral antiretroviral prophylaxis, that is 6 weeks of nevirapine for non‐high‐risk infants and 12 weeks of dual nevirapine and zidovudine for high‐risk infants [18]. Within each target population, we investigated the impact of varied bNAb dosing approaches, including zero doses (*SOC*), one dose (at birth), two doses (at birth and 3 months) and extended bNAb dosing every 3 months throughout breastfeeding. Hybrid dosing approaches, in which high‐risk and non‐high‐risk infants are eligible to receive a different number of bNAb doses, were also explored (see online Appendix; Figure A2). In the model, we do not require HIV testing prior to bNAb administration; however, prophylaxis is stopped if the infant tests positive through routine HIV testing.

### 2.4 Outcomes

Modelled outcomes include cumulative HIV incidence and 5‐year survival, as well as discounted (3%/year) and undiscounted life expectancy and lifetime per‐person HIV‐related costs (in 2019 US dollars [USD]) from the healthcare system perspective.

We chose a lifetime time horizon to capture the full clinical and economic impacts of averting HIV infection among children. We also multiplied projected cumulative HIV incidence by the HIV‐exposed birth cohort size to estimate the number of HIV infections that would occur and would be averted by each strategy relative to *SOC*. To calculate incremental cost‐effectiveness ratios (ICERs) in $/year‐of‐life saved (YLS), we ordered strategies by ascending life expectancy and divided the difference in discounted costs by the difference in discounted life expectancy between non‐dominated strategies. Given controversies regarding cost‐effectiveness thresholds in resource‐limited settings, we identified the most effective strategy with an ICER below thresholds of both 20% and 50% of gross domestic product (GDP) per capita (see online Appendix; Table A3).

### 2.5 Model inputs

Country‐specific HIV prevalence and maternal and infant HIV care cascade characteristics were informed by UNAIDS estimates, Demographic and Health Surveys, Multiple Indicator Cluster Surveys and published literature (Table 1 and Table A5) [1, 6, 7, 8, 10, 12, 19, 20, 21, 22, 23, 24, 25, 26, 27, 28, 29, 30, 31, 32, 33, 34, 35, 36, 37, 38, 39, 40, 41, 42, 43, 44, 45, 46, 47, 48, 49, 50, 51, 52, 53, 54, 55, 56, 57, 58, 59, 60, 61, 62, 63, 64]. The three settings evaluated in this analysis were chosen to reflect a range of maternal awareness of chronic HIV infection (Côte d'Ivoire: 92%, South Africa: 99% and Zimbabwe: 98%) [1, 26, 35, 36, 53], ART uptake in pregnancy (Côte d'Ivoire: 87%, South Africa: 98% and Zimbabwe: 93%) [1], mean breastfeeding duration (Côte d'Ivoire: 14 months, South Africa: 6 months and Zimbabwe: 13 months) [35, 36, 51, 56] and 2019 GDP per capita (Côte d'Ivoire: $2276, South Africa: $6001 and Zimbabwe: $1464) [65]. The proportion of infants within each sub‐cohort was calculated using estimates of maternal HIV incidence and prevalence, ART coverage and virologic suppression from published data (Table 1). Data informing the natural history of HIV, ART efficacy, EID cascade and HIV‐related costs were also taken from published literature (Table A5) [14, 15].

**Table 1:** Selected base‐case model input parameters

Input parameter	Côte d'Ivoire	South Africa	Zimbabwe	Selected references
**Maternal characteristics**				
Probability of acute HIV infection in pregnancy, %	1.2	3.3	1.9	[19, 20, 21, 22, 23, 24]
Probability of known HIV status with acute/chronic infection in pregnancy, %	56/92	55/99	70/98	[1, 25, 26, 27, 28, 29, 30, 31, 32]
Received ART in pregnancy, %	87	98	93	[1]
HIV RNA ≤50/>1000 c/ml if on ART at delivery, %	58/23	66/14	69/9	[32, 33, 34]
Probability of delivering in a healthcare facility, %	70	96	76	[35, 36, 37]
Retention in HIV care at 6/12/24 months postpartum, %	87/87/79	88/87/79	88/87/79	[38]
HIV RNA <1000 c/ml among postpartum women on ART, by month postpartum	77–92	77–92	77–92	[39, 40, 41, 42, 43, 44, 45, 46, 47, 48]
**Vertical transmission rates**				
Perinatal transmission rate, one‐time %				
Chronic maternal HIV infection in pregnancy				
On ART, HIV RNA ≤50/50–1000/>1000 c/ml at delivery	0.24/1.45/4.14			[49]
Not on ART at delivery	19.70			[50]
Acute maternal HIV infection in pregnancy				
On ART at delivery	8.33			[50]
Not on ART at delivery	18.10			[50]
Postnatal transmission rate, %/month^a^				
On ART, HIV RNA ≤50/50–1000/>1000 c/ml	0.06/0.39/0.78			[49]
Not on ART	0.89			[50]
**Infant cohort characteristics**				
Estimated number of known HIV‐exposed infants born per year (thousands)	35	368	50	Derived from [1, 19, 20, 21, 22, 23, 24, 25, 26, 28, 29, 30, 31, 32, 33, 34, 35, 36, 51, 52, 53, 54]
Breastfed infants, % of known HIV‐exposed infants	97	66	94	[32, 35, 55]
Breastfeeding duration, mean (SD) month	14 (7)	6 (6)	13 (7)	[35, 42, 51, 56]
Infants meeting WHO high‐risk criteria at birth, %	44	19	24	Derived from [1, 19, 20, 21, 22, 23, 24, 25, 26, 28, 29, 30, 31, 32, 33, 34, 35, 36, 51, 52, 53]
**Infant HIV prophylaxis**				
Probability of receiving scheduled prophylaxis, %				
SOC oral infant prophylaxis (NVP +/– ZDV)^b^	86			[57]
bNAb prophylaxis (varies by age)^c^	31–71	54–96	62–89	Assum. based on [35, 36, 37]
Efficacy against intrapartum/postnatal transmission, %				
SOC oral infant prophylaxis (NVP +/– ZDV)^b,^ ^d^	75/71			[60, 66]
bNAb (in addition to efficacy of SOC)	60/60			Assum. based on [6, 10]
Duration of bNAb effect, month	3			Assum. based on [7, 8]
**Costs (2019 USD)**				
Infant oral prophylaxis (NVP +/– ZDV), per month	$7.19–$16.51			[61]
bNAb prophylaxis, per dose	$60.00			Assum. based on [12, 62, 63, 64]
HIV test by NAAT/antibody	$25.74/$4.01			[61]
Paediatric ART (range by age and weight, per month)				
First line (LPV/r or EFV‐based ART)	$10.29–$18.12			[61]
Second line (DTG‐based ART)	$7.86–$15.84			[61]
Adult ART (per month)				
First line (DTG‐based ART)	$5.30			[61]
Second line (PI‐based ART)	$23.15			[61]

Abbreviations: Assum., assumption; DTG, dolutegravir; EFV, efavirenz; LPV/r, ritonavir‐boosted lopinavir; NAAT, nucleic acid amplification test; PI, protease inhibitor; SD, standard deviation; SOC, standard of care.

^a^ All women included in this analysis are known to have HIV infection by delivery. Therefore, we did not model acute HIV infection during breastfeeding.

^b^ High‐risk infants received dual oral infant prophylaxis with nevirapine (NVP) + zidovudine (ZDV) for 12 weeks. Non‐high‐risk infants received NVP alone for 6 weeks.

^c^ The probability of receiving broadly neutralizing antibody (bNAb) prophylaxis was based on the probability of receiving World Health Organization (WHO) Expanded Programme on Immunization vaccines, by age at recommended immunization.

^d^ A reduction in the risk of intrapartum transmission with use of oral infant prophylaxis was only applied to infants born to mothers who were known to have HIV infection, but who were not on antiretroviral therapy (ART) at delivery. The impact of oral infant prophylaxis on intrapartum transmission among mothers on ART at delivery is already captured in the on ART perinatal transmission estimates.

We assumed that 86% of HIV‐exposed infants received SOC prophylaxis in all three countries [57], with 75% and 71% efficacy against intrapartum and postnatal transmission, respectively (Table 1, online Appendix; Table A1) [60, 66]. There are no published efficacy studies of bNAb prophylaxis among children. However, in the Antibody Mediated Protection (AMP) studies, HIV incidence with VRC01‐sensitive isolates was 75% lower among adults who received VRC01 than those who received a placebo [6]. Potent bNAbs alone or in combination have neutralizing activity for 84–92% of isolates in multiclade panels [10]. Therefore, we conservatively assumed an average efficacy of 60% for the hypothetical bNAb product against both intrapartum and postnatal transmission, by assuming that bNAb prophylaxis would lead to an approximate 75% reduction in transmission from the estimated 84% of bNAb‐susceptible circulating HIV strains. In the base case, we applied bNAb efficacy equally to high‐risk and non‐high‐risk infants in the *HIVE* strategies, varying it for each cohort separately in sensitivity analyses. In the base case, we assumed that the bNAb prophylaxis effect duration is 3 months, based on pharmacokinetic studies [7, 8]. Our assumed bNAb cost of $60/dose was driven primarily by estimated production costs (using the highest end of estimates to reflect potential bNAb combination products), but also includes cold‐chain, overhead and personnel costs, based on costs for vaccine delivery (see online Appendix; Table A2) [12].

### 2.6 Sensitivity analyses

We conducted univariate and multivariate sensitivity analyses to test the robustness of our findings to changes in key parameters. Given the uncertainty around bNAb uptake, efficacy, effect duration and costs, these inputs were varied widely in univariate sensitivity analyses. We also investigated the influence of uptake and efficacy of WHO‐recommended SOC prophylaxis; recognition of infants’ high‐risk status; breastfeeding duration; maternal retention in care and virologic suppression; vertical transmission risks; EID uptake; paediatric ART efficacy; and ART costs. In multivariate analyses, we simultaneously varied bNAb efficacy and costs to identify which strategies would be cost‐effective across a wide array of hypothetical bNAb characteristics at both the 20% and 50% GDP per capita thresholds.

## 3 RESULTS

### 3.1 Clinical outcomes

**Figure 2:**
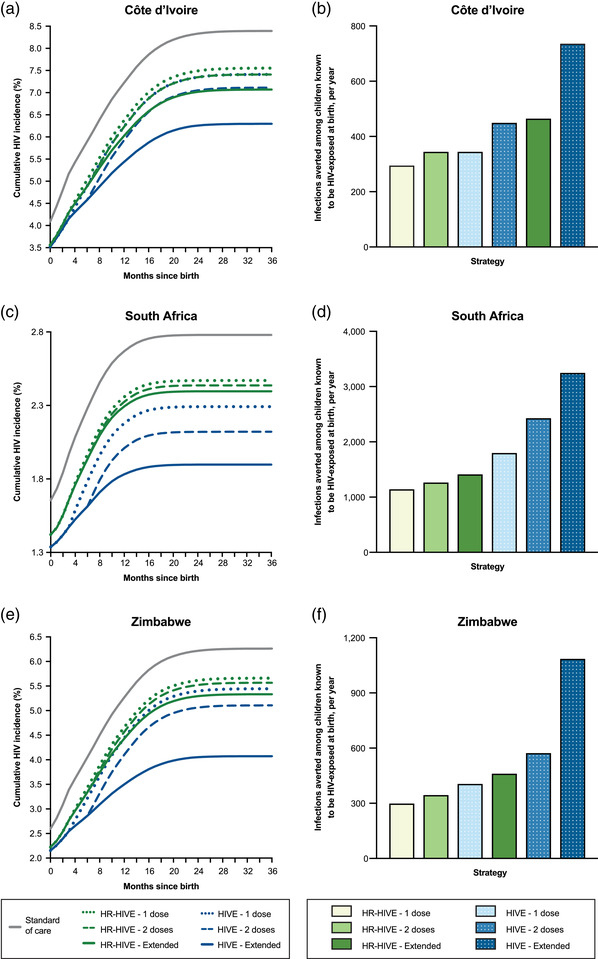
On the left‐sided panels, projected cumulative HIV incidence (Y‐axis) among all known HIV‐exposed children by months since birth (X‐axis) is shown for each bNAb implementation strategy using the base case in the three country settings examined: Côte d'Ivoire (Panel a), South Africa (Panel c) and Zimbabwe (Panel e). On the right‐sided panels, the number of annual HIV infections averted (Y‐axis) relative to standard of care is shown for each bNAb implementation strategy (X‐axis) in Côte d'Ivoire (Panel b), South Africa (Panel d) and Zimbabwe (Panel f). Abbreviations: bNAb, broadly neutralizing antibody; HIVE, all HIV‐exposed infants; HR‐HIVE, high‐risk HIV‐exposed infants.

In the *SOC* strategy, cumulative HIV incidence among all known HIV‐exposed infants was projected to be 8.4%, 2.8% and 6.3% in Côte d'Ivoire, South Africa and Zimbabwe, respectively (Table 2 and Figure 2, Panels a, c and e), similar to UNAIDS estimates (Table A6) [1]. Offering one dose of bNAbs at birth to only high‐risk HIV‐exposed infants (*HR‐HIVE–1‐dose*) would decrease projected cumulative HIV incidence to 7.6%, 2.5% and 5.7%. Additional doses throughout breastfeeding for high‐risk infants (*HR‐HIVE‐Extended*) would further reduce projected cumulative HIV incidence to 7.1%, 2.4% and 5.3%. In all three countries, extended bNAb dosing throughout breastfeeding for all known HIV‐exposed infants (*HIVE‐Extended*) would result in the lowest projected cumulative HIV incidence at 6.3%, 1.9% and 4.1%. Even in this most expansive bNAb strategy, the model projects that there would be considerable ongoing vertical transmission related to missed opportunities to prevent intrauterine transmission, incomplete bNAb uptake and efficacy, and suboptimal maternal ART uptake postpartum. However, when scaled to each country's population, bNAb strategies would still avert 300–740 projected paediatric HIV infections in Côte d'Ivoire, 1140–3250 infections in South Africa and 300–1090 infections in Zimbabwe annually compared with *SOC* (Table 2 and Figure 2, Panels b, d and f).

**Table 2:** Clinical and economic outcomes of bNAb implementation strategies for all known HIV‐exposed infants

	Cumulative HIV incidence (%)	Children with known exposure acquiring HIV (*n*/year)^a^	5‐year survival among children with known exposure (%)	Undiscounted life expectancy (years)	Discounted life expectancy (years)	Discounted costs ($)	ICER ($/YLS)
**Côte d'Ivoire (CET: ICER < $455/YLS [20% GDP per capita], ICER < $1138 [50% GDP per capita])**							
Standard of care	8.4	2950	92.4	56.960	25.106	550	*Reference*
HR‐HIVE*–*1‐dose	7.6	2650	92.9	57.273	25.234	525	cost‐saving^b^
HR‐HIVE*–*2‐doses	7.4	2600	92.9	57.320	25.252	532	367
HIVE*–*1‐dose	7.4	2600	92.9	57.322	25.255	540	dominated
HR‐HIVE‐Extended	7.1	2480	93.0	57.414	25.286	545	387^c^
HIVE*–*2‐doses	7.1	2500	93.1	57.427	25.295	559	dominated
HIVE‐Extended	6.3	2210	93.3	57.645	25.374	589	497^d^
**South Africa (CET: ICER < $1200/YLS [20% GDP per capita], ICER < $3001 [50% GDP per capita])**							
Standard of care	2.8	10,240	97.6	68.270	28.274	249	*Reference*
HR‐HIVE*–*1‐dose	2.5	9100	97.7	68.417	28.325	235	cost‐saving^b^
HR‐HIVE*–*2‐doses	2.4	8980	97.7	68.435	28.331	238	dominated
HR‐HIVE‐Extended	2.4	8830	97.7	68.454	28.337	240	377
HIVE*–*1‐dose	2.3	8440	97.8	68.502	28.355	254	dominated
HIVE*–*2‐doses	2.1	7810	97.9	68.592	28.386	267	dominated
HIVE‐Extended	1.9	6990	97.9	68.700	28.421	279	464^c^, ^d^
**Zimbabwe (CET: ICER < $293/YLS [20% GDP per capita], ICER < $732 [50% GDP per capita])**							
Standard of care	6.3	3110	95.0	65.837	27.158	411	*Reference*
HR‐HIVE*–*1‐dose	5.7	2810	95.3	66.119	27.260	390	cost‐saving^b^, ^c^
HR‐HIVE*–*2‐doses	5.6	2760	95.3	66.163	27.275	396	367
HIVE*–*1‐dose	5.4	2700	95.4	66.227	27.298	414	dominated
HR‐HIVE‐Extended	5.3	2650	95.4	66.255	27.304	407	389
HIVE*–*2‐doses	5.1	2530	95.6	66.386	27.353	440	dominated
HIVE‐Extended	4.1	2020	95.9	66.792	27.484	489	455^d^

Notes: Paediatric cumulative HIV incidence and 5‐year survival are rounded to the nearest tenth of a percent. Number of children acquiring HIV is rounded to the nearest ten. Undiscounted and discounted life expectancies are rounded to the nearest thousandth. Costs are rounded to the nearest dollar and are presented in 2019 USD. Discounted values are discounted at 3% per year. Incremental cost‐effectiveness ratios (ICERs) are rounded to the nearest dollar and are calculated using unrounded discounted life expectancy and discounted costs. The most cost‐effective broadly neutralizing antibody (bNAb) implementation strategy was the strategy that offered the greatest increase in overall population life expectancy while still having an ICER less than the cost‐effectiveness threshold when compared to the next best‐performing, non‐dominated strategy. A strategy is dominated if it offers less clinical benefit than another strategy with a lower cost, or if it has a higher ICER than another strategy with greater clinical benefit.

Abbreviations: %, percent; CET, cost‐effectiveness threshold; HIVE, all HIV‐exposed infants; HR, high‐risk HIV‐exposed infants; *n*, number; YLS, year of life saved.

^a^ Scaled to the size of the population of known HIV‐exposed children born annually in each country.

^b^ The label cost‐saving refers to a strategy that is less costly, but more clinically effective than the next non‐dominated comparator.

^c^ Indicates the most cost‐effective strategy at a cost‐effectiveness threshold of 20% GDP per capita.

^d^ Indicates the most cost‐effective strategy at a cost‐effectiveness threshold of 50% GDP per capita.

In all three countries, bNAb strategies would result in slightly higher 5‐year survival and longer life expectancy among the whole population of all known HIV‐exposed children compared with *SOC* due to HIV infections averted (Table 2). Undiscounted life expectancy among all known HIV‐exposed children would increase by 0.31–0.69 years (Côte d'Ivoire), 0.15–0.43 years (South Africa) and 0.28–0.95 years (Zimbabwe). Projected life expectancy gains with bNAb strategies would be greater for the sub‐cohort of high‐risk infants: 0.71–1.03 years (Côte d'Ivoire), 0.77–0.97 years (South Africa) and 1.15–1.70 years (Zimbabwe) (Table A7).

### 3.2 Cost‐effectiveness of implementation strategies

Compared to *SOC*, *HR‐HIVE–1‐dose* would increase life expectancy and reduce costs in all three countries (Table 2). At the 20% GDP per capita cost‐effectiveness threshold (Côte d'Ivoire: $455/YLS, South Africa: $1200/YLS, Zimbabwe: $293/YLS), the cost‐effective bNAb implementation strategy differs by country: *HR‐HIVE‐Extended* in Côte d'Ivoire (ICER: $387/YLS), *HIVE‐Extended* in South Africa (ICER: $464/YLS) and *HR‐HIVE–1‐dose* in Zimbabwe (cost‐saving). At the 50% GDP per capita threshold (Côte d'Ivoire: $1138/YLS, South Africa: $3001/YLS, Zimbabwe: $732/YLS), *HIVE‐Extended* is cost‐effective in all three settings, with ICERs of $497/YLS in Côte d'Ivoire, $464/YLS in South Africa and $455/YLS in Zimbabwe, each compared to the next non‐dominated strategy.

### 3.3 Univariate sensitivity analyses

Throughout the ranges examined, bNAb strategies would remain cost‐effective in all three settings at both cost‐effectiveness thresholds, except when bNAb efficacy is reduced to 10% (Tables A8–A36). For *HIVE‐Extended* to retain cost‐effectiveness, bNAb efficacy among non‐high‐risk infants would need to closely resemble base case bNAb efficacy among high‐risk infants in Côte d'Ivoire and Zimbabwe; however, in South Africa, *HIVE‐Extended* would remain cost‐effective if even bNAb efficacy among non‐high‐risk infants were to drop to as low as 20% (Figure A3). Using the 20% GDP per capita threshold, the cost‐effective strategy changes from that of the base case in at least two countries when bNAb efficacy is ≤30%; bNAb cost is ≥$120/dose; or when bNAb effect duration, postnatal transmission risk, maternal retention in care or ART cost is varied (Tables A8–A36). For example, when the bNAb effect duration is reduced to 1 month, the bNAbs programme value would diminish; the most cost‐effective strategy would become *HR‐HIVE–1‐dose* in Côte d'Ivoire and Zimbabwe and *HR‐HIVE–2‐doses* in South Africa (Table A13).

When the bNAb effect duration is lengthened to 6 months (with every 6 months dosing frequency), *HIVE‐Extended* would become cost‐effective in all settings at both thresholds. Aside from scenarios in which bNAb efficacy is ≤40%, bNAb cost ≥$80/dose, bNAb effect duration is reduced to 1 month or postnatal vertical transmission risk is halved, *HIVE‐Extended* remains consistently cost‐effective in all settings using the 50% GDP per capita threshold.

### 3.4 Multivariate sensitivity analyses

**Figure 3:**
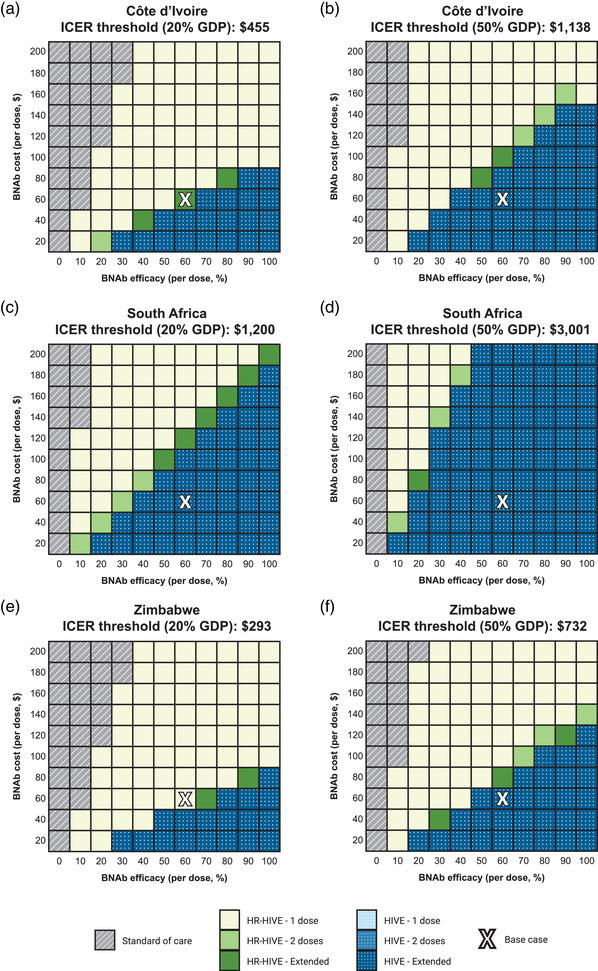
We conducted a multivariate sensitivity analysis of the impact of bNAb cost per dose (X‐axis) and bNAb efficacy per dose (Y‐axis) on the most cost‐effective bNAb implementation strategy in each country setting at specified cost‐effectiveness thresholds. The most cost‐effective bNAb implementation strategy was the strategy that offered the greatest increase in overall population life expectancy while still having an ICER less than the cost‐effectiveness threshold when compared to the next best‐performing, non‐dominated strategy. Given the ongoing debate regarding the cost‐effectiveness threshold at which interventions are affordable in resource‐limited settings, we examined outcomes at both 20% GDP per capita (left panels) and 50% per GDP per capita (right panels) cost‐effectiveness thresholds in the three country settings examined: Côte d'Ivoire (Panels a and b), South Africa (Panels c and d) and Zimbabwe (Panels e and f). The white “X” in each panel reflects the base‐case assumptions of 60% bNAb efficacy at a cost of $60 per dose. Abbreviations: bNAb, broadly neutralizing antibody; GDP, gross domestic product; HIVE, all HIV‐exposed infants; HR‐HIVE, high‐risk HIV‐exposed infants; ICER, incremental cost‐effectiveness ratio.

BNAb strategies are cost‐effective throughout a broad range of bNAb costs and efficacy in all three countries (Table A38 and Figure 3). In Côte d'Ivoire, bNAb strategies are cost‐effective at all modelled costs ($20–$200/dose) if efficacy is ≥40% or ≥20% using a 20% or 50% GDP per capita threshold, respectively (Figure 3, Panels a and b). In South Africa, bNAbs strategies are cost‐effective at all tested costs if efficacy is ≥20% or ≥10% using a 20% or 50% GDP per capita threshold, respectively (Panels C and D). In Zimbabwe, bNAbs strategies are cost‐effective at all tested costs if efficacy is ≥40% or ≥30% using a 20% or 50% GDP per capita threshold, respectively (Panels E and F). If bNAb efficacy rose to 80–100% as *in vitro* data suggest, then the *HIVE‐Extended* strategy would be cost‐effective in all three settings at costs of $60/dose or $100/dose using a 20% or 50% GDP per capita threshold, respectively (Figure 3).

## 4 DISCUSSION

In this model‐based analysis, we project that offering bNAb prophylaxis to infants known to be HIV exposed could reduce cumulative HIV incidence by 0.3–2.2% and would be cost‐effective when added to existing HIV prevention strategies in Côte d'Ivoire, South Africa and Zimbabwe. Our projections suggest that when considering the substantial lifetime HIV‐related costs and poor health outcomes averted by preventing vertical HIV transmission, bNAb strategies are likely to be cost‐effective in sub‐Saharan African settings such as these, even if bNAb efficacy is as low as 20–40%. Although identifying high‐risk HIV‐exposed infants may pose operational challenges, offering these infants a birth dose of a bNAb with 60% efficacy, 3‐month effect duration and $60 cost would likely be cost‐saving in all three settings, compared to oral prophylaxis without a bNAb.

While infant bNAb prophylaxis is likely to be cost‐effective, we found that the optimal target population and dosing approach depended upon the specific care cascade vulnerabilities in each setting. In Côte d'Ivoire and Zimbabwe, fewer than 95% of women with known HIV infection receive ART in pregnancy, so overall transmission risks are highest in the intrapartum and early postnatal periods [1]. Prioritizing a bNAb birth dose for high‐risk HIV‐exposed infants offers greater relative value in these settings than in South Africa, where 98% of women with known HIV receive ART in pregnancy [1]. With better ART uptake in pregnancy, the highest transmission risks for the overall population in South Africa are shifted towards the mid‐to‐late postnatal period when maternal disengagement from care and ART non‐adherence become more prevalent among mothers of high‐risk and non‐high‐risk infants alike.

Resource constraints, partially reflected by GDP per capita, also play a key role in determining the cost‐effectiveness of bNAb implementation strategies. Administering bNAbs throughout breastfeeding to all known HIV‐exposed infants offers the greatest clinical benefit, with ICERs comparable to other HIV prevention interventions currently funded by international donors and below the 50% GDP per capita threshold in all three settings [67, 68, 69]. Without donor contributions, a 20% GDP per capita threshold may be more consistent with a willingness to pay for health in low‐ and middle‐income countries (see online Appendix; Table A3). At this threshold, the cost‐effective strategy in Côte d'Ivoire and Zimbabwe instead only offers bNAbs to high‐risk infants; more expansive programmes ceased to be cost‐effective under base‐case assumptions. This finding suggests that increased investment may be necessary to meet the global commitment to eliminate vertical transmission of HIV [1].

The optimal bNAb implementation approach depends upon the efficacy, effect duration and costs of the specific product, all of which remain uncertain. Although VRC01 was not effective at preventing overall sexual HIV acquisition in the AMP studies, it did prevent infection with susceptible virus [6]. There are several bNAb products in development with much higher potency and longer half‐life than VRC01; they may provide improved efficacy and effect duration [10, 70]. Effective HIV neutralization likely will require multiple bNAbs in combination [10], which could increase production costs. However, we found that bNAb strategies would remain cost‐effective even if costs tripled from base‐case assumptions. For a strategy offering all children known to be HIV‐exposed bNAbs throughout breastfeeding to be cost‐effective in a variety of settings, target bNAb product characteristics would broadly include a 3‐month or longer duration of effect, cost of $60 or less per dose and efficacy of at least 50%.

Beyond cost‐effectiveness, other crucial logistic considerations may impact bNAb programmes. For example, implementing a national bNAb programme would require training providers in maternal–child health clinics to give subcutaneous injections, monitor for adverse reactions and counsel patients about using bNAbs alongside existing approaches to vertical HIV transmission prevention. Alternatively, bNAbs could be delivered through scaled‐up Expanded Programme on Immunization services where similar injections are already routinely administered and vaccine cold chain systems are in place. Operationally, accurate identification of high‐risk HIV‐exposed infants is challenging [71], so bNAb strategies that focus on high‐risk infants would likely fail to provide bNAb prophylaxis to many eligible infants, unless systems to identify these infants are improved. The rollout of a bNAb programme would also require substantial political will and public health messaging campaigns. However, the implementation of hepatitis B virus (HBV) immunoglobulin at birth to HBV‐exposed infants in many resource‐limited settings could provide a blueprint for bNAb implementation [2].

Our analysis has several limitations. First, bNAb product characteristics remain uncertain, as while there are several promising candidates in the late stages of development, no bNAbs have yet been brought to market [7, 8, 10, 11]. However, based on our projections, bNAbs would be cost‐effective in sub‐Saharan settings similar to the three we evaluated throughout a broad range of plausible costs and efficacy assumptions. Second, we assumed that present‐day maternal and infant HIV care cascade characteristics and recommended antiretroviral regimens would be static over time. Improvements in ART coverage in pregnancy, ART adherence, or postpartum retention in care, or implementation of more effective regimens for treatment and prophylaxis, such as emerging long‐acting antiretrovirals, could reduce the potential added value of bNAb prophylaxis. Absent these improvements, we found that even if bNAbs were offered to all children known to be HIV exposed throughout breastfeeding, vertical HIV transmission rates would remain unacceptably high. Renewed efforts to promote early HIV diagnosis, prompt treatment initiation and engagement in the care of pregnant and lactating women are also needed to eliminate vertical HIV transmission. However, our results suggest that bNAbs would be an impactful and cost‐effective addition to a portfolio of interventions needed to achieve the elimination of vertical transmission. Lastly, we did not consider universal strategies involving offering bNAbs to all infants born in high‐burden settings, irrespective of known HIV exposure at birth, and, therefore, did not assess the potential impact of reducing vertical transmission related to unrecognized maternal HIV infection or acute maternal HIV infection postpartum. In settings where knowledge of maternal serostatus is low, or acute HIV infection during pregnancy or breastfeeding is common, offering bNAbs to all infants at birth might offer good value.

## 5 CONCLUSIONS

Throughout a wide range of plausible cost and efficacy assumptions, bNAb prophylaxis for HIV‐exposed children in various sub‐Saharan African settings would be a cost‐effective intervention to prevent vertical HIV transmission. Clinical studies of the pharmacokinetics, safety, tolerability and potential efficacy of novel bNAb products for infant prophylaxis should be prioritized.

## COMPETING INTERESTS

SRP is a consultant for Merck, Moderna, Pfizer and Dynavax and has a sponsored research programme with Moderna in the area of cytomegalovirus vaccines. All other authors declare that they have no competing interests.

## AUTHORS’ CONTRIBUTIONS

CMD, ALC, OU and CA designed and conducted the analyses. CMD, OU, CA, MCW, KAF and ALC interpreted model results. SRP, LSC, CKC, GGF, LM, VL and EJM provided data for use in the model‐based analyses. CMD, OU, CA, MCW, KAF and ALC designed updates to the CEPAC model to facilitate the analysis. CMD, OU and CA drafted the manuscript. All authors critically revised the manuscript and approved its submission.

## FUNDING

This work was supported by the Eunice Kennedy Shriver National Institute of Child Health and Human Development [K08HD101342 to CMD, R01HD079214 to ALC and MCW] and the International Maternal Pediatric Adolescent AIDS Clinical Trials Network [DR808 to CMD and ALC]. Overall support for the International Maternal Pediatric Adolescent AIDS Clinical Trials Network (IMPAACT) was provided by the National Institute of Allergy and Infectious Diseases (NIAID) with co‐funding from the *Eunice Kennedy Shriver* National Institute of Child Health and Human Development (NICHD) and the National Institute of Mental Health (NIMH); all components of the National Institutes of Health (NIH), under Award Numbers UM1AI068632 (IMPAACT LOC), UM1AI068616 (IMPAACT SDMC) and UM1AI106716 (IMPAACT LC); and by NICHD contract number HHSN275201800001I. CKC was supported, in part, by the Duke University Center for AIDS Research [5P30 AI064518].

## DISCLAIMER

The content is solely the responsibility of the authors and does not necessarily represent the official views of the NIH. The funding sources had no role in the study's design, analysis or interpretation, nor were they involved in the manuscript writing process or decision to submit for publication.

## Supporting information


**Table A1**: Literature review on the efficacy of VRC01 against infant transmitted/founder viruses.
**Table A2**: Itemized costs included in modeled bNAb cost/dose.
**Table A3**: Cost‐effectiveness thresholds for LMICs proposed in published literature Cost‐effectiveness threshold (in % GDP per capita).
**Table A4**: Consolidated Health Economic Evaluation Reporting Standards (CHEERS) checklist.
**Table A5**: Extended model input parameters.
**Table A6**: Comparison of CEPAC‐P infant HIV incidence projections with other nationally representative published estimates.
**Table A7**: Clinical and economic outcomes of bNAb administration program, by infant risk status.
**Table A8**: Scenario analysis: bNAb does not reduce intrapartum transmission (base case: 60% reduction).
**Table A9**: Scenario analysis: intrapartum transmissions accounts for 67% of all perinatal transmissions (base case: 33%).
**Table A10**: Scenario analysis: proportion of high‐risk infants recognized as being high‐risk (base case: 100%).
**Table A11**: One‐way sensitivity analysis: bNAb efficacy against intrapartum and postpartum transmission.
**Table A12**: One‐way sensitivity analysis: bNAb cost.
**Table A13**: One‐way sensitivity analysis: bNAb effect duration.
**Table A14**: One‐way sensitivity analysis: bNAb toxicity.
**Table A15**: One‐way sensitivity analysis: bNAb uptake.
**Table A16**: One‐way sensitivity analysis: proportion of mothers on antiretroviral therapy during pregnancy.
**Table A17**: One‐way sensitivity analysis: proportion of mothers with viral load <1,000 copies/mL at delivery.
**Table A18**: One‐way sensitivity analysis: perinatal vertical transmission risk.
**Table A19**: One‐way sensitivity analysis: breastfeeding duration.
**Table A20**: One‐way sensitivity analysis: postpartum vertical transmission risk.
**Table A21**: One‐way sensitivity analysis: postpartum maternal retention in care.
**Table A22**: One‐way sensitivity analysis: postpartum maternal virologic suppression.
**Table A23**: One‐way sensitivity analysis: antiretroviral therapy treatment cost.
**Table A24**: One‐way sensitivity analysis: infant oral prophylaxis adherence.
**Table A25**: One‐way sensitivity analysis: infant oral prophylaxis cost.
**Table A26**: One‐way sensitivity analysis: infant oral prophylaxis efficacy.
**Table A27**: One‐way sensitivity analysis: infant oral prophylaxis major toxicity leading to discontinuation.
**Table A28**: One‐way sensitivity analysis: birth early infant diagnosis uptake.
**Table A29**: One‐way sensitivity analysis: six to eight week early infant diagnosis uptake.
**Table A30**: One‐way sensitivity analysis: six/nine month early infant diagnosis uptake.
**Table A31**: One‐way sensitivity analysis: 18 month early infant diagnosis uptake.
**Table A32**: One‐way sensitivity analysis: overall early infant diagnosis uptake.
**Table A33**: One‐way sensitivity analysis: early infant diagnosis result return rate, return time, & linkage to care.
**Table A34**: One‐way sensitivity analysis: bNAb reduction in nucleic acid amplification test sensitivity.
**Table A35**: One‐way sensitivity analysis: bNAb reduction in antibody test specificity (stop bNAb if positive).
**Table A36**: One‐way sensitivity analysis: bNAb reduction in antibody test specificity (continue bNAb if positive).
**Table A37**: One‐way sensitivity analysis: 1st‐line pediatric antiretroviral therapy efficacy.
**Table A38**: Minimum bNAb efficacy required for cost‐effectiveness (50% GDP per capita) of a HIVE‐Extended strategy for a variety of product characteristics across all 3 settings.
**Figure A1**: CEPAC‐P infant postnatal HIV prophylaxis module flowchart.
**Figure A2**: Cost‐effectiveness frontier of hybrid bNAb administration strategies.
**Figure A3**: The influence of bNAb efficacy for non‐high risk infants on the ICER value of the *HIVE‐Extended* strategy (highrisk bNAb efficacy = 60%).

## Data Availability

Model input data and CEPAC‐generated sensitivity analysis data that support the findings of this study are outlined in detail in the supplementary material of this article. Additional data that support the findings of this study, including detailed model specifications, are available from the corresponding author upon reasonable request.

## References

[1] Joint United Nations Programme on HIV/AIDS (UNAIDS) .UNAIDS Data 2020. UNAIDS; 2020. 12349391

[2] Van De Perre P , Goga A , Ngandu N , Nagot N , Moodley D , King R , et al. Eliminating postnatal HIV transmission in high incidence areas: need for complementary biomedical interventions. Lancet. 2021;397:1316–24. 33812490 10.1016/S0140-6736(21)00570-5

[3] Hessell AJ , Jaworski JP , Epson E , Matsuda K , Pandey S , Kahl C , et al. Early short‐term treatment with neutralizing human monoclonal antibodies halts SHIV infection in infant macaques. Nat Med. 2016;22:362–8. 26998834 10.1038/nm.4063 PMC4983100

[4] Rosenberg YJ , Jiang X , Cheever T , Coulter FJ , Pandey S , Sack M , et al. Protection of newborn macaques by plant‐derived HIV broadly neutralizing antibodies: a model for passive immunotherapy during breastfeeding. J Virol. 2021;95(18):e0026821. 34190597 10.1128/JVI.00268-21 PMC8387040

[5] Shapiro MB , Cheever T , Malherbe DC , Pandey S , Reed J , Yang ES , et al. Single‐dose bNAb cocktail or abbreviated ART post‐exposure regimens achieve tight SHIV control without adaptive immunity. Nat Commun. 2020;11:70. 31911610 10.1038/s41467-019-13972-y PMC6946664

[6] Corey L , Gilbert PB , Juraska M , Montefiori DC , Morris L , Karuna ST , et al. Two randomized trials of neutralizing antibodies to prevent HIV‐1 acquisition. N Engl J Med. 2021;384:1003–4. 33730454 10.1056/NEJMoa2031738 PMC8189692

[7] Cunningham C . Safety and PK of potent anti‐HIV monoclonal Ab VRC07‐523LS in HIV‐exposed infants (Oral Abstract 03.02). 2021.

[8] Mcfarland EJ , Cunningham CK , Muresan P , Capparelli EV , Perlowski C , Morgan P , et al. Safety, tolerability, and pharmacokinetics of a long‐acting broadly neutralizing HIV‐1 monoclonal antibody VRC01LS in HIV‐1‐exposed newborn infants. J Infect Dis. 2021;224:1916–24. 34009371 10.1093/infdis/jiab229 PMC8643399

[9] Miner MD , Corey L , Montefiori D . Broadly neutralizing monoclonal antibodies for HIV prevention. J Int AIDS Soc. 2021;24(Suppl 7):e25829. 34806308 10.1002/jia2.25829 PMC8606861

[10] Lorenzi JCC , Mendoza P , Cohen YZ , Nogueira L , Lavine C , Sapiente J , et al. Neutralizing activity of broadly neutralizing anti‐HIV‐1 antibodies against primary African isolates. J Virol. 2020;95:e01909–20. 33298542 10.1128/JVI.01909-20 PMC8092834

[11] Stefic K , Bouvin‐Pley M , Essat A , Visdeloup C , Moreau A , Goujard C , et al. Sensitivity to broadly neutralizing antibodies of recently transmitted HIV‐1 Clade CRF02_AG viruses with a focus on evolution over time. J Virol. 2019;93:e01492–18. 30404804 10.1128/JVI.01492-18 PMC6321924

[12] Anderson DJ , Politch JA , Zeitlin L , Hiatt A , Kadasia K , Mayer KH , et al. Systemic and topical use of monoclonal antibodies to prevent the sexual transmission of HIV. AIDS. 2017;31:1505–17. 28463876 10.1097/QAD.0000000000001521 PMC5619647

[13] Cunningham CK , Mcfarland EJ , Morrison RL , Capparelli EV , Safrit JT , Mofenson LM , et al. Safety, tolerability, and pharmacokinetics of the broadly neutralizing human immunodeficiency virus (HIV)‐1 monoclonal antibody VRC01 in HIV‐exposed newborn infants. J Infect Dis. 2020;222:628–36. 31681963 10.1093/infdis/jiz532 PMC7377284

[14] Ciaranello AL , Morris BL , Walensky RP , Weinstein MC , Ayaya S , Doherty K , et al. Validation and calibration of a computer simulation model of pediatric HIV infection. PLoS One. 2013;8:e83389. 24349503 10.1371/journal.pone.0083389 PMC3862684

[15] Frank SC , Cohn J , Dunning L , Sacks E , Walensky RP , Mukherjee S , et al. Clinical effect and cost‐effectiveness of incorporation of point‐of‐care assays into early infant HIV diagnosis programmes in Zimbabwe: a modelling study. Lancet HIV. 2019;6:e182–90. 30737187 10.1016/S2352-3018(18)30328-X PMC6408227

[16] Dunning L , Gandhi AR , Penazzato M , Soeteman DRI , Revill P , Frank S , et al. Optimizing infant HIV diagnosis with additional screening at immunization clinics in three sub‐Saharan African settings: a cost‐effectiveness analysis. J Int AIDS Soc. 2021;24:e25651. 33474817 10.1002/jia2.25651 PMC8992471

[17] Husereau D , Drummond M , Petrou S , Carswell C , Moher D , Greenberg D , et al. Consolidated Health Economic Evaluation Reporting Standards (CHEERS) statement. BMJ. 2013;346:f1049. 23529982 10.1136/bmj.f1049

[18] World Health Organization . Consolidated guidelines on the use of antiretroviral drugs for the treating and preventing HIV infection: recommendations for a public health approach. 2nd ed. 2016 [cited 2020 Jul 23] https://apps.who.int/iris/handle/10665/208825. 27466667

[19] Egbe TO , Tazinya R‐MA , Halle‐Ekane GE , Egbe E‐N , Achidi EA . Estimating HIV incidence during pregnancy and knowledge of prevention of mother‐to‐child transmission with an ad hoc analysis of potential cofactors. J Pregnancy. 2016;2016:7397695. 27127653 10.1155/2016/7397695 PMC4830744

[20] Imade GE , Sagay AS , Musa J , Ocheke AN , Adeniyi DS , Idighri M , et al. Declining rate of infection with maternal human immunodeficiency virus at delivery units in north‐central Nigeria. Afr J Reprod Health. 2013;17:138–45. 24689325

[21] Dinh T‐H , Delaney KP , Goga A , Jackson D , Lombard C , Woldesenbet S , et al. Impact of maternal HIV seroconversion during pregnancy on early mother to child transmission of HIV (MTCT) measured at 4–8 weeks postpartum in South Africa 2011–2012: a national population‐based evaluation. PLoS One. 2015;10:e0125525. 25942423 10.1371/journal.pone.0125525 PMC4420458

[22] Mbizvo MT , Kasule J , Mahomed K , Nathoo K . HIV‐1 seroconversion incidence following pregnancy and delivery among women seronegative at recruitment in Harare, Zimbabwe. Cent Afr J Med. 2001;47:115–8. 11921668 10.4314/cajm.v47i5.8600

[23] Morrison CS , Wang J , Van Der Pol B , Padian N , Salata RA , Richardson BA . Pregnancy and the risk of HIV‐1 acquisition among women in Uganda and Zimbabwe. AIDS. 2007;21:1027–34. 17457097 10.1097/QAD.0b013e3280f00fc4

[24] Teasdale CA , Abrams EJ , Chiasson MA , Justman J , Blanchard K , Jones HE . Incidence of sexually transmitted infections during pregnancy. PLoS One. 2018;13:e0197696. 29795625 10.1371/journal.pone.0197696 PMC5967814

[25] Elizabeth Glaser Pediatric AIDS Foundation . The Elizabeth Glaser Pediatric AIDS Foundation ‐ Zimbabwe Annual Report, January–December 2018. Elizabeth Glaser Pediatric AIDS Foundation; 2019.

[26] South Africa National Department of Health . National Antenatal Sentinel HIV Survey Key Findings. South Africa: 2017.

[27] Desmonde S , Bangali M , Amorissani‐Folquet M , Lenaud S , Karcher S , Lohoues‐Kouacou M , et al. Effectiveness of a web‐based information system to improve HIV early diagnosis and hepatitis B immunization coverages in Abidjan, Cote d'Ivoire. The DEPISTNEO Project. 2019 [cited 2022 Aug 18] https://academicmedicaleducation.com/meeting/international‐workshop‐hiv‐pediatrics‐2019/abstract/effectiveness‐web‐based‐information.

[28] de Beer S , Kalk E , Kroon M , Boulle A , Osler M , Euvrard J , et al. A longitudinal analysis of the completeness of maternal HIV testing, including repeat testing in Cape Town, South Africa. J Int AIDS Soc. 2020;23:e25441. 31997583 10.1002/jia2.25441 PMC6989397

[29] Heemelaar S , Habets N , Makukula Z , Roosmalen J , van den Akker T . Repeat HIV testing during pregnancy and delivery: missed opportunities in a rural district hospital in Zambia. Trop Med Int Health. 2015;20:277–83. 25418130 10.1111/tmi.12432

[30] Rogers AJ , Akama E , Weke E , Blackburn J , Owino G , Bukusi EA , et al. Implementation of repeat HIV testing during pregnancy in southwestern Kenya: progress and missed opportunities. J Int AIDS Soc. 2017;20:e25036. 29236362 10.1002/jia2.25036 PMC5810348

[31] Mandala J , Kasonde P , Badru T , Dirks R , Torpey K . HIV retesting of HIV‐negative pregnant women in the context of prevention of mother‐to‐child transmission of HIV in primary health centers in rural Zambia: what did we learn? J Int Assoc Provid AIDS Care. 2019;18:1–6. 10.1177/2325958218823530 PMC6748466 30798664

[32] Zimbabwe Ministry of Health and Child Care (MOHCC) . Zimbabwe Population‐Based HIV Impact Assessment (ZIMPHIA) 2020: Summary Sheet. Harare: 2020.

[33] Moyo F , Mazanderani AH , Murray T , Sherman GG , Kufa T . Achieving maternal viral load suppression for elimination of mother‐to‐child transmission of HIV in South Africa. AIDS. 2021;35:307–16. 33394673 10.1097/QAD.0000000000002733

[34] ICAP at Columbia University . Cote d'Ivoire Population‐Based HIV Impact Assessment (CIPHIA) 2017–2018: Summary Sheet. Population‐based HIV Impact Assessment (PHIA) Project. 2018 [cited 2021 Aug 27] https://phia.icap.columbia.edu/wp‐content/uploads/2018/08/CIPHIA_Cote‐DIvoire‐SS_FINAL.pdf.

[35] Ministère du Plan et du Développement . La Situation des Femmes et des Enfants en Côte d'Ivoire ‐ Enquête à Indicateurs Multiples 2016 ‐ MICS5. 2016 [cited 2022 Oct 6] https://mics‐surveys‐prod.s3.amazonaws.com/MICS5/West%20and%20Central%20Africa/C%C3%B4te%20d%27Ivoire/2016/Final/Cote%20d%27Ivoire%202016%20MICS_French.pdf.

[36] South Africa National Department of Health . South Africa Demographic and Health Survey 2016. 2019 [cited 2022 Oct 6] https://dhsprogram.com/pubs/pdf/FR337/FR337.pdf.

[37] Zimbabwe National Statistics Agency . Demographic and Health Survey 2015. Harare: 2016.

[38] Stover J , Glaubius R , Kassanjee R , Dugdale C . Updates to the Spectrum/AIM model for the UNAIDS 2020 HIV estimates. J Int AIDS Soc. 2021;24:e25778. 34546648 10.1002/jia2.25778 PMC8454674

[39] Dinh T , Mushavi A , Balachandra S , Barr BT , Balachandra S , Shambira G , et al. Impact of option B+ and maternal HIV RNA viral load on mother‐to‐child HIV transmission: findings from an 18‐month prospective cohort study of a nationally representative sample of mother–infant pairs, Zimbabwe 2016–2017. 2018 [cited 2021 Aug 18] http://programme.aids2018.org/Abstract/Abstract/6374.

[40] Nguyen K , Abrams EJ , Brittain K , Phillips TK , Ronan A , Zerbe A , et al. Breastfeeding cessation, maternal adherence to antiretroviral therapy, and HIV viremia in the early postpartum period: a prospective cohort study. 8th International Workshop on HIV Pediatrics. Abstract P100:99–100 [cited 2020 Jul 14] http://www.infectiousdiseasesonline.com/wp‐content/uploads/2016/07/8th‐HIVPediatrics_abstractbook_web.pdf.

[41] Watt MH , Cichowitz C , Kisigo G , Minja L , Knettel BA , Knippler ET , et al. Predictors of postpartum HIV care engagement for women enrolled in prevention of mother‐to‐child transmission (PMTCT) programs in Tanzania. AIDS Care. 2019;31:687–98. 30466304 10.1080/09540121.2018.1550248 PMC6443456

[42] Myer L , Phillips TK , Zerbe A , Brittain K , Lesosky M , Hsiao N‐Y , et al. Integration of postpartum healthcare services for HIV‐infected women and their infants in South Africa: a randomised controlled trial. PLoS Med. 2018;15:e1002547. 29601570 10.1371/journal.pmed.1002547 PMC5877834

[43] Harrington BJ , Pence BW , Maliwichi M , Jumbe AN , Gondwe NA , Wallie SD , et al. Probable antenatal depression at antiretroviral initiation and postpartum viral suppression and engagement in care. AIDS. 2018;32:2827–33. 30234603 10.1097/QAD.0000000000002025 PMC6528829

[44] Luoga E , Vanobberghen F , Bircher R , Nyuri A , Ntamatungiro AJ , Mnzava D , et al. Brief report: no HIV transmission from virally suppressed mothers during breastfeeding in rural Tanzania. J Acquir Immune Defic Syndr. 2018;79:e17–20. 29781882 10.1097/QAI.0000000000001758

[45] Gill MM , Hoffman HJ , Ndatimana D , Mugwaneza P , Guay L , Ndayisaba GF , et al. 24‐month HIV‐free survival among infants born to HIV‐positive women enrolled in Option B+ program in Kigali, Rwanda: the Kabeho Study. Medicine. 2017;96:e9445. 29390577 10.1097/MD.0000000000009445 PMC5758279

[46] Hosseinipour M , Nelson JAE , Trapence C , Rutstein SE , Kasende F , Kayoyo V , et al. Viral suppression and HIV drug resistance at 6 months among women in Malawi's Option B+ program: results from the PURE Malawi Study. J Acquir Immune Defic Syndr. 2017;75(Suppl 2):S149–55. 28498184 10.1097/QAI.0000000000001368 PMC5431274

[47] Onoya D , Sineke T , Brennan AT , Long L , Fox MP . Timing of pregnancy, postpartum risk of virologic failure and loss to follow‐up among HIV‐positive women. AIDS. 2017;31:1593–602. 28463877 10.1097/QAD.0000000000001517 PMC5491237

[48] Davis NL , Miller WC , Hudgens MG , Chasela CS , Sichali D , Kayira D , et al. Maternal and breastmilk viral load: impacts of adherence on peripartum HIV infections averted–The Breastfeeding, Antiretrovirals, and Nutrition Study. J Acquir Immune Defic Syndr. 2016;73:572–80. 27846071 10.1097/QAI.0000000000001145 PMC5141681

[49] Dugdale C . A meta‐analysis of viral load‐based vertical HIV transmission risks. 2020 [cited 2021 Jun 16] https://www.epidem.org/modelling‐paediatric‐hiv‐and‐the‐need‐for‐art‐october‐2020.

[50] Stover J , Glaubius R , Mofenson L , Dugdale CM , Davies M‐A , Patten G , et al. Updates to the Spectrum/AIM model for estimating key HIV indicators at national and subnational levels. AIDS. 2019;33(Suppl 3):S227–34. 31805028 10.1097/QAD.0000000000002357 PMC6919230

[51] Zimbabwe National Statistics Agency (ZIMSTAT), UNICEF . Zimbabwe Multiple Indicator Cluster Survey 2019, Survey Findings Report. Harare: ZIMSTAT and UNICEF; 2019.

[52] McCoy S , Koyuncu A , Kang‐Dufour M , Mushavi A , Mahomva A , Padian N , et al. Approaching eMTCT in Zimbabwe: expansion of PMTCT services and declining MTCT, 2012–2018. In: IAS 2019: Abstract #TUPEC473. Mexico City, MX: International AIDS Society; 2019.

[53] Zimbabwe Ministry of Health and Child Care (MOHCC) .Zimbabwe Population‐Based HIV Impact Assessment (ZIMPHIA) 2015–2016: Final Report. Harare: 2019.

[54] UNICEF . Countdown to 2030 ‐ Countdown Country Dashboards. [cited 2022 Oct 6] https://www.countdown2030.org/landing_page.

[55] West NS , Schwartz SR , Yende N , Schwartz SJ , Parmley L , Gadarowski MB , et al. Infant feeding by South African mothers living with HIV: implications for future training of health care workers and the need for consistent counseling. Int Breastfeed J. 2019;14:11. 30815026 10.1186/s13006-019-0205-1 PMC6376722

[56] Patel MR , Mushavi A , Balachandra S , Shambira G , Nyakura J , Mugurungi O , et al. HIV‐exposed uninfected infant morbidity and mortality within a nationally representative prospective cohort of mother–infant pairs in Zimbabwe. AIDS. 2020;34:1339–46. 32590432 10.1097/QAD.0000000000002567 PMC8900086

[57] Desmond AC , Moodley D , Conolly CA , Castel SA , Coovadia HM . Evaluation of adherence measures of antiretroviral prophylaxis in HIV exposed infants in the first 6 weeks of life. BMC Pediatr. 2015;15:23. 25885678 10.1186/s12887-015-0340-9 PMC4381484

[58] Chasela CS , Hudgens MG , Jamieson DJ , Kayira D , Hosseinipour MC , Kourtis AP , et al. Maternal or infant antiretroviral drugs to reduce HIV‐1 transmission. N Engl J Med. 2010;362:2271–81. 20554982 10.1056/NEJMoa0911486 PMC3440865

[59] Six Week Extended‐Dose Nevirapine (SWEN) Study Team T , Bedri A , Gudetta B , Isehak A , Kumbi S , Lulseged S , et al. Extended‐dose nevirapine to 6 weeks of age for infants to prevent HIV transmission via breastfeeding in Ethiopia, India, and Uganda: an analysis of three randomised controlled trials. Lancet. 2008;372:300–13. 18657709 10.1016/S0140-6736(08)61114-9

[60] Hudgens MG , Taha TE , Omer SB , Jamieson DJ , Lee H , Mofenson LM , et al. Pooled individual data analysis of 5 randomized trials of infant nevirapine prophylaxis to prevent breast‐milk HIV‐1 transmission. Clin Infect Dis. 2013;56:131–9. 22997212 10.1093/cid/cis808 PMC3518881

[61] The Global Fund . Price list. [cited 2020 Oct 27] https://public.tableau.com/profile/the.global.fund#!/vizhome/PQRPricelist_English/PriceList.

[62] COVAX Working Group on delivery costs . Costs of delivering COVID‐19 vaccine in 92 AMC countries. 2021 [cited 2021 Apr 23] https://www.who.int/publications/m/item/costs‐of‐delivering‐covid‐19‐vaccine‐in‐92‐amc‐countries.

[63] Mvundura M , Lorenson K , Chweya A , Kigadye R , Bartholomew K , Makame M , et al. Estimating the costs of the vaccine supply chain and service delivery for selected districts in Kenya and Tanzania. Vaccine. 2015;33:2697–703. 25865467 10.1016/j.vaccine.2015.03.084

[64] Cunnama L , Abrams EJ , Myer L , Phillips TK , Dugdale CM , Ciaranello AL , et al. Provider‐ and patient‐level costs associated with providing antiretroviral therapy during the postpartum phase to women living with HIV in South Africa: a cost comparison of three postpartum models of care. Trop Med Int Health. 2020;25:1553–67. 32959434 10.1111/tmi.13493 PMC7756215

[65] The World Bank . GDP per capita (current US$) ‐ South Africa, Zimbabwe, Cote d'Ivoire. 2020 [cited 2020 Sep 9] https://data.worldbank.org/indicator/NY.GDP.PCAP.CD?end=2019&locations=ZA‐ZW‐CI&start=2019&view=bar.

[66] Kumwenda NI , Hoover DR , Mofenson LM , Thigpen MC , Kafulafula G , et al. Extended antiretroviral prophylaxis to reduce breast‐milk HIV‐1 transmission. N Engl J Med. 2008;359:119–29. 18525035 10.1056/NEJMoa0801941

[67] Sarkar S , Corso P , Ebrahim‐Zadeh S , Kim P , Charania S , Wall K . Cost‐effectiveness of HIV prevention interventions in sub‐Saharan Africa: a systematic review. EClinicalMedicine. 2019;10:10–31. 31193863 10.1016/j.eclinm.2019.04.006 PMC6543190

[68] Karnon J , Orji N . Option B+ for the prevention of mother‐to‐child transmission of HIV infection in developing countries: a review of published cost‐effectiveness analyses. Health Policy Plan. 2016;31:1133–41. 27016949 10.1093/heapol/czw025

[69] Siedner MJ , Alba C , Fitzmaurice KP , Gilbert RF , Scott JA , Shebl FM , et al. Cost‐effectiveness of COVID‐19 vaccination in low‐ and middle‐income countries. J Infect Dis. 2022;226:1887–96. 35696544 10.1093/infdis/jiac243 PMC9214172

[70] Gama L , Koup RA . New‐generation high‐potency and designer antibodies: role in HIV‐1 treatment. Annu Rev Med. 2018;69:409–19. 29029583 10.1146/annurev-med-061016-041032

[71] Komtenza B , Satyanarayana S , Takarinda KC , Mukungunugwa SH , Mugurungi O , Chonzi P , et al. Identifying high or low risk of mother to child transmission of HIV: how Harare City, Zimbabwe is doing? PLoS One. 2019;14:e0212848. 30865646 10.1371/journal.pone.0212848 PMC6415877

