# Supplementary material for: Cost‐effectiveness of broadly neutralizing antibody prophylaxis for HIV‐exposed infants in sub‐Saharan African settings
Source: J Int AIDS Soc. 2023 Jan 5;26(1):e26052. doi: 10.1002/jia2.26052 (PMC9816086; doi:10.1002/jia2.26052)
Supplement: Supplementary file 1 — Table A1: Literature review on the efficacy of VRC01 against infant transmitted/founder viruses. Table A2: Itemized costs included in modeled bNAb cost/dose. Table A3: Cost‐effectiveness thresholds for LMICs proposed in published literature Cost‐effectiveness threshold (in % GDP per capita). Table A4: Consolidated Health Economic Evaluation Reporting Standards (CHEERS) checklist. Table A5: Extended model input parameters. Table A6: Comparison of CEPAC‐P infant HIV incidence projections with other nationally representative published estimates. Table A7: Clinical and economic outcomes of bNAb administration program, by infant risk status. Table A8: Scenario analysis: bNAb does not reduce intrapartum transmission (base case: 60% reduction). Table A9: Scenario analysis: intrapartum transmissions accounts for 67% of all perinatal transmissions (base case: 33%). Table A10: Scenario analysis: proportion of high‐risk infants recognized as being high‐risk (base case: 100%). Table A11: One‐way sensitivity analysis: bNAb efficacy against intrapartum and postpartum transmission. Table A12: One‐way sensitivity analysis: bNAb cost. Table A13: One‐way sensitivity analysis: bNAb effect duration. Table A14: One‐way sensitivity analysis: bNAb toxicity. Table A15: One‐way sensitivity analysis: bNAb uptake. Table A16: One‐way sensitivity analysis: proportion of mothers on antiretroviral therapy during pregnancy. Table A17: One‐way sensitivity analysis: proportion of mothers with viral load <1,000 copies/mL at delivery. Table A18: One‐way sensitivity analysis: perinatal vertical transmission risk. Table A19: One‐way sensitivity analysis: breastfeeding duration. Table A20: One‐way sensitivity analysis: postpartum vertical transmission risk. Table A21: One‐way sensitivity analysis: postpartum maternal retention in care. Table A22: One‐way sensitivity analysis: postpartum maternal virologic suppression. Table A23: One‐way sensitivity analysis: antiretroviral therapy treatment cost. Table A [file JIA2-26-e26052-s001.docx]

**Cost-effectiveness of broadly neutralizing antibody prophylaxis for HIV-exposed infants in sub-Saharan African settings**

**Appendix**

**Dugdale et al.**

Table of Contents

[**Appendix Methods** 3](#_Toc116039419)

[Cohort characteristics and weighting 3](#_Toc116039420)

[Additional modeled strategies 3](#_Toc116039421)

[Overview of the CEPAC-P model 4](#_Toc116039422)

[Model initiation and perinatal characteristics 4](#_Toc116039423)

[Postnatal characteristics 4](#_Toc116039424)

[Untreated HIV infection 4](#_Toc116039425)

[Pediatric HIV diagnosis 4](#_Toc116039426)

[Treated HIV infection 4](#_Toc116039427)

[LTFU and return to care 4](#_Toc116039428)

[Vertical HIV transmission risks 5](#_Toc116039429)

[Infant HIV prophylaxis module 5](#_Toc116039430)

[HIV-related care costs 5](#_Toc116039431)

[Oral infant prophylaxis efficacy 5](#_Toc116039432)

[BNAb efficacy and duration 6](#_Toc116039433)

[**Table A1. Literature review on the efficacy of VRC01 against infant transmitted/founder viruses** 6](#_Toc116039434)

[BNAb cost estimates 6](#_Toc116039435)

[**Table A2. Itemized costs included in modeled bNAb cost/dose** 7](#_Toc116039436)

[Determining appropriate cost-effectiveness thresholds 7](#_Toc116039437)

[**Table A3. Cost-effectiveness thresholds for LMICs proposed in published literature** 7](#_Toc116039438)

[**Figure A1. CEPAC-P infant postnatal HIV prophylaxis module flowchart** 8](#_Toc116039439)

[**Figure A2. Cost-effectiveness frontier of hybrid bNAb administration strategies** 9](#_Toc116039440)

[**Figure A3. The influence of bNAb efficacy for non-high risk infants on the ICER value of the HIVE-Extended strategy (high-risk bNAb efficacy = 60%)** 10](#_Toc116039441)

[**Table A4. Consolidated Health Economic Evaluation Reporting Standards (CHEERS) checklist** 12](#_Toc116039442)

[**Table A5. Extended model input parameters** 14](#_Toc116039443)

[**Table A6. Comparison of CEPAC-P infant HIV incidence projections with other nationally representative published estimates.** 19](#_Toc116039444)

[**Table A7. Clinical and economic outcomes of bNAb administration program, by infant risk status^†^** 20](#_Toc116039445)

[**Table A8. Scenario analysis: bNAb does not reduce intrapartum transmission (base case: 60% reduction)** 21](#_Toc116039446)

[**Table A9. Scenario analysis: intrapartum transmissions accounts for 67% of all perinatal transmissions (base case: 33%)** 22](#_Toc116039447)

[**Table A10. Scenario analysis: proportion of high-risk infants recognized as being high-risk (base case: 100%)^†^** 23](#_Toc116039448)

[**Table A11. One-way sensitivity analysis: bNAb efficacy against intrapartum and postpartum transmission** 24](#_Toc116039449)

[**Table A12. One-way sensitivity analysis: bNAb cost** 25](#_Toc116039450)

[**Table A13. One-way sensitivity analysis: bNAb effect duration^†^** 26](#_Toc116039451)

[**Table A14. One-way sensitivity analysis: bNAb toxicity** 27](#_Toc116039452)

[**Table A15. One-way sensitivity analysis: bNAb uptake** 28](#_Toc116039453)

[**Table A16. One-way sensitivity analysis: proportion of mothers on antiretroviral therapy during pregnancy** 29](#_Toc116039454)

[**Table A17. One-way sensitivity analysis: proportion of mothers with viral load <1,000 copies/mL at delivery** 30](#_Toc116039455)

[**Table A18. One-way sensitivity analysis: perinatal vertical transmission risk** 31](#_Toc116039456)

[**Table A19. One-way sensitivity analysis: breastfeeding duration** 32](#_Toc116039457)

[**Table A20. One-way sensitivity analysis: postpartum vertical transmission risk** 33](#_Toc116039458)

[**Table A21. One-way sensitivity analysis: postpartum maternal retention in care** 34](#_Toc116039459)

[**Table A22. One-way sensitivity analysis: postpartum maternal virologic suppression** 35](#_Toc116039460)

[**Table A23. One-way sensitivity analysis: antiretroviral therapy treatment cost** 36](#_Toc116039461)

[**Table A24. One-way sensitivity analysis: infant oral prophylaxis adherence** 37](#_Toc116039462)

[**Table A25. One-way sensitivity analysis: infant oral prophylaxis cost** 38](#_Toc116039463)

[**Table A26. One-way sensitivity analysis: infant oral prophylaxis efficacy** 39](#_Toc116039464)

[**Table A27. One-way sensitivity analysis: infant oral prophylaxis major toxicity leading to discontinuation** 40](#_Toc116039465)

[**Table A28. One-way sensitivity analysis: birth early infant diagnosis uptake** 41](#_Toc116039466)

[**Table A29. One-way sensitivity analysis: six to eight week early infant diagnosis uptake** 42](#_Toc116039467)

[**Table A30. One-way sensitivity analysis: six/nine month early infant diagnosis uptake** 43](#_Toc116039468)

[**Table A31. One-way sensitivity analysis: 18 month early infant diagnosis uptake** 44](#_Toc116039469)

[**Table A32. One-way sensitivity analysis: overall early infant diagnosis uptake** 45](#_Toc116039470)

[**Table A33. One-way sensitivity analysis: early infant diagnosis result return rate, return time, & linkage to care** 46](#_Toc116039471)

[**Table A34. One-way sensitivity analysis: bNAb reduction in nucleic acid amplification test sensitivity** 47](#_Toc116039472)

[**Table A35. One-way sensitivity analysis: bNAb reduction in antibody test specificity (stop bNAb if positive)** 49](#_Toc116039473)

[**Table A36. One-way sensitivity analysis: bNAb reduction in antibody test specificity (continue bNAb if positive)** 51](#_Toc116039474)

[**Table A37. One-way sensitivity analysis: 1^st^-line pediatric antiretroviral therapy efficacy** 53](#_Toc116039475)

[**Table A38. Minimum bNAb efficacy required for cost-effectiveness (50% GDP per capita) of a HIVE-Extended strategy for a variety of product characteristics across all 3 settings.** 54](#_Toc116039476)

[**References** 55](#_Toc116039477)

# **Appendix Methods**

This appendix provides supplemental details regarding modeling methodology, input specifications, and derivation of key parameters. Additional information about the Cost-Effectiveness of Preventing AIDS Complications–Pediatric (CEPAC-P) model is available in previously published works [1–10]. For further details regarding the mathematical formulas used in the model, model flowcharts, and opportunities for collaboration, we direct readers to the CEPAC website: <https://mpec.massgeneral.org/cepac-model/>.

## Cohort characteristics and weighting

Using the CEPAC-P model, we simulated two sub-cohorts of infants (Figure 1) who were known to be HIV-exposed at birth from birth until death. One sub-cohort was comprised entirely of infants who were known to be HIV-exposed and who met World Health Organization (WHO) criteria as being “high-risk for HIV” by one of the following criteria [11]:

- mother was not on antiretroviral therapy (ART) in pregnancy,
- mother was on ART in pregnancy but had an HIV viral load (VL) >1,000 copies/mL near delivery, or
- mother was on ART for less than four weeks prior to delivery (we assumed all these mothers had an HIV VL >1,000 copies/mL near delivery).

In the base-case, we assumed that all infants who fulfilled WHO high-risk criteria were recognized as being high-risk, and we varied this assumption in sensitivity analyses. All high-risk infants were eligible for WHO-recommended dual oral infant prophylaxis with twice daily zidovudine (ZDV) and once daily nevirapine (NVP) for 12 weeks. The second sub-cohort was comprised of “non-high-risk” infants (i.e., those born to mothers who did not meet one of the above WHO criteria). These infants were all eligible for WHO-recommended oral infant prophylaxis with once daily NVP for six weeks.

We weighted outcomes from the high-risk and non-high risk sub-cohorts to generate average outcomes for the entire population of all known HIV-exposed infants. The weights for each sub-cohort were calculated as follows:


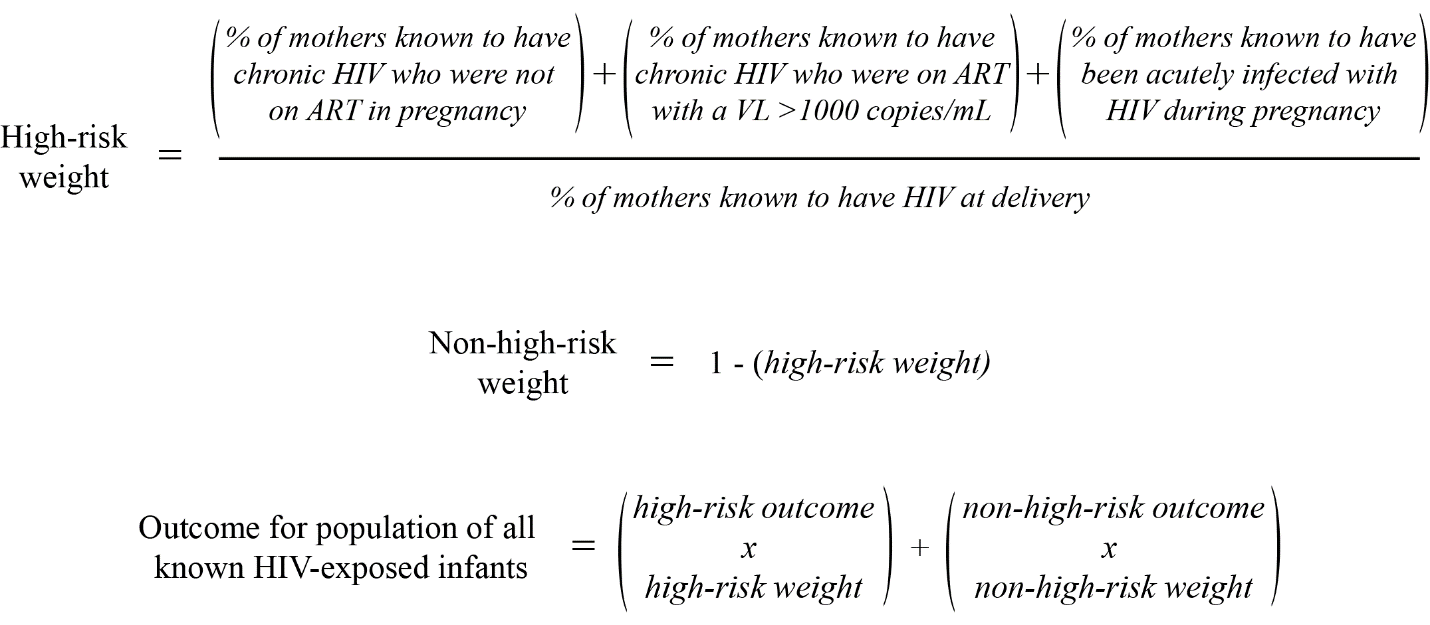


Using published data regarding country-specific HIV prevalence, HIV incidence, knowledge of HIV status, ART uptake in pregnancy, and virologic suppression to <1,000 c/mL at delivery (Table A5), the proportion of all known HIV-exposed infants meeting high-risk criteria in each country was 44% in Côte d’Ivoire, 19% in South Africa, and 24% in Zimbabwe.

We used country-specific data on the number of live births, maternal HIV prevalence, probability of incident HIV infection during pregnancy, and probability of known HIV status to estimate the number of infants known to be HIV-exposed at birth in each country: 35,000 in Cote d’Ivoire, 368,000 in South Africa, and 50,000 in Zimbabwe. We then multiplied the projected cumulative HIV incidence among known exposed infants projected in CEPAC-P by the size of the known HIV-exposed birth cohort to calculate the number of pediatric HIV infections experienced and averted by each modeled strategy.

## Additional modeled strategies

To examine the impact of a hypothetical bNAb program focused on different target populations, we modeled strategies in which bNAbs were offered only to the sub-cohort of known high-risk HIV-exposed infants (*HR-HIVE* strategies) and strategies in which bNAbs were offered to both sub-cohorts of known high-risk and non-high-risk HIV-exposed infants (*HIVE* strategies). All known HIV-exposed children were eligible for standard-of-care (*SOC*) oral infant prophylaxis in all modeled strategies.

In the main analysis, the same dosing frequency (i.e., zero doses, one dose, two doses, or extended dosing throughout breastfeeding) was used for all cohorts eligible for bNAbs. In extended analyses, we separately modeled “hybrid” strategies in which infants could receive different dosing frequencies based on risk status. For example, a strategy in which high-risk HIV-exposed infants were eligible to receive extended dosing while non-high-risk HIV-exposed infants were eligible to receive one dose was considered a hybrid strategy. All hybrid strategies were dominated by non-hybrid strategies and are thus not presented in the main analysis (Figure A2).

## Overview of the CEPAC-P model

### *Model initiation and perinatal characteristics*

The CEPAC-P model is a Monte Carlo microsimulation model of HIV infection, diagnosis, and disease progression that runs on a monthly time cycle. The model simulates individuals from birth through death (with a transition to adult model inputs at age 13) and then aggregates clinical and cost outcomes for the entire cohort. Infants start the model at birth, at which time they are assigned a set of characteristics based on their mother, including HIV status (chronically infected before pregnancy, acutely infected during pregnancy, or not living with HIV). Among women with HIV, the user specifies the probability that the mother’s HIV status is known, and if known, the probability of receiving ART during pregnancy. Among women receiving ART, the user also specifies the distribution of maternal HIV viral load near delivery into three categories: <50 c/mL, 50-1,000 c/mL, and >1,000 c/mL. Use of ART and viral load near delivery influence the perinatal HIV transmission risks applied to each infant (see “Vertical HIV transmission risks” below).

### *Postnatal characteristics*

Model users specify what proportion of the infant population is breastfed for any duration, and the mean and standard deviation of the duration of breastfeeding through a maximum of 36 months. When children stop breastfeeding, they are assumed to no longer be at risk for acquiring HIV for the remainder of the simulation. Users then specify what proportion of breastfeeding mothers are off ART (inclusive of mothers who are lost to follow up [LTFU]), on ART with VL <50 c/mL, on ART with VL 50-1,000 c/mL, and on ART with VL >1,000 c/mL in each month postpartum. Each of these categories confers a unique risk of postnatal HIV transmission, which is then applied to the simulated child in each month.

### *Untreated HIV infection*

If a child acquires HIV infection, he or she draws from distributions of HIV VL and CD4 cells; the model uses CD4 percentage (CD4%) for children under five years old and absolute CD4 count thereafter. Current age and CD4%/count in each month determine the risks of disease progression, including development of acute opportunistic infections (OIs) and death. Without effective ART, CD4%/count declines monthly. The model tracks true CD4%/count and HIV VL, although clinical decisions are made based on observed information, such as symptomatic illness or observed CD4%/count or viral loads (measured according to user-specified laboratory monitoring strategies).

### *Pediatric HIV diagnosis*

All known HIV-exposed children encounter multiple opportunities for early infant diagnosis (EID) testing in the CEPAC-P model at testing intervals consistent with country-level and WHO guidelines: at birth, two months, nine months, and 18 months in Côte d’Ivoire and Zimbabwe, or at birth, two months, six months, and 18 months in South Africa [11–13]. EID testing is modeled to use nucleic acid amplification testing (NAAT) before seroreversion (approximately 9 months of age) [14] and antibody testing after seroreversion. In the CEPAC-P model, infants experience delays in result return consistent with laboratory-based NAAT and point-of-care antibody testing programs [15–17]. Those who test positive undergo confirmatory testing with a second NAAT [10], and then face a probability of linking to care and initiating ART. For HIV-positive infants who miss testing opportunities prior to 18 months or who become infected after the last scheduled EID test, HIV diagnosis and linkage to care can be achieved at a subsequent EID visit, if available, or following an OI.

### *Treated HIV infection*

All children with HIV who have recognized HIV infection initiate first-line ART, consistent with WHO guidelines [18]. For each line of ART, we specify “ART efficacy,” defined as the probability of suppressing HIV VL to <1,000 copies/mL (c/mL) by 12 months. Children with suppressed VL experience CD4% or CD4 count gains each month. Individuals who initially achieve virologic suppression by 12 months then face a subsequent monthly risk of virologic failure (“late failure”). Following virologic failure, HIV VL gradually rises to a “set point” that is determined as a function of HIV VL at initial infection. After virologic failure, there is a 12-month delay until CD4% or CD4 count begins to decline at pre-ART rates, leading to increased monthly risks of OIs and death unless virologic suppression is achieved again. If children are observed to experience virologic failure, they receive a one-time probability of resuppressing on the same line of ART after enhanced adherence counseling [19]. If a repeat HIV viral load performed six months later redemonstrates an HIV VL >1,000 c/mL, then the individual is switched to the next line of ART. We also incorporate a reduction in mortality and OI risks for children on ART, independent of CD4 level and HIV RNA suppression, as observed in adults [1,20].

### *LTFU and return to care*

When simulated individuals with HIV are LTFU, they stop attending clinic visits and discontinue ART and co-trimoxazole prophylaxis. If ART is discontinued, the HIV viral load increases back to the starting setpoint and the CD4 count declines rapidly [21]. After hitting the viral load setpoint, simulated patients experience HIV natural history rates of CD4 decline, OIs, and AIDS-related death (Table A5). If children with HIV who are LTFU experience a WHO Stage 3 or 4 OI, they return to HIV-related care in the subsequent month. Individuals who are LTFU also have a “background” probability of returning to care, independent from OI-driven return to care, after being out of care for a minimum of six months [22]. When individuals return to care, they reinitiate ART. Patients who were previously suppressed on ART return to the same line of ART they were taking prior to being LTFU. If they were failing ART prior to being LTFU, but not yet recognized as failing, they return to the line of ART they were taking previously. If they were recognized as failing ART at the time of becoming LTFU but had not yet transitioned to the next ART line, they initiate the next ART line when returning to care.

### *Vertical HIV transmission risks*

All HIV-exposed infants experience a one-time risk of intrauterine (IU)/intrapartum (IP) HIV infection, followed by monthly risks of postnatal HIV infection while breastfeeding. All HIV transmission risks are stratified by recency of maternal HIV infection (acute or chronic), maternal ART use (yes or no), and maternal HIV viral load (<50, 50-1,000, or >1,000 c/mL). For infants born to mothers who received ART in pregnancy, IU/IP transmission risks include the impact of oral infant prophylaxis on reducing intrapartum transmission. For infants born to mothers who are known to be living with HIV, but who were not on ART in pregnancy, the impact of oral infant prophylaxis on reducing intrapartum transmission is not captured by the IU/IP transmission risk. However, these infants do receive a separate probability of receiving oral infant prophylaxis and subsequent reduction in intrapartum transmission risk. Vertical transmission risks were informed by two meta-analyses regarding the peri- and post-natal HIV transmission risks by ART use [23] and viral load [24]. Country-specific rates of maternal ART use and virologic suppression were informed by published literature (Table A5) and varied by month throughout breastfeeding.

### *Infant HIV prophylaxis module*

The infant HIV prophylaxis module (Figure A1) is embedded within the CEPAC-P model and allows the user to simulate up to four concurrent and independent lines of HIV prophylaxis. The influence of infant HIV prophylaxis on reducing IP transmission is reflected as a one-time multiplier on the IP component of the overall IU/IP transmission risks at birth. For all months after birth, children who are breastfeeding, have not had a prior positive HIV test, and who do not have an active dose of prophylaxis from a prior month face optional user-specified prophylaxis eligibility criteria based on their age, maternal characteristics, and EID test results. If the child meets eligibility criteria for that line of prophylaxis, they then face a probability of access and adherence to prophylaxis each month. If the child receives a dose of prophylaxis, an “efficacy multiplier” is applied to reduce postnatal transmission for a user-specified number of months during which the prophylaxis dose is active. Irrespective of efficacy, if a child receives prophylaxis in a given month, he or she also experiences a probability of mild and/or severe drug toxicity in the month the dose was administered. Every toxicity event also carries a toxicity cost; when a severe toxicity is encountered, it also carries a probability of death in that month and triggers the prophylaxis regimen to be permanently stopped.

### *HIV-related care costs*

All HIV-related costs were modeled from the healthcare system perspective [25]. While in HIV care, simulated individuals accumulate CD4-stratified routine care costs reflecting the costs of clinic infrastructure, provider time, and laboratory monitoring other than CD4 and HIV viral load tests (Table A5) [26–29]. During periods of LTFU, we assume that individuals receive 20% of monthly routine care costs. Routine care costs for Zimbabwe were inclusive of the cost of treating OIs. In Côte d’Ivoire and South Africa, OI-related costs and routine HIV care costs are modeled separately. The costs of OI-related care for children less than 5 years old were derived from previously published studies of healthcare costs for HIV-infected children from South Africa and Côte d’Ivoire [26,30,31]. Costs of OI-related care for individuals older than five years old were calculated by multiplying resource use (e.g. outpatient visits, inpatient days, laboratory testing, and medication use) from the Cape Town AIDS Cohort (South Africa) and ANRS 059 cohort (Côte d’Ivoire) by country-specific unit costs [29,31,32].

While in care, people with HIV undergo routine lab monitoring with HIV viral load and CD4 tests, consistent with WHO guidelines [11]. Lab monitoring and infant HIV diagnostic costs were informed by the Global Fund’s price list and procurement tools [33]. Antiretroviral (ARV) prophylaxis and ART regimen costs were also informed by the Global Fund’s price list [34], but then adjusted for weight, age, and ARV formulation [35].

## Oral infant prophylaxis efficacy

All known HIV-exposed children in this analysis experienced a probability of receiving WHO-recommended standard of care oral infant prophylaxis, including those in the bNAb strategies. Consistent with WHO guidelines, modeled non-high-risk infants were eligible for dual oral infant prophylaxis with NVP alone for six weeks, while high-risk infants were eligible for NVP + ZDV for 12 weeks. With conflicting data on the efficacy of one vs. multi-drug infant prophylaxis regimens, we modeled the same relative efficacy of both regimens [36].

Modeled perinatal transmission risks among women on ART in pregnancy were largely derived from studies in which infants received short courses of antiretroviral prophylaxis after birth. Therefore, we did not model additional reductions in perinatal transmission among infants born to mothers on ART in pregnancy. However, among infants born to women who did not receive ART in pregnancy, we modeled a 75% relative reduction in the intrapartum component of perinatal transmission with oral infant prophylaxis based on data from the PEPI study [37]. In the PEPI study, the cumulative HIV incidence at six weeks among breastfeeding infants who received a single NVP dose plus one week of ZDV was 5.10% after excluding infants identified as having HIV infection at birth. Cumulative HIV incidence by 6 weeks was much lower among infants who received control plus extended (through 14 weeks) dosing of nevirapine (1.67%, relative risk [RR]: 0.33) or control plus extended dual prophylaxis (NVP + ZDV) (1.58%, relative risk [RR]: 0.31). These relative risks of 0.31-0.33 roughly translate to an oral antiretroviral prophylaxis efficacy of 67-69%, which likely would have been even higher had the control arm in the study not received single-dose nevirapine and zidovudine for 1 week. Therefore, we assumed 75% efficacy of standard of care oral antiretroviral prophylaxis (vs. no infant oral antiretroviral prophylaxis) in the base case. While transmission by six weeks may include some early postnatal transmission, we applied this risk reduction to the intrapartum component of projected vertical transmission risks in the model, to be conservative with respect to the relative incremental benefits of bNAbs. We also modeled a 71% decrease in postnatal transmission risk with oral infant prophylaxis based on an analysis of pooled individual data from five randomized trials of infant NVP prophylaxis demonstrating an adjusted hazard ratio (in time-varying analyses) of 0.29 (95% CI: 0.20-0.42) for vertical transmission while NVP was administered [38].

## BNAb efficacy and duration

Since there have been no published studies of bNAb efficacy for human infant HIV prophylaxis, the base case efficacy estimate of 60% was estimated based on data from human adult, non-human primate, and in vitro studies. In the Antibody Mediated Prevention (AMP) trials, intravenous infusions of VRC01 failed to prevent sexual acquisition of HIV infection among overall populations of cisgender men and transgender adults in the Americas and Europe, and in women in sub-Saharan Africa [39]. However, HIV incidence with VRC01-sensitive isolates with an IC_80_ <1 µg/mL was 75.4% lower among individuals who received VRC01 compared to individuals who received placebo. A literature review also revealed 77-100% susceptibility of infant transmitted/founder viruses to VRC01 in recent studies (see table below).

### **Table A1. Literature review on the efficacy of VRC01 against infant transmitted/founder viruses**

| **Citation** | **# of infant T/F viruses** | **# of infant T/F viruses resistant to VRC01** | **Efficacy of VRC01 (%)** | **IC_50_ cutoff, (µg/mL)** |
| --- | --- | --- | --- | --- |
| Fouda GG et al., 2013 [40] | 7 | 1 | 86 | 25 |
| Nakamura KJ et al., 2013 [41] | 23 | 5 | 78 | 10 |
| Russell ES et al., 2013 [42] | 6 | 1 | 83 | 25 |
| Mabuka J, Goo L, Omenda MM, Nduati R, Overbaugh J, 2013 [43] | 22 | 5 | 77 | 1 |
| Kumar A et al., 2018 [44] | 21 | 0 | 100 | 5 |
| Martinez DR et al., 2020 [45] | 6 | 1 | 83 | 50 |
| **TOTAL** | 85 | 13 | 85 | ... |

T, transmitted; F, founder; IC_50_, concentration of drug required for 50% inhibition.

Other bNAbs under investigation, including VRC07-523L, PGT121.414LS, 1-18, 3BNC117-LS, and 10-1074-LS are more potent than VRC01 both alone and in combination [46,47]. In multiclade panels, 84-92% of isolates were susceptible to one or more of these bNAbs [47]. Using the lower bound of susceptibility to those bNAbs, if 84% of circulating viruses were susceptible to a bNAb with 75% efficacy as in the AMP study, estimated overall population efficacy would be 84% x 75% = 63%. Therefore, we assumed 60% overall efficacy per dose of bNAbs in our base case analysis.

While the AMP studies investigated bNAb efficacy when delivered as pre-exposure prophylaxis, non-human primate studies have also observed efficacy of bNAbs as post-exposure prophylaxis if given within 24-30 hours of exposure [48,49]. Based on these data, we assumed that a bNAb given at birth would reduce the risk of IP HIV transmission as post-exposure prophylaxis, in addition to acting as pre-exposure prophylaxis for postnatal transmission.

Pharmacokinetic data from HIV-exposed infants suggest that with an 80mg dose of VRC07-523LS delivered subcutaneously at birth, concentrations of the bNAb would remain sufficiently high to achieve protective efficacy through at least 12 weeks of life [46]. In our base case, we assumed a three-month duration of effect following each bNAb dose, and we varied this assumption in sensitivity analyses.

## BNAb cost estimates

As there are no bNAb products currently approved for clinical use, their cost has not been established. Therefore, we estimated the cost of delivering bNAbs in resource-limited settings based on existing literature regarding monoclonal antibody production costs [50], implementation of a new vaccine that has cold chain costs [51,52], and facility/overhead costs associated with postnatal HIV-related care [53].

### **Table A2. Itemized costs included in modeled bNAb cost/dose**

| **Cost components of bNAb implementation at scale** | **Cost** |
| --- | --- |
| bNAb production costs per 100mg dose [50] | $2.00-20.00 |
| Supply and delivery costs per dose, including: training, social mobilization, hand hygiene, personal protective equipment, waste management, transport, and cold-chain costs [51,52] | $0.34-1.66 |
| Service delivery personnel cost [52] | $1.00-2.14 |
| Facility overhead and capital costs [53] | $2.44-5.37 |
| **Total estimated cost** | **$5.78-29.17** |

Total estimated cost of bNAb implementation at scale ranged from $5.78-29.17 for a single bNAb product. If multiple bNAbs were used in combination, production costs could potentially double, with total estimated costs increasing to $7.78-$49.17. Supply costs, facility overhead, and capital costs would all likely be higher at lower scale early in the bNAb implementation process. Therefore, to account for the upper bound of estimated costs and to be conservative with respect to the potential cost-effectiveness of bNAb strategies, we used a cost of $60/dose of bNAbs in the base case.

## Determining appropriate cost-effectiveness thresholds

While the WHO-CHOICE 100% GDP per-capita-based cost-effectiveness threshold (CET) has been widely cited, there is growing concern that spending to this threshold may not offer good value, particularly in resource-limited settings [54–57]. Due to budget constraints, investing in interventions at this GDP-based CET may result in substantial opportunity costs (e.g., forgone health benefits) relative to other investments in health care that offer better value [54–56].

Several alternative approaches to determining an appropriate CET for resource-limited settings have been proposed. In recent years, two teams of investigators at the Centre for Health Economics at the University of York used novel approaches to estimate CETs. Woods et al. applied international income elasticities to estimates of forgone health benefit from the National Health Service in the UK in order to generate country-specific ranges of alternative CETs [54]. Ochalek et al. examined the relationship between levels of healthcare expenditure and mortality using data from the World Bank and Global Burden of Disease Study to estimate CETs [58]. More recently, Mark Jit examined the projected CET at which low income countries introduced the WHO-recommended human papilloma virus vaccine, finding that the optimal threshold was an incremental cost-effectiveness ratio of 30-40% GDP per capita [59]. In two other studies (Edoka et al. and Meyer-Rath et al.) [56,60], investigators in South Africa evaluated health opportunity costs reflecting revealed willingness to pay to estimate an appropriate CET for that setting.

All of the CETs proposed by these alternative approaches were consistently less than the WHO-CHOICE recommended 100% GDP per capita in resource-limited settings. However, there was a wide range in recommended CETs between these different approaches:

### **Table A3. Cost-effectiveness thresholds for LMICs proposed in published literature**

| **Source** | **Cost-effectiveness threshold (in % GDP per capita)** | | |
| --- | --- | --- | --- |
|  | **Côte d’Ivoire** | **South Africa** | **Zimbabwe** |
| Woods B, Revill P, Sculpher M, Claxton K, 2016 [54] | 4-52% | 17-69% | 1-32% |
| Ochalek JM, Lomas J, Claxton KP, 2018 [58] | 15-19% | 43-58% | 22-30% |
| Jit M, 2021 [59] | 30-40% | 30-40% | 30-40% |
| Edoka EP, Stacey NK, 2020 [60] | ... | 53% | ... |
| Meyer-Rath G, Rensburg C, Larson B, Jamieson L, Rosen S, 2017 [56] | ... | 10-17% | ... |

Given the heterogeneity in appropriate CETs recommended by these approaches, we decided to evaluate the cost-effectiveness of bNAb infant HIV prophylaxis at two different thresholds, a “conservative” threshold of 20% GDP per capita per year of life saved, and a “relaxed” threshold of 50% GDP per capita per year of life saved.

# **Figure A1. CEPAC-P infant postnatal HIV prophylaxis module flowchart**


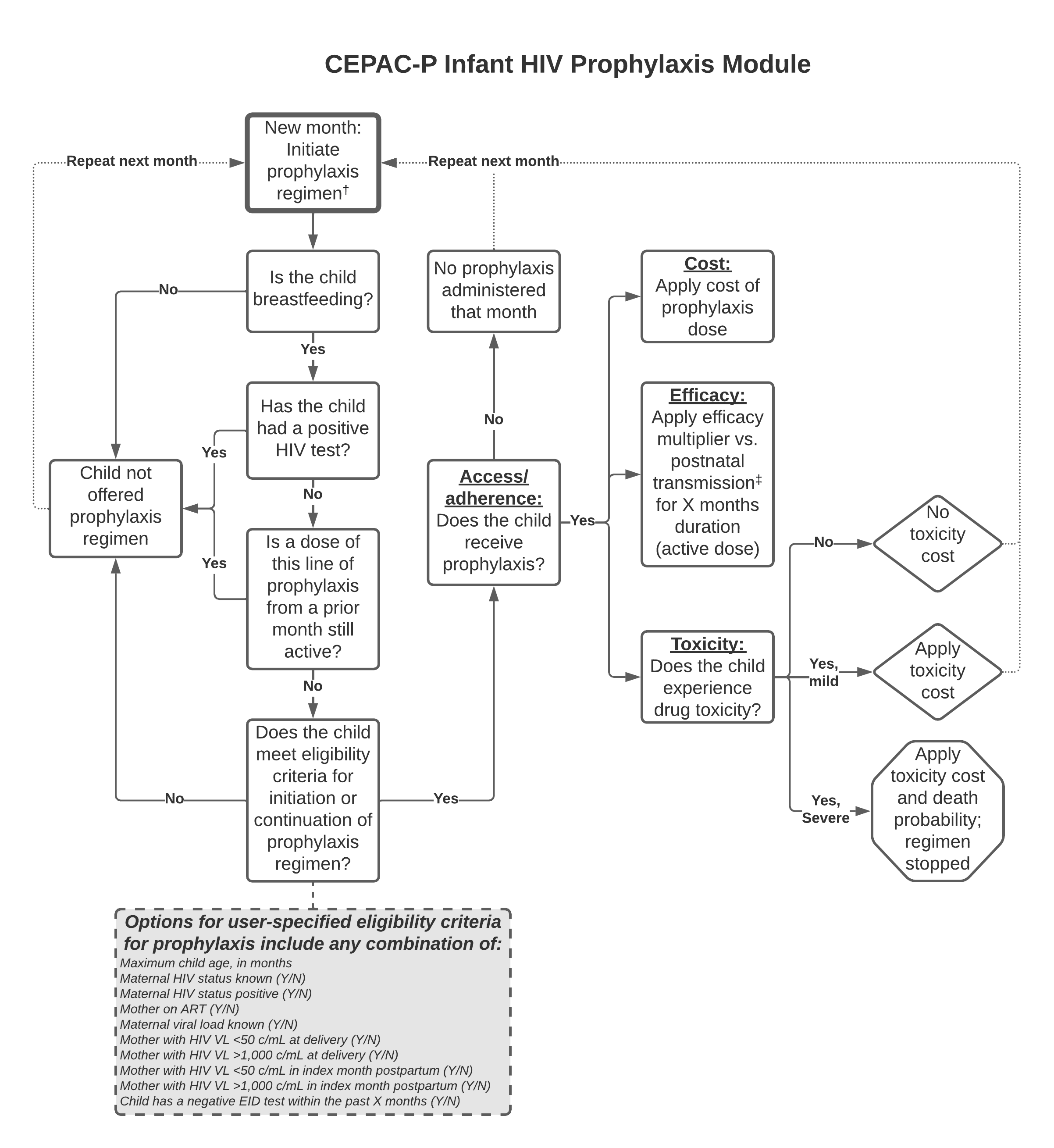


CEPAC-P, Cost-Effectiveness of Preventing AIDS Complications–Pediatrics Model; Y, yes; N, no; ART, antiretroviral therapy; VL, viral load; EID, early infant diagnosis.

^†^ This process is repeated separately for each line of prophylaxis each month.

^‡^ Efficacy of prophylaxis against intrapartum transmission is applied directly as a one-time multiplier against the intrapartum component of the intrauterine/intrapartum transmission risk.

**Figure A2. Cost-effectiveness frontier of hybrid bNAb administration strategies**


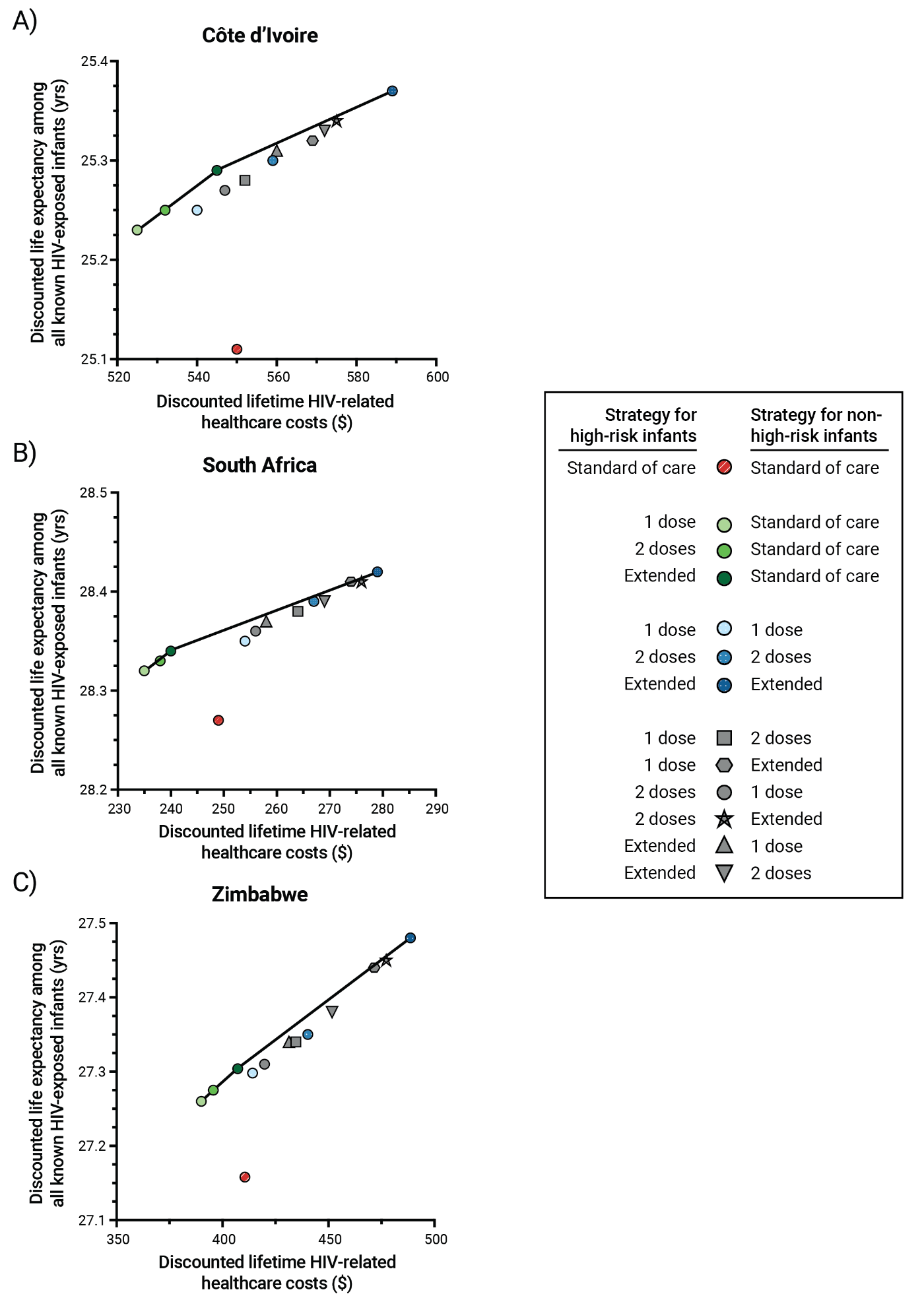


Costs are presented in 2019 USD. Discounted values are discounted at rate of 3% per year.

bNAb: broadly neutralizing antibody; yrs, years.

**Figure A3. The influence of bNAb efficacy for non-high risk infants on the ICER value of the *HIVE-Extended* strategy (high-risk bNAb efficacy = 60%)**

**
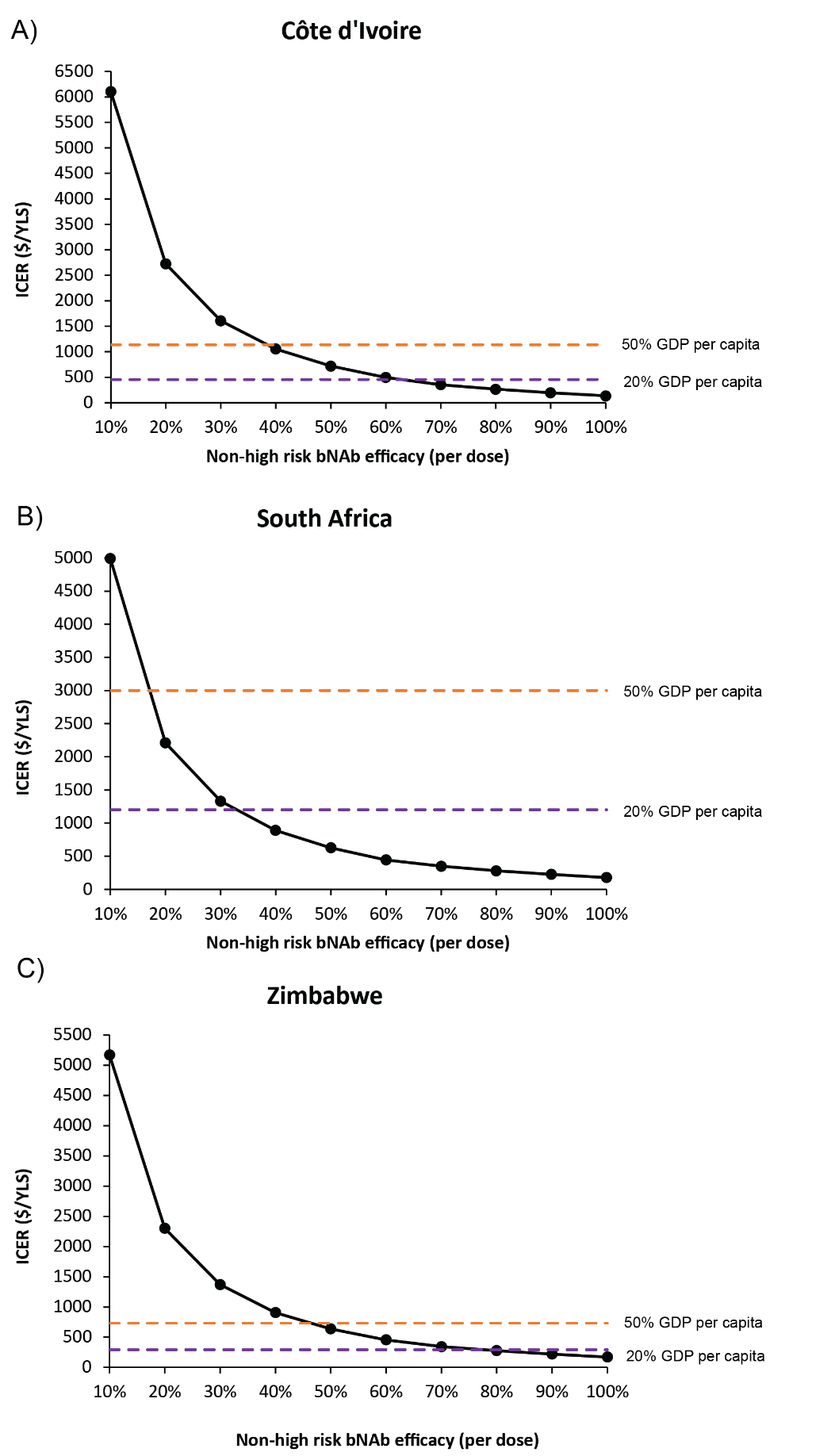
**

Costs are presented in 2019 USD.

bNAb: broadly neutralizing antibody; ICER: incremental cost-effectiveness ratio; YLS: year of life saved.

# **Table A4. Consolidated Health Economic Evaluation Reporting Standards (CHEERS) checklist**

| **Section/item** | **Item No** | **Recommendation** | **Reported on page No/ line No** |
| --- | --- | --- | --- |
| **Title and abstract** |  |  |  |
| Title | 1 | Identify the study as an economic evaluation or use more specific terms such as “cost effectiveness analysis”, and describe the interventions compared. | Title |
| Abstract | 2 | Provide a structured summary of objectives, perspective, setting, methods (including study design and inputs), results (including base case and uncertainty analyses), and conclusions. | Abstract |
| **Introduction** |  |  |  |
| Background and objectives | 3 | Provide an explicit statement of the broader context for the study | Introduction, paragraphs 1-3 |
|  |  | Present the study question and its relevance for health policy or practice decisions | Introduction, paragraph 4 |
| **Methods** |  |  |  |
| Target population and subgroups | 4 | Describe characteristics of the base case population and subgroups analysed, including why they were chosen. | Methods, paragraphs 1, 3, & 4 |
| Setting and location | 5 | State relevant aspects of the system(s) in which the decision(s) need(s) to be made. | Methods, paragraph 1 & 6 |
| Study perspective | 6 | Describe the perspective of the study and relate this to the costs being evaluated. | Methods, paragraph 5 |
| Comparators | 7 | Describe the interventions or strategies being compared and state why they were chosen. | Methods, paragraph 4 |
| Time horizon | 8 | State the time horizon(s) over which costs and consequences are being evaluated and say why appropriate. | Methods, paragraph 5 |
| Discount rate | 9 | Report the choice of discount rate(s) used for costs and outcomes and say why appropriate. | Methods, paragraph 5 |
| Choice of health outcomes | 10 | Describe what outcomes were used as the measure(s) of benefit in the evaluation and their relevance for the type of analysis performed. | Methods, paragraph 5 |
| Measurement of effectiveness | 11a | Single study-based estimates: Describe fully the design features of the single effectiveness study and why the single study was a sufficient source of clinical effectiveness data. | N/A |
|  | 11b | Synthesis-based estimates: Describe fully the methods used for identification of included studies and synthesis of clinical effectiveness data. | Methods, paragraphs 6 & 7; Appendix Methods, page 6 |
| Measurement and valuation of preference based outcomes | 12 | If applicable, describe the population and methods used to elicit preferences for outcomes. | N/A |
| Estimating resources and costs | 13a | Single study-based economic evaluation: Describe approaches used to estimate resource use associated with the alternative interventions. Describe primary or secondary research methods for valuing each resource item in terms of its unit cost. Describe any adjustments made to approximate to opportunity costs. | N/A |
|  | 13b | Model-based economic evaluation: Describe approaches and data sources used to estimate resource use associated with model health states. Describe primary or secondary research methods for valuing each resource item in terms of its unit cost. Describe any adjustments made to approximate to opportunity costs. | Methods, paragraphs 6 & 7; Appendix Methods, page 7; Table A3; Table A5 |
| Currency, price date, and conversion | 14 | Report the dates of the estimated resource quantities and unit costs. Describe methods for adjusting estimated unit costs to the year of reported costs if necessary. Describe methods for converting costs into a common currency base and the exchange rate. | Methods, paragraph 5 |
| Choice of model | 15 | Describe and give reasons for the specific type of decision-analytical model used. Providing a figure to show model structure is strongly recommended. | Methods, paragraph 1; Figure A1 |
| Assumptions | 16 | Describe all structural or other assumptions underpinning the decision-analytical model. | Methods, paragraphs 1 & 2; Figure A1 |
| Analytical methods | 17 | Describe all analytical methods supporting the evaluation. This could include methods for dealing with skewed, missing, or censored data; extrapolation methods; methods for pooling data; approaches to validate or make adjustments (such as half cycle corrections) to a model; and methods for handling population heterogeneity and uncertainty. | Methods, paragraphs 1 & 8; Appendix Methods |
| **Results** |  |  |  |
| Study parameters | 18 | Report the values, ranges, references, and, if used, probability distributions for all parameters. Report reasons or sources for distributions used to represent uncertainty where appropriate. Providing a table to show the input values is strongly recommended. | Table 1; Table A5 |
| Incremental costs and outcomes | 19 | For each intervention, report mean values for the main categories of estimated costs and outcomes of interest, as well as mean differences between the comparator groups. If applicable, report incremental cost-effectiveness ratios. | Results, paragraph 3; Table 2 |
| Characterising uncertainty | 20a | Single study-based economic evaluation: Describe the effects of sampling uncertainty for the estimated incremental cost and incremental effectiveness parameters, together with the impact of methodological assumptions (such as discount rate, study perspective). | N/A |
|  | 20b | Model-based economic evaluation: Describe the effects on the results of uncertainty for all input parameters, and uncertainty related to the structure of the model and assumptions. | Results, paragraphs 4-5; Figure 3; Tables A8-A37 |
| Characterising heterogeneity | 21 | If applicable, report differences in costs, outcomes, or cost-effectiveness that can be explained by variations between subgroups of patients with different baseline characteristics or other observed variability in effects that are not reducible by more information. | Results, paragraphs 1-5; Figures 2-3; Tables A8-A37 |
| **Discussion** |  |  |  |
| Study findings, limitations, generalizability, and current knowledge | 22 | Summarise key study findings and describe how they support the conclusions reached. Discuss limitations and the generalisability of the findings and how the findings fit with current knowledge. | Discussion |
| **Other** |  |  |  |
| Source of funding | 23 | Describe how the study was funded and the role of the funder in the identification, design, conduct, and reporting of the analysis. Describe other non-monetary sources of support. | Acknowledgements, Funding |
| Conflicts of interest | 24 | Describe any potential for conflict of interest of study contributors in accordance with journal policy. In the absence of a journal policy, we recommend authors comply with International Committee of Medical Journal Editors recommendations. | Conflicts of Interest Statement |

# **Table A5. Extended model input parameters**

| Parameter | Côte d’Ivoire | South Africa | Zimbabwe | Reference(s) |
| --- | --- | --- | --- | --- |
| Maternal characteristics |  |  |  |  |
| Probability of acute HIV infection in pregnancy, % | 1.2 | 3.3 | 1.9 | [61–66] |
| Proportion of mothers with chronic infection, % |  |  |  |  |
| Non-high-risk cohort | 100 | 100 | 100 | Assumption |
| High-risk cohort | 64 | 79 | 57 | Derived from [61–81] |
| Probability of known HIV status in pregnancy, % |  |  |  | Derived from [17,67–70,76,79,81,82] |
| Among mothers with acute infection | 56 | 55 | 70 |  |
| Among mothers with chronic infection | 92 | 99 | 98 |  |
| Probability of being on ART during pregnancy, % |  |  |  |  |
| Non-high-risk cohort | 100 | 100 | 100 | Assumption |
| High-risk cohort |  |  |  |  |
| Among those with chronic infection | 60 | 84 | 53 | Derived from [72,73,75–81] |
| Among those with acute infection | 87 | 97 | 93 | Derived from [61–77,81] |
| Viral load if on ART at delivery, % |  |  |  |  |
| Non-high-risk cohort |  |  |  | Derived from [72,73,75–81] |
| HIV RNA <50 c/mL | 76 | 76 | 76 |  |
| HIV RNA 50-1,000 c/mL | 24 | 24 | 24 |  |
| HIV RNA >1,000 c/mL | 0 | 0 | 0 |  |
| High-risk cohort |  |  |  | Assumption |
| HIV RNA >1,000 c/mL | 100 | 100 | 100 |  |
| Probability of delivering in a healthcare facility, % | 70 | 96 | 77 | [72,73,83] |
| HIV RNA <1,000 c/mL among postpartum women on ART, by month postpartum | 77-92 | 77-92 | 77-92 | [84–93] |
| Retention in HIV care, % |  |  |  | [94] |
| 6 mo. postpartum | 87 | 88 | 88 |  |
| 12 mo. postpartum | 87 | 87 | 87 |  |
| 24 mo. postpartum | 79 | 79 | 79 |  |
| Infant cohort characteristics and transmission risks |  |  |  |  |
| Total number of live births (thousands) | 888 | 1158 | 427 | [95] |
| Age, mean (SD), mo. | 0 (0) | 0 (0) | 0 (0) | Assumption |
| Proportion female, % | 52 | 52 | 52 | [96] |
| Initial CD4% at infection, mean (SD), % | 45 (10) | 45 (10) | 45 (10) | [1] |
| Estimated number of known HIV-exposed infants born per year (thousands) | 35 | 368 | 50 | Derived from [61–70,72–82,95] |
| Breastfed infants, % of known HIV-exposed infants | 97 | 66 | 94 | [72,79,97] |
| Breastfeeding duration, mean (SD) mo. | 14 (7) | 6 (6) | 13 (7) | [72,74,87,98] |
| Infants meeting WHO high-risk criteria at birth, % | 44 | 19 | 24 | Derived from [61–70,72–82] |
| Intrauterine infections as a share of all perinatal infections, % | 67 | 67 | 67 | [11] |
| Perinatal transmission, one-time % |  |  |  |  |
| Chronic maternal HIV infection in pregnancy |  |  |  |  |
| On ART, HIV RNA <50 c/mL at delivery | 0.24 | 0.24 | 0.24 | [24] |
| On ART, HIV RNA 50-1,000 c/mL at delivery | 1.45 | 1.45 | 1.45 | [24] |
| On ART, HIV RNA >1,000 c/mL at delivery | 4.14 | 4.14 | 4.14 | [24] |
| Not on ART at delivery | 19.70 | 19.70 | 19.70 | [23] |
| Acute maternal HIV infection in pregnancy |  |  |  |  |
| On ART at delivery | 8.33 | 8.33 | 8.33 | [23,24] |
| Not on ART at delivery | 18.10 | 18.10 | 18.10 | [23] |
| Postnatal transmission rate, %/mo.^†^ |  |  |  |  |
| On ART, HIV RNA <50 c/mL | 0.06 | 0.06 | 0.06 | [24] |
| On ART, HIV RNA 50-1,000 c/mL | 0.39 | 0.39 | 0.39 | [24] |
| On ART, HIV RNA >1,000 c/mL | 0.78 | 0.78 | 0.78 | [24] |
| Not on ART | 0.89 | 0.89 | 0.89 | [23] |
| Early infant diagnosis |  |  |  |  |
| Probability of uptake, % by age |  |  |  |  |
| Birth | 0 | 93 | 9 | [99,100] |
| 6-8 weeks | 69 | 64 | 54 | [17,80,87,101–105] |
| 6/9 months (country-specific timepoint) | 25 | 25 | 26 | [80,106] |
| 18 months | 22 | 22 | 22 | [107] |
| Nucleic acid amplification test characteristics |  |  |  |  |
| Sensitivity, 0 months after infection, % | 0 | 0 | 0 | Assumption |
| Sensitivity, >1 month after infection, % | 99.4 | 99.4 | 99.4 | [108] |
| Specificity, % | 99.6 | 99.6 | 99.6 | [108] |
| Probability of result return, % | 84 | 96 | 83 | [16,17,109] |
| Delay between primary test and result return, mean mo. (SD) | 2 (1) | 2 (1) | 2 (1) | [16,17] |
| Probability of linkage to care upon detection, % | 59.8 | 80.7 | 59.8 | [15,110] |
| Antibody test characteristics |  |  |  |  |
| Sensitivity, 0 months after infection, % | 0 | 0 | 0 | Assumption |
| Sensitivity, >1 month after infection, % | 100 | 100 | 100 | [111,112] |
| Specificity, % |  |  |  |  |
| Before seroconversion | 0.01 | 0.01 | 0.01 | Assumption |
| After seroconversion | 99 | 99 | 99 | [112] |
| Probability of result return, % | 99.6 | 99.5 | 99.7 | [15,16] |
| Delay between primary test and result return, mean mo. (SD) | 0 (0) | 0 (0) | 0 (0) | [16]/Assumption |
| Probability of linkage to care upon detection, % | 92.6 | 92.6 | 92.6 | [15] |
| Probability of HIV detection after presenting to care with a severe OI, % | 100 | 100 | 100 | Assumption |
| Infant HIV prophylaxis |  |  |  |  |
| Probability of receiving scheduled prophylaxis, % |  |  |  |  |
| Standard of care oral infant prophylaxis (NVP +/- ZDV) ^‡^ | 86 | 86 | 86 | [113] |
| bNAb prophylaxis (varies by age)^§^ | 31-71 | 54-96 | 62-89 | [72,73,83] |
| Efficacy of standard of care oral infant prophylaxis (NVP +/- ZDV)^‡,¶^ % |  |  |  |  |
| Against intrapartum transmission | 75 | 75 | 75 | [114] |
| Against postnatal transmission | 71 | 71 | 71 | [38] |
| Efficacy of bNAb prophylaxis against intrapartum and postnatal transmission (in addition to efficacy of standard of care), % | 60 | 60 | 60 | Assumption based on [39,47] |
| Duration of bNAb effect, mo. | 3 | 3 | 3 | [46,115] |
| Probability of infant standard of care oral prophylaxis major toxicity, one-time % | 1.5 | 1.5 | 1.5 | Assumption based on [116–118] |
| Probability of infant bNAb prophylaxis minor toxicity, one-time % | 50 | 50 | 50 | Assumption |
| Probability of infant bNAb prophylaxis major toxicity, one-time % | 0 | 0 | 0 | Assumption |
| Natural history |  |  |  |  |
| Monthly CD4% or CD4 decline in absence of ART, by age |  |  |  |  |
| <5 years (range by age and time of transmission), % | 0.5 – 4.0 | 0.5 – 4.0 | 0.5 – 4.0 | [1] |
| ≥5 years, mean (SD) (range by viral load and CD4), cells/mm^3^ | 3.0 (0.3) – 6.4 (0.3) | 3.0 (0.3) – 6.4 (0.3) | 3.0 (0.3) – 6.4 (0.3) | [1,21] |
| Monthly risk of clinical events, by age, % |  |  |  |  |
| 0-5 years (range by age and CD4%) |  |  |  | [96] |
| WHO stage 3 event (except tuberculosis) | 3.3 – 11.6 | 3.3 – 11.6 | 3.3 – 11.6 |  |
| WHO stage 4 event (except tuberculosis) | 1.4 – 6.4 | 1.4 – 6.4 | 1.4 – 6.4 |  |
| Tuberculosis (any body site) | 0.5 – 3.8 | 0.5 – 3.8 | 0.5 – 3.8 |  |
| ≥5 years (range by CD4) |  |  |  | [29,32] |
| Mild fungal infection | 0.75 – 9.42 | 1.76 – 3.14 | 1.8 – 3.1 |  |
| Mild bacterial infection | 0.93 – 2.01 | 0 | 0 |  |
| Bacterial gastroenteritis | 0.18 – 1.33 | 0 | 0 |  |
| Malaria | 0.85 – 2.97 | 0 | 0 |  |
| Visceral bacterial infection | 0.15 – 1.70 | 0.04 – 0.71 | 0.04 – 0.71 |  |
| WHO stage 3 or 4 visceral disease | 0.03 – 2.92 | 0.03 – 1.43 | 0.03 – 1.43 |  |
| WHO stage 3 or 4 mucocutaneous disease | 0.01 – 2.74 | 0.03 – 2.26 | 0.03 – 2.26 |  |
| Other WHO stage 3 or 4 disease | 0.04 – 2.08 | 0.02 – 0.73 | 0.02 – 0.73 |  |
| Other severe disease | 0.75 – 3.79 | 0.19 – 1.67 | 0.19 – 1.67 |  |
| Other mild infection | 0.72 – 4.16 | 2.39 | 2.39 |  |
| Tuberculosis (any body site) | 0.06 – 0.66 | 0.03 – 1.74 | 0.03 – 1.74 |  |
| Risk of death within 30 days of clinical event, by age, % |  |  |  |  |
| 0-5 years |  |  |  | [1,96] |
| WHO stage 3 or 4 event | 13.5 | 13.5 | 13.5 |  |
| Tuberculosis | 11.1 | 11.1 | 11.1 |  |
| ≥5 years (range by CD4) |  |  |  | [29,32] |
| Mild fungal infection | 0 | 0 | 0 |  |
| Mild bacterial infection | 0 | ... | ... |  |
| Bacterial gastroenteritis | 0 | ... | ... |  |
| Malaria | 0 – 16.7 | ... | ... |  |
| Visceral bacterial infection | 0 – 16.7 | 0 | 0 |  |
| WHO stage 3 or 4 visceral disease | 6.7 – 12.3 | 0.5 | 0.5 |  |
| WHO stage 3 or 4 mucocutaneous disease | 0 | 0 | 0 |  |
| Other WHO stage 3 or 4 disease | 6.7 – 12.3 | 0 | 0 |  |
| Other severe disease | 0 – 16.7 | 0.4 | 0.4 |  |
| Other mild infection | 0 | 2.4 | 2.4 |  |
| Tuberculosis (any body site) | 6.5 – 50.0 | 9.2 | 9.2 |  |
| Monthly risk of HIV-related mortality, by age, % (range by CD4 and OI history) |  |  |  | [1,29,32,96] |
| 0-5 months | 5.3 – 40.8 | 5.3 – 40.8 | 5.3 – 40.8 |  |
| 6-11 months | 2.2 – 16.8 | 2.2 – 16.8 | 2.2 – 16.8 |  |
| 12-23 months | 0.9 – 7.2 | 0.9 – 7.2 | 0.9 – 7.2 |  |
| 24-35 months | 0.5 – 3.6 | 0.5 – 3.6 | 0.5 – 3.6 |  |
| 36-47 months | 0.4 – 2.9 | 0.4 – 2.9 | 0.4 – 2.9 |  |
| 48-59 months | 0.1 – 1.0 | 0.1 – 1.0 | 0.1 – 1.0 |  |
| 5-13 years | 0.1 – 1.0 | 0.1 – 1.0 | 0.1 – 1.0 |  |
| ≥13 years | 0.04 – 5.4 | 0.2 – 9.5 | 0.2 – 9.5 |  |
| Monthly risk of non-HIV related mortality, % |  |  |  |  |
| HIV-exposed, uninfected children, born to mothers not on ART, by age |  |  |  | [119,120] |
| 0-2 months | 1.01 | 1.01 | 1.01 |  |
| 3-5 months | 0.41 | 0.41 | 0.41 |  |
| 6-11 months | 0.28 | 0.28 | 0.28 |  |
| 12-17 months | 0.14 | 0.14 | 0.14 |  |
| 18-23 months | 0.07 | 0.07 | 0.07 |  |
| ≥24 months | See below | See below | See below |  |
| All others, by age |  |  |  | [121,122] |
| 0-11 months (range by age and sex) | 0.50 – 0.66 | 0.24 – 0.31 | 0.30 – 0.38 |  |
| 12-23 months (range by sex) | 0.05 – 0.06 | 0.02 | 0.03 |  |
| 24-59 months (range by sex) | 0.05 – 0.06 | 0.02 | 0.03 |  |
| 5-13 years (range by age and sex) | 0.02 – 0.03 | 0.003 – 0.006 | 0.01 – 0.02 |  |
| 13-99 years | 0.02 – 2.40 | 0.004 – 1.930 | 0.01 – 1.89 |  |
| 100 years | 100 | 100 | 100 |  |
| Treatment |  |  |  |  |
| Efficacy of adult OI prophylaxis (co-trimoxazole) (range by OI type), % | 18 – 88 | 18 – 50 | 18 – 88 | [29,123] |
| Probability of adult OI prophylaxis (co-trimoxazole) minor toxicity, one-time % | 17 | 17 | 17 | [29,123] |
| Probability of adult OI prophylaxis (co-trimoxazole) major toxicity, one-time % | 6 | 6 | 6 | [29,123] |
| Probability of virologic suppression (<1,000 c/mL for children, <50 c/mL for adults) at 48 weeks while on ART, one-time % |  |  |  |  |
| Pediatric 1^st^ line ART (LPV/r or EFV-based) | 76 | 76 | 76 | [124–130] |
| Pediatric 2^nd^ and subsequent lines of ART (DTG-based) | 87 | 87 | 87 | [131] |
| Adult 1^st^ line ART (DTG-based) | 90 | 90 | 90 | [132,133] |
| Adult 2^nd^ and subsequent lines of ART (PI-based) | 83 | 83 | 83 | [134] |
| Probability of virologic failure after initial suppression, %/mo. |  |  |  |  |
| Pediatric 1^st^ line ART | 0.52 | 0.52 | 0.52 | [125,128] |
| Pediatric 2^nd^ and subsequent lines of ART | 0.37 | 0.37 | 0.37 | [131] |
| Adult 1^st^ line ART | 0.60 | 0.60 | 0.60 | [132,133,135,136] |
| Adult 2^nd^ and subsequent lines of ART | 0.20 | 0.20 | 0.20 | [137] |
| Probability of resuppression when reinitiating ART, one-time % |  |  |  |  |
| Pediatric ART lines | 31 | 31 | 31 | [138] |
| Adult ART lines | 67 | 67 | 67 | [139,140] |
| Effect of ART on CD4 when suppressed, by age |  |  |  |  |
| 0-4 years, absolute CD4% gain/mo., mean (SD) |  |  |  |  |
| 0-6 months after ART initiation | 2.2 (0.6) | 2.2 (0.6) | 2.2 (0.6) | [141,142] |
| ≥6 months after ART initiation | 0.7 (0.2) | 0.7 (0.2) | 0.7 (0.2) | [141,142] |
| ≥5 years, absolute CD4 gain/mo., mean (SD) |  |  |  |  |
| 0-2 months after ART initiation, cells/mm^3^ | 83.2 (38.2) | 83.2 (38.2) | 83.2 (38.2) | [143] |
| ≥2 months after ART initiation, cells/mm^3^ | 4.2 (1.9) | 4.2 (1.9) | 4.2 (1.9) | [143] |
| Relative risk reduction of HIV-related mortality for patients on ART, by age, % |  |  |  |  |
| 0-5 years | 90 | 90 | 90 | [1] |
| 5-13 years | 90 | 90 | 90 | [1] |
| ≥13 years (range by viral load and CD4) | 55 – 96 | 55 – 96 | 55 – 96 | [20] |
| Relative risk reduction of clinical events for patients on ART, by age, % |  |  |  |  |
| 0-5 years | 85 | 85 | 85 | [1] |
| 5-13 years (range by OI type) | 0 – 85 | 0 – 85 | 0 – 85 | [1] |
| ≥13 years (range by OI type) | 0 – 32 | 0 – 32 | 0 – 32 | [20] |
| Loss to follow-up probability after ART initiation, by age, %/mo. |  |  |  |  |
| 0-13 years | 1.6 | 0.8 | 0.4 | [144–153] |
| ≥13 years | 1.0 | 2.1 | 1.1 | [154–158] |
| Return to care probability after 6 months of loss to follow-up, %/mo. |  |  |  |  |
| With presentation of a severe OI | 100 | 100 | 100 | Assumption |
| Without presentation of a severe OI | 1.3 | 1.3 | 1.3 | [159] |
| Frequency of CD4 monitoring while in care, test interval, mo. |  |  |  | [11] |
| First 12 months post-ART initiation | 6 | 6 | 6 |  |
| After 12 months post-ART initiation | 12 | 12 | 12 |  |
| CD4 threshold to stop CD4 monitoring, cells/mm^3^ | >350 | >350 | >350 |  |
| Frequency of viral load monitoring while in care, mo. |  |  |  | [11] |
| First 12 months post-ART initiation | 6 | 6 | 6 |  |
| After 12 months post-ART initiation | 12 | 12 | 12 |  |
| Costs (in 2019 USD) |  |  |  |  |
| Early infant diagnosis program, $/test |  |  |  |  |
| Nucleic acid amplification test | 25.74 | 25.74 | 25.74 | [33] |
| Antibody test | 4.01 | 4.01 | 4.01 | [160] |
| Negative result return | 2.06 | 2.06 | 2.06 | [161] |
| Positive result return | 3.44 | 3.44 | 3.44 | [161] |
| Pediatric ART (range by age and weight, per mo.) |  |  |  |  |
| 1^st^ line | 10.29 – 18.12 | 10.29 – 18.12 | 10.29 – 18.12 | [35,162] |
| 2^nd^ line | 7.86 – 15.84 | 7.86 – 15.84 | 7.86 – 15.84 | [35,162] |
| Adult ART (per mo.) |  |  |  |  |
| 1^st^ line | 5.30 | 5.30 | 5.30 | [163] |
| 2^nd^ line | 23.15 | 23.15 | 23.15 | [163] |
| Infant oral prophylaxis (NVP +/- ZDV), per mo. | 7.19 – 16.51 | 7.19 – 16.51 | 7.19 – 16.51 | [35,162] |
| bNAb prophylaxis, per dose | 60.00 | 60.00 | 60.00 | [50–53] |
| Routine HIV care, by age, $/mo (range by CD4%/CD4 and ART status) |  |  |  |  |
| 0-18 years | 4.45 – 176.61 | 3.65 – 138.70 | 6.95 – 34.76 | [26–28,31,32] |
| ≥18 years | 4.05 – 27.70 | 3.65 – 138.70 | 6.95 – 34.76 | [27–29,31,32] |
| Routine CD4 monitoring test, $/test | 3.84 | 3.84 | 3.84 | [34,164] |
| Routine HIV viral load test, $/test | 23.75 | 23.75 | 23.75 | [33] |
| Adult OI prophylaxis (co-trimoxazole), $/mo. | 3.97 | 3.97 | 3.97 | [165] |
| Acute OI care (range by OI type), $/mo. |  |  |  |  |
| 0-5 years | 125.15 – 443.15 | 980.97 – 1,723.67 | 0 | [26,30,166] |
| ≥5 years | 80.07 – 557.89 | 219.53 – 729.56 | 0 | [29,31,32] |
| Toxicity, $/event |  |  |  |  |
| Standard of care oral infant prophylaxis, minor (outpatient visit) | 2.67 | 23.84 | 1.91 | [29,31,167] |
| Infant bNAb prophylaxis, minor/major (outpatient visit) | 2.67 | 23.84 | 1.91 | [29,31,167] |
| Adult OI oral prophylaxis (co-trimoxazole), minor (outpatient visit) | 2.67 | 23.84 | 1.91 | [29,31,167] |
| Adult OI oral prophylaxis (co-trimoxazole), major (hospitalization) | 82.05 | 1,480.58 | 51.97 | [29,31,167] |
| Death-related costs, $/death | 87.86 | 541.43 | 0 | [29,31,123,167] |

ART, antiretroviral therapy; mo, month; SD, standard deviation; WHO, World Health Organization; OI, opportunistic infection; NVP, nevirapine; ZDV, zidovudine; bNAb, broadly neutralizing antibody; LPV/r, lopinavir/ritonavir; EFV, efavirenz; DTG, dolutegravir; PI, protease inhibitor.

^†^ All women included in this analysis are known to have HIV infection by delivery. Therefore, we did not model acute HIV infection during breastfeeding.

^‡^ High-risk infants received dual oral infant prophylaxis with nevirapine (NVP) + zidovudine (ZDV) for 12 weeks. Non-high-risk infants received NVP alone for 6 weeks.

^§^ The probability of receiving broadly neutralizing antibody (bNAb) prophylaxis was based on the probability of receiving World Health Organization (WHO) Expanded Program on Immunization vaccines, by age at recommended immunization.

^¶^ A reduction in the risk of intrapartum transmission with use of oral infant prophylaxis was only applied to infants born to mothers who were known to have HIV infection, but who were not on antiretroviral therapy (ART) at delivery. The impact of oral infant prophylaxis on intrapartum transmission among mothers on ART at delivery is already captured in the on ART perinatal transmission estimates.

# **Table A6. Comparison of CEPAC-P infant HIV incidence projections with other nationally representative published estimates.**

| **Country** | **CEPAC-P projections among infants known to be HIV-exposed at birth (*SOC* strategy), %** | **UNAIDS 2020 estimates,**  **% (95% CI)**^†^ | **PHIA data,**  **%** | **Source(s)** |
| --- | --- | --- | --- | --- |
| Côte d’Ivoire | 8.4 | 10.2 (5.8-13.1) | ... ^‡^ | [81] |
| South Africa | 2.8 | 3.3 (3.0-5.2) | ... | [81] |
| Zimbabwe | 6.3 | 8.2 (6.4-11.1) | 6.2 | [79,81] |

SOC, standard of care; CI, confidence interval; PHIA, Population Health Impact Assessment.

^†^ UNAIDS estimates include not only infants born to women known to have HIV, but also infants born to women with HIV whose status is unknown and women who are acutely infected during breastfeeding. Vertical transmission risks from both of those groups of women are higher than from women with HIV whose HIV status is known at delivery. In this analysis, only children born to women known to have HIV at delivery were modeled, so CEPAC-P projected transmission rates are expected to be lower than published UNAIDS rates.

^‡^ Côte d’Ivoire PHIA was reported in March 2021, but it does not contain data regarding vertical transmission rates.

# **Table A7. Clinical and economic outcomes of bNAb administration program, by infant risk status^†^**

| **Country/strategy** | **Clinical outcomes** | |  | **Lifetime efficacy and costs** | | | |
| --- | --- | --- | --- | --- | --- | --- | --- |
|  | **Postnatal cumulative HIV incidence (%)** | **Overall cumulative HIV incidence (%)** |  | **Undiscounted life expectancy (yrs)** | **Discounted life expectancy (yrs)** | **Discounted**  **costs ($)** | **ICER**  **($/YLS)** |
| **Côte d’Ivoire [CET: ICER < $455/YLS (20% GDP per capita), ICER < $1,138 (50% GDP per capita)]** | | | | | | | |
| ***High-risk infants*** |  |  |  |  |  |  |  |
| Standard of care | 5.0 | 13.6 |  | 54.448 | 24.074 | 830 | *Reference* |
| 1 dose | 4.3 | 11.7 |  | 55.162 | 24.365 | 774 | cost-saving |
| 2 doses | 4.0 | 11.4 |  | 55.269 | 24.406 | 789 | 367 |
| Extended | 3.2 | 10.6 |  | 55.482 | 24.484 | 819 | 387^‡,§^ |
| ***Non-high-risk infants*** |  |  |  |  |  |  |  |
| Standard of care | 3.7 | 4.3 |  | 58.929 | 25.916 | 330 | *Reference* ^‡^ |
| 1 dose | 3.6 | 4.0 |  | 59.015 | 25.952 | 357 | dominated |
| 2 doses | 3.3 | 3.7 |  | 59.118 | 25.992 | 378 | dominated |
| Extended | 2.4 | 2.9 |  | 59.340 | 26.073 | 408 | 497^§^ |
| **South Africa [CET: ICER < $1,200/YLS (20% GDP per capita), ICER < $3,001 (50% GDP per capita)]** | | | | | | | |
| ***High-risk infants*** |  |  |  |  |  |  |  |
| Standard of care | 1.5 | 7.9 |  | 65.282 | 27.218 | 619 | *Reference* |
| 1 dose | 1.1 | 6.3 |  | 66.057 | 27.485 | 546 | cost-saving |
| 2 doses | 0.9 | 6.1 |  | 66.153 | 27.517 | 559 | dominated |
| Extended | 0.7 | 5.9 |  | 66.249 | 27.548 | 570 | 377^‡,§^ |
| ***Non-high-risk infants*** |  |  |  |  |  |  |  |
| Standard of care | 1.0 | 1.6 |  | 68.972 | 28.522 | 162 | *Reference* |
| 1 dose | 0.9 | 1.3 |  | 69.077 | 28.559 | 185 | dominated |
| 2 doses | 0.7 | 1.2 |  | 69.165 | 28.590 | 198 | dominated |
| Extended | 0.5 | 1.0 |  | 69.277 | 28.626 | 210 | 464^‡,§^ |
| **Zimbabwe [CET: ICER < $293/YLS (20% GDP per capita), ICER < $732 (50% GDP per capita)]** | | | | | | | |
| ***High-risk infants*** |  |  |  |  |  |  |  |
| Standard of care | 4.8 | 13.7 |  | 61.384 | 25.504 | 838 | *Reference* |
| 1 dose | 3.8 | 11.3 |  | 62.535 | 25.92 | 754 | cost-saving^‡^ |
| 2 doses | 3.4 | 10.9 |  | 62.714 | 25.983 | 777 | 367 |
| Extended | 2.5 | 9.9 |  | 63.088 | 26.103 | 824 | 389^§^ |
| ***Non-high-risk infants*** |  |  |  |  |  |  |  |
| Standard of care | 3.3 | 3.8 |  | 67.281 | 27.694 | 272 | *Reference*^‡^ |
| 1 dose | 3.1 | 3.6 |  | 67.424 | 27.745 | 304 | dominated |
| 2 doses | 2.8 | 3.2 |  | 67.576 | 27.798 | 331 | dominated |
| Extended | 1.7 | 2.2 |  | 67.993 | 27.931 | 380 | 455^§^ |

Pediatric HIV incidence is rounded to the nearest tenth of a percent. Undiscounted and discounted life expectancies are rounded to the nearest ten thousandth. Costs are rounded to the nearest dollar and are presented in 2019 USD. Discounted values are discounted at 3% per year. ICERs are rounded to the nearest dollar and are calculated using unrounded discounted life expectancy and discounted costs. The most cost-effective bNAb implementation strategy was the strategy that offered the greatest increase in overall population life expectancy while still having an ICER less than the cost-effectiveness threshold when compared to the next best performing, non-dominated strategy.

bNAb, broadly neutralizing antibody; yr, year; ICER, incremental cost-effectiveness ratio; YLS, year of life saved; CET, cost-effectiveness threshold.

^†^ Results are presented for each sub-cohort (i.e., high-risk and non-high-risk infants) separately. When weighted and combined, these results would reflect the base case results presented in Table 2. ICERs compare implementation strategies within a single cohort (i.e., high-risk or non-high-risk) not for the overall population.

^‡^ Indicates the most cost-effective strategy at a cost-effectiveness threshold of 20% GDP per capita.

^§^ Indicates the most cost-effective strategy at a cost-effectiveness threshold of 50% GDP per capita.

# **Table A8. Scenario analysis: bNAb does not reduce intrapartum transmission (base case: 60% reduction)**

| **Country/strategy** | **Clinical outcomes** | | | **Lifetime efficacy and costs** | | | |
| --- | --- | --- | --- | --- | --- | --- | --- |
|  | **IU/IP cumulative HIV incidence (%)** | **Postnatal cumulative HIV incidence (%)** | **Total cumulative HIV incidence (%)** | **Undiscounted life expectancy (yrs)** | **Discounted life expectancy (yrs)** | **Discounted costs ($)** | **ICER ($/YLS)** |
| **Côte d’Ivoire [CET: ICER < $455/YLS (20% GDP per capita), ICER < $1,138 (50% GDP per capita)]** | | | | | | | |
| Standard of care | 4.1 | 4.3 | 8.4 | 56.960 | 25.106 | 550 | *Reference* |
| HR-HIVE – 1 dose | 4.1 | 4.0 | 8.1 | 57.080 | 25.156 | 550 | 9 |
| HIVE – 1 dose | 4.1 | 3.9 | 8.0 | 57.113 | 25.170 | 567 | dominated |
| HR-HIVE – 2 doses | 4.1 | 3.8 | 7.9 | 57.127 | 25.174 | 557 | 374 |
| HR-HIVE – Extended | 4.1 | 3.5 | 7.6 | 57.220 | 25.208 | 570 | 390^†^ |
| HIVE – 2 doses | 4.1 | 3.6 | 7.7 | 57.217 | 25.210 | 585 | dominated |
| HIVE – Extended | 4.1 | 2.8 | 6.8 | 57.434 | 25.289 | 616 | 564^‡^ |
| **South Africa [CET: ICER < $1,200/YLS (20% GDP per capita), ICER < $3,001 (50% GDP per capita)]** | | | | | | | |
| Standard of care | 1.7 | 1.1 | 2.8 | 68.270 | 28.274 | 249 | *Reference* |
| HR-HIVE – 1 dose | 1.7 | 1.0 | 2.7 | 68.308 | 28.288 | 251 | 165 |
| HR-HIVE – 2 doses | 1.7 | 1.0 | 2.7 | 68.327 | 28.294 | 254 | dominated |
| HR-HIVE – Extended | 1.7 | 1.0 | 2.6 | 68.345 | 28.300 | 256 | 365 |
| HIVE – 1 dose | 1.7 | 1.0 | 2.6 | 68.354 | 28.304 | 276 | dominated |
| HIVE – 2 doses | 1.7 | 0.8 | 2.4 | 68.443 | 28.335 | 289 | dominated |
| HIVE – Extended | 1.7 | 0.6 | 2.2 | 68.551 | 28.370 | 300 | 636^†,‡^ |
| **Zimbabwe [CET: ICER < $293/YLS (20% GDP per capita), ICER < $732 (50% GDP per capita)]** | | | | | | | |
| Standard of care | 2.6 | 3.7 | 6.3 | 65.837 | 27.158 | 411 | *Reference* |
| HR-HIVE – 1 dose | 2.6 | 3.4 | 6.0 | 65.953 | 27.199 | 410 | cost-saving^†^ |
| HR-HIVE – 2 doses | 2.6 | 3.3 | 5.9 | 65.996 | 27.214 | 415 | 376 |
| HIVE – 1 dose | 2.6 | 3.3 | 5.9 | 66.029 | 27.226 | 438 | dominated |
| HR-HIVE – Extended | 2.6 | 3.1 | 5.7 | 66.086 | 27.243 | 427 | 404 |
| HIVE – 2 doses | 2.6 | 2.9 | 5.5 | 66.187 | 27.281 | 464 | dominated |
| HIVE - Extended | 2.6 | 1.9 | 4.5 | 66.591 | 27.411 | 512 | 510^‡^ |

Pediatric HIV incidence is rounded to the nearest tenth of a percent. Undiscounted and discounted life expectancies are rounded to the nearest ten thousandth. Costs are rounded to the nearest dollar and are presented in 2019 USD. Discounted values are discounted at 3% per year. ICERs are rounded to the nearest dollar and are calculated using unrounded discounted life expectancy and discounted costs. The most cost-effective bNAb implementation strategy was the strategy that offered the greatest increase in overall population life expectancy while still having an ICER less than the cost-effectiveness threshold when compared to the next best performing, non-dominated strategy.

bNAb, broadly neutralizing antibody; IU/IP, intrauterine/intrapartum; yr, year; ICER, incremental cost-effectiveness ratio; YLS, years of life saved; CET, cost-effectiveness threshold; HR-HIVE, high-risk HIV-exposed infants; HIVE, all HIV-exposed infants.

^†^ Indicates the most cost-effective strategy at a cost-effectiveness threshold of 20% GDP per capita.

^‡^ Indicates the most cost-effective strategy at a cost-effectiveness threshold of 50% GDP per capita.

# **Table A9. Scenario analysis: intrapartum transmissions accounts for 67% of all perinatal transmissions (base case: 33%)**

| **Country/strategy** | **Clinical outcomes** | | | **Lifetime efficacy and costs** | | | |
| --- | --- | --- | --- | --- | --- | --- | --- |
|  | **IU/IP cumulative HIV incidence (%)** | **Postnatal cumulative HIV incidence (%)** | **Total cumulative HIV incidence (%)** | **Undiscounted life expectancy (yrs)** | **Discounted life expectancy (yrs)** | **Discounted costs ($)** | **ICER ($/YLS)** |
| **Côte d’Ivoire [CET: ICER < $455/YLS (20% GDP per capita), ICER < $1,138 (50% GDP per capita)]** | | | | | | | |
| Standard of care | 4.1 | 4.3 | 8.4 | 56.956 | 25.105 | 549 | *Reference* |
| HR-HIVE – 1 dose | 3.0 | 4.0 | 7.0 | 57.465 | 25.312 | 500 | cost-saving |
| HR-HIVE – 2 doses | 3.0 | 3.9 | 6.9 | 57.513 | 25.330 | 507 | dominated |
| HIVE – 1 dose | 2.9 | 3.9 | 6.9 | 57.529 | 25.339 | 513 | dominated |
| HR-HIVE – Extended | 3.0 | 3.5 | 6.6 | 57.608 | 25.364 | 520 | 382 |
| HIVE – 2 doses | 2.9 | 3.6 | 6.6 | 57.635 | 25.379 | 531 | dominated |
| HIVE – Extended | 2.9 | 2.8 | 5.7 | 57.854 | 25.459 | 561 | 439^†,‡^ |
| **South Africa [CET: ICER < $1,200/YLS (20% GDP per capita), ICER < $3,001 (50% GDP per capita)]** | | | | | | | |
| Standard of care | 1.7 | 1.1 | 2.8 | 68.249 | 28.262 | 243 | *Reference* |
| HR-HIVE – 1 dose | 1.2 | 1.1 | 2.2 | 68.511 | 28.354 | 215 | cost-saving |
| HR-HIVE – 2 doses | 1.2 | 1.0 | 2.2 | 68.530 | 28.360 | 217 | dominated |
| HR-HIVE – Extended | 1.2 | 1.0 | 2.2 | 68.548 | 28.366 | 219 | dominated |
| HIVE – 1 dose | 1.0 | 1.0 | 2.0 | 68.637 | 28.398 | 228 | 312 |
| HIVE – 2 doses | 1.0 | 0.8 | 1.8 | 68.727 | 28.429 | 241 | dominated |
| HIVE – Extended | 1.0 | 0.6 | 1.6 | 68.836 | 28.464 | 252 | 363^†,‡^ |
| **Zimbabwe [CET: ICER < $293/YLS (20% GDP per capita), ICER < $732 (50% GDP per capita)]** | | | | | | | |
| Standard of care | 2.6 | 3.7 | 6.3 | 65.832 | 27.155 | 408 | *Reference* |
| HR-HIVE – 1 dose | 1.9 | 3.5 | 5.3 | 66.281 | 27.318 | 369 | cost-saving^†^ |
| HR-HIVE – 2 doses | 1.9 | 3.4 | 5.2 | 66.325 | 27.333 | 374 | 364 |
| HR-HIVE – Extended | 1.9 | 3.1 | 5.0 | 66.419 | 27.363 | 386 | 390 |
| HIVE – 1 dose | 1.7 | 3.3 | 5.0 | 66.421 | 27.368 | 389 | dominated |
| HIVE – 2 doses | 1.7 | 3.0 | 4.7 | 66.580 | 27.423 | 416 | dominated |
| HIVE – Extended | 1.7 | 1.9 | 3.6 | 66.988 | 27.554 | 465 | 412^‡^ |

Pediatric HIV incidence is rounded to the nearest tenth of a percent. Undiscounted and discounted life expectancies are rounded to the nearest ten thousandth. Costs are rounded to the nearest dollar and are presented in 2019 USD. Discounted values are discounted at 3% per year. ICERs are rounded to the nearest dollar and are calculated using unrounded discounted life expectancy and discounted costs. The most cost-effective bNAb implementation strategy was the strategy that offered the greatest increase in overall population life expectancy while still having an ICER less than the cost-effectiveness threshold when compared to the next best performing, non-dominated strategy.

bNAb, broadly neutralizing antibody; IU/IP, intrauterine/intrapartum; yr, year; ICER, incremental cost-effectiveness ratio; YLS, years of life saved; CET, cost-effectiveness threshold; HR-HIVE, high-risk HIV-exposed infants; HIVE, all HIV-exposed infants.

^†^ Indicates the most cost-effective strategy at a cost-effectiveness threshold of 20% GDP per capita.

^‡^ Indicates the most cost-effective strategy at a cost-effectiveness threshold of 50% GDP per capita.

# **Table A10. Scenario analysis:** **proportion of high-risk infants recognized as being high-risk (base case: 100%)^†^**

| **Country/strategy** | **Clinical outcomes** |  | **Lifetime efficacy and costs** | | |
| --- | --- | --- | --- | --- | --- |
|  | **Total cumulative HIV incidence (%)** |  | **Discounted life expectancy (yrs)** | **Discounted**  **costs ($)** | **ICER ($/YLS)** |
| **Côte d’Ivoire [CET: ICER < $455/YLS (20% GDP per capita), ICER < $1,138 (50% GDP per capita)]** | | | | | |
| ***Proportion recognized as high-risk: 10%*** [99] | | |  |  |  |
| Standard of care | 8.9 |  | 25.033 | 567 | *Reference* |
| HR-HIVE – 1 dose | 8.8 |  | 25.046 | 565 | cost-saving |
| HR-HIVE – 2 doses | 8.8 |  | 25.048 | 565 | 367 |
| HIVE – 1 dose | 8.8 |  | 25.051 | 567 | 387^‡^ |
| HR-HIVE – Extended | 8.7 |  | 25.067 | 580 | dominated |
| HIVE – 2 doses | 8.5 |  | 25.091 | 592 | dominated |
| HIVE – Extended | 8.0 |  | 25.139 | 610 | 497^§^ |
| ***Proportion recognized as high-risk: 50%*** | | |  |  |  |
| Standard of care | 8.7 |  | 25.066 | 559 | *Reference* |
| HR-HIVE – 1 dose | 8.3 |  | 25.130 | 547 | cost-saving |
| HR-HIVE – 2 doses | 8.2 |  | 25.139 | 550 | 367 |
| HIVE – 1 dose | 8.1 |  | 25.150 | 562 | dominated |
| HR-HIVE – Extended | 8.0 |  | 25.156 | 557 | 387^‡^ |
| HIVE – 2 doses | 7.9 |  | 25.181 | 577 | dominated |
| HIVE – Extended | 7.2 |  | 25.244 | 601 | 497^§^ |
| **South Africa [CET: ICER < $1,200/YLS (20% GDP per capita), ICER < $3,001 (50% GDP per capita)]** | | | | | |
| ***Proportion recognized as high-risk: 10%*** [99] | | |  |  |  |
| Standard of care | 2.9 |  | 28.259 | 251 | *Reference* |
| HR-HIVE – 1 dose | 2.8 |  | 28.264 | 249 | cost-saving |
| HR-HIVE – 2 doses | 2.8 |  | 28.265 | 250 | dominated |
| HR-HIVE – Extended | 2.8 |  | 28.265 | 250 | 377 |
| HIVE – 1 dose | 2.7 |  | 28.294 | 268 | dominated |
| HIVE – 2 doses | 2.5 |  | 28.320 | 279 | dominated |
| HIVE – Extended | 2.3 |  | 28.349 | 289 | 464^‡,§^ |
| ***Proportion recognized as high-risk: 50%*** | | |  |  |  |
| Standard of care | 2.8 |  | 28.266 | 250 | *Reference* |
| HR-HIVE – 1 dose | 2.7 |  | 28.291 | 243 | cost-saving |
| HR-HIVE – 2 doses | 2.7 |  | 28.294 | 244 | dominated |
| HR-HIVE – Extended | 2.6 |  | 28.297 | 245 | 377 |
| HIVE – 1 dose | 2.5 |  | 28.321 | 262 | dominated |
| HIVE – 2 doses | 2.3 |  | 28.349 | 273 | dominated |
| HIVE – Extended | 2.1 |  | 28.381 | 284 | 464^‡,§^ |
| **Zimbabwe [CET: ICER < $293/YLS (20% GDP per capita), ICER < $732 (50% GDP per capita)]** | | | | | |
| ***Proportion recognized as high-risk: 10%*** [99] | | |  |  |  |
| Standard of care | 6.6 |  | 27.107 | 420 | *Reference* |
| HR-HIVE – 1 dose | 6.5 |  | 27.117 | 418 | cost-saving^‡^ |
| HR-HIVE – 2 doses | 6.5 |  | 27.118 | 418 | 367 |
| HR-HIVE – Extended | 6.5 |  | 27.121 | 419 | 389 |
| HIVE – 1 dose | 6.3 |  | 27.155 | 442 | dominated |
| HIVE – 2 doses | 6.0 |  | 27.197 | 463 | dominated |
| HIVE – Extended | 5.2 |  | 27.300 | 501 | 455^§^ |
| ***Proportion recognized as high-risk: 50%*** | | |  |  |  |
| Standard of care | 6.4 |  | 27.129 | 416 | *Reference* |
| HR-HIVE – 1 dose | 6.1 |  | 27.180 | 405 | cost-saving^‡^ |
| HR-HIVE – 2 doses | 6.1 |  | 27.188 | 408 | 367 |
| HR-HIVE – Extended | 6.0 |  | 27.203 | 414 | 389 |
| HIVE – 1 dose | 5.9 |  | 27.219 | 429 | dominated |
| HIVE – 2 doses | 5.6 |  | 27.266 | 453 | dominated |
| HIVE – Extended | 4.7 |  | 27.382 | 495 | 455^§^ |

The preferred bNAb implementation strategy was the strategy that offered the greatest increase in overall population life expectancy while still having an ICER less than the cost-effectiveness threshold when compared to the next best performing, non-dominated strategy.

yr: year; ICER: incremental cost-effectiveness ratio; YLS: year of life saved; CET: cost-effectiveness threshold; HR-HIVE: high-risk HIV-exposed infants; HIVE: all HIV-exposed infants.

^†^ In addition to receiving bNAbs in *HR-HIVE* strategies, high-risk infants recognized as such also receive 12 weeks of twice daily zidovudine and once daily nevirapine. High-risk infants unrecognized as being high-risk only receive 6 weeks of once daily nevirapine.

^‡^ Indicates the preferred strategy at a cost-effectiveness threshold of 20% GDP per capita.

^§^ Indicates the preferred strategy at a cost-effectiveness threshold of 50% GDP per capita.

# **Table A11. One-way sensitivity analysis: bNAb efficacy against intrapartum and postpartum transmission**

| **Country/strategy** | **Clinical outcomes** |  | **Lifetime efficacy and costs** | | |
| --- | --- | --- | --- | --- | --- |
|  | **Total cumulative HIV incidence (%)** |  | **Discounted life expectancy (yrs)** | **Discounted**  **costs ($)** | **ICER ($/YLS)** |
| **Côte d’Ivoire [CET: ICER < $455/YLS (20% GDP per capita), ICER < $1,138 (50% GDP per capita)]** | | | | | |
| ***bNAb efficacy: 10%*** | | |  |  |  |
| Standard of care | 8.4 |  | 25.106 | 550 | *Reference*^†^ |
| HR-HIVE – 1 dose | 8.3 |  | 25.129 | 561 | 511^‡^ |
| HR-HIVE – 2 doses | 8.2 |  | 25.131 | 576 | dominated |
| HIVE – 1 dose | 8.2 |  | 25.133 | 582 | dominated |
| HR-HIVE – Extended | 8.2 |  | 25.137 | 610 | dominated |
| HIVE – 2 doses | 8.2 |  | 25.139 | 617 | 5,334 |
| HIVE – Extended | 8.1 |  | 25.151 | 698 | 6,904 |
| ***bNAb efficacy: 100%*** | | |  |  |  |
| Standard of care | 8.4 |  | 25.106 | 550 | *Reference* |
| HR-HIVE – 1 dose | 7.0 |  | 25.321 | 469 | cost-saving |
| HR-HIVE – 2 doses | 6.7 |  | 25.352 | 496 | dominated |
| HIVE – 1 dose | 6.8 |  | 25.355 | 479 | dominated |
| HR-HIVE – Extended | 6.2 |  | 25.409 | 492 | dominated |
| HIVE – 2 doses | 6.2 |  | 25.422 | 511 | dominated |
| HIVE – Extended | 4.9 |  | 25.557 | 499 | 128^†,‡^ |
| **South Africa [CET: ICER < $1,200/YLS (20% GDP per capita), ICER < $3,001 (50% GDP per capita)]** | | | | | |
| ***bNAb efficacy: 10%*** | | |  |  |  |
| Standard of care | 2.8 |  | 28.274 | 249 | *Reference* |
| HR-HIVE – 1 dose | 2.7 |  | 28.282 | 253 | 429^†,‡^ |
| HR-HIVE – 2 doses | 2.7 |  | 28.283 | 257 | 3,455 |
| HR-HIVE – Extended | 2.7 |  | 28.284 | 261 | dominated |
| HIVE – 1 dose | 2.7 |  | 28.285 | 281 | dominated |
| HIVE – 2 doses | 2.7 |  | 28.292 | 303 | dominated |
| HIVE – Extended | 2.6 |  | 28.298 | 327 | 5,007 |
| ***bNAb efficacy: 100%*** | | |  |  |  |
| Standard of care | 2.8 |  | 28.274 | 249 | *Reference* |
| HR-HIVE – 1 dose | 2.3 |  | 28.359 | 221 | cost-saving |
| HR-HIVE – 2 doses | 2.2 |  | 28.369 | 222 | dominated |
| HR-HIVE – Extended | 2.1 |  | 28.380 | 222 | 56 |
| HIVE – 1 dose | 2.0 |  | 28.410 | 232 | dominated |
| HIVE – 2 doses | 1.7 |  | 28.461 | 238 | dominated |
| HIVE – Extended | 1.3 |  | 28.520 | 239 | 121^†,‡^ |
| **Zimbabwe [CET: ICER < $293/YLS (20% GDP per capita), ICER < $732 (50% GDP per capita)]** | | | | | |
| ***bNAb efficacy: 10%*** | | |  |  |  |
| Standard of care | 6.3 |  | 27.158 | 411 | *Reference*^†^ |
| HR-HIVE – 1 dose | 6.2 |  | 27.175 | 417 | 365^‡^ |
| HR-HIVE – 2 doses | 6.1 |  | 27.178 | 427 | 3,519 |
| HR-HIVE – Extended | 6.1 |  | 27.182 | 451 | dominated |
| HIVE – 1 dose | 6.1 |  | 27.183 | 450 | 4,419 |
| HIVE – 2 doses | 6.1 |  | 27.192 | 492 | 4,905 |
| HIVE – Extended | 5.9 |  | 27.210 | 597 | 5,644 |
| ***bNAb efficacy: 100%*** | | |  |  |  |
| Standard of care | 6.3 |  | 27.158 | 411 | *Reference* |
| HR-HIVE – 1 dose | 5.3 |  | 27.328 | 368 | cost-saving |
| HR-HIVE – 2 doses | 5.1 |  | 27.354 | 370 | dominated |
| HIVE – 1 dose | 4.9 |  | 27.391 | 385 | dominated |
| HR-HIVE – Extended | 4.7 |  | 27.405 | 371 | 41 |
| HIVE – 2 doses | 4.3 |  | 27.483 | 398 | dominated |
| HIVE – Extended | 2.6 |  | 27.707 | 400 | 95^†,‡^ |

The preferred bNAb implementation strategy was the strategy that offered the greatest increase in overall population life expectancy while still having an ICER less than the cost-effectiveness threshold when compared to the next best performing, non-dominated strategy.

yr, year; ICER, incremental cost-effectiveness ratio; YLS, year of life saved; CET, cost-effectiveness threshold; HR-HIVE, high-risk HIV-exposed infants; HIVE, all HIV-exposed infants.

^†^ Indicates the preferred strategy at a cost-effectiveness threshold of 20% GDP per capita.

^‡^ Indicates the preferred strategy at a cost-effectiveness threshold of 50% GDP per capita.

# **Table A12. One-way sensitivity analysis: bNAb cost**

| **Country/strategy** | **Clinical outcomes** |  | **Lifetime efficacy and costs** | | |
| --- | --- | --- | --- | --- | --- |
|  | **Total cumulative HIV incidence (%)** |  | **Discounted life expectancy (yrs)** | **Discounted**  **costs ($)** | **ICER ($/YLS)** |
| **Côte d’Ivoire [CET: ICER < $455/YLS (20% GDP per capita), ICER < $1,138 (50% GDP per capita)]** | | | | | |
| ***bNAb cost: $20/dose*** | | |  |  |  |
| Standard of care | 8.4 |  | 25.106 | 550 | *Reference* |
| HR-HIVE – 1 dose | 7.6 |  | 25.234 | 513 | dominated |
| HR-HIVE – 2 doses | 7.4 |  | 25.252 | 509 | dominated |
| HIVE – 1 dose | 7.4 |  | 25.255 | 513 | dominated |
| HR-HIVE – Extended | 7.1 |  | 25.286 | 497 | dominated |
| HIVE – 2 doses | 7.1 |  | 25.295 | 507 | dominated |
| HIVE – Extended | 6.3 |  | 25.374 | 476 | cost-saving^†,‡^ |
| ***bNAb cost: $200/dose*** | | |  |  |  |
| Standard of care | 8.4 |  | 25.106 | 550 | *Reference* |
| HR-HIVE – 1 dose | 7.6 |  | 25.234 | 566 | 124^†,‡^ |
| HR-HIVE – 2 doses | 7.4 |  | 25.252 | 610 | 2,499 |
| HIVE – 1 dose | 7.4 |  | 25.255 | 632 | dominated |
| HR-HIVE – Extended | 7.1 |  | 25.286 | 713 | dominated |
| HIVE – 2 doses | 7.1 |  | 25.295 | 739 | 2,998 |
| HIVE – Extended | 6.3 |  | 25.374 | 981 | 3,060 |
| **South Africa [CET: ICER < $1,200/YLS (20% GDP per capita), ICER < $3,001 (50% GDP per capita)]** | | | | | |
| ***bNAb cost: $20/dose*** | | |  |  |  |
| Standard of care | 2.8 |  | 28.274 | 249 | *Reference* |
| HR-HIVE – 1 dose | 2.5 |  | 28.325 | 231 | dominated |
| HR-HIVE – 2 doses | 2.4 |  | 28.331 | 231 | dominated |
| HR-HIVE – Extended | 2.4 |  | 28.337 | 229 | dominated |
| HIVE – 1 dose | 2.3 |  | 28.355 | 233 | dominated |
| HIVE – 2 doses | 2.1 |  | 28.386 | 231 | dominated |
| HIVE – Extended | 1.9 |  | 28.421 | 224 | cost-saving^†,‡^ |
| ***bNAb cost: $200/dose*** | | |  |  |  |
| Standard of care | 2.8 |  | 28.274 | 249 | *Reference* |
| HR-HIVE – 1 dose | 2.5 |  | 28.325 | 249 | cost-saving^†^ |
| HR-HIVE – 2 doses | 2.4 |  | 28.331 | 262 | 2,083 |
| HR-HIVE – Extended | 2.4 |  | 28.337 | 275 | 2,251 |
| HIVE – 1 dose | 2.3 |  | 28.355 | 327 | dominated |
| HIVE – 2 doses | 2.1 |  | 28.386 | 395 | dominated |
| HIVE – Extended | 1.9 |  | 28.421 | 470 | 2,321^‡^ |
| **Zimbabwe [CET: ICER < $293/YLS (20% GDP per capita), ICER < $732 (50% GDP per capita)]** | | | | | |
| ***bNAb cost: $20/dose*** | | |  |  |  |
| Standard of care | 6.3 |  | 27.158 | 411 | *Reference* |
| HR-HIVE – 1 dose | 5.7 |  | 27.260 | 382 | dominated |
| HR-HIVE – 2 doses | 5.6 |  | 27.275 | 381 | dominated |
| HIVE – 1 dose | 5.4 |  | 27.298 | 383 | dominated |
| HR-HIVE – Extended | 5.3 |  | 27.304 | 375 | dominated |
| HIVE – 2 doses | 5.1 |  | 27.353 | 379 | dominated |
| HIVE – Extended | 4.1 |  | 27.484 | 350 | cost-saving^†,‡^ |
| ***bNAb cost: $200/dose*** | | |  |  |  |
| Standard of care | 6.3 |  | 27.158 | 411 | *Reference* |
| HR-HIVE – 1 dose | 5.7 |  | 27.260 | 416 | 58^†,‡^ |
| HR-HIVE – 2 doses | 5.6 |  | 27.275 | 447 | 1,994 |
| HIVE – 1 dose | 5.4 |  | 27.298 | 521 | dominated |
| HR-HIVE – Extended | 5.3 |  | 27.304 | 522 | dominated |
| HIVE – 2 doses | 5.1 |  | 27.353 | 652 | dominated |
| HIVE – Extended | 4.1 |  | 27.484 | 975 | 2,531 |

The preferred bNAb implementation strategy was the strategy that offered the greatest increase in overall population life expectancy while still having an ICER less than the cost-effectiveness threshold when compared to the next best performing, non-dominated strategy.

yr, year; ICER, incremental cost-effectiveness ratio; YLS, year of life saved; CET, cost-effectiveness threshold; HR-HIVE, high-risk HIV-exposed infants; HIVE, all HIV-exposed infants.

^†^ Indicates the preferred strategy at a cost-effectiveness threshold of 20% GDP per capita.

^‡^ Indicates the preferred strategy at a cost-effectiveness threshold of 50% GDP per capita.

# **Table A13. One-way sensitivity analysis: bNAb effect duration^†^**

| **Country/strategy** | **Clinical outcomes** |  | **Lifetime efficacy and costs** | | |
| --- | --- | --- | --- | --- | --- |
|  | **Total cumulative HIV incidence (%)** |  | **Discounted life expectancy (yrs)** | **Discounted**  **costs ($)** | **ICER ($/YLS)** |
| **Côte d’Ivoire [CET: ICER < $455/YLS (20% GDP per capita), ICER < $1,138 (50% GDP per capita)]** | | | | | |
| ***bNAb effect duration: 1 month*** | | |  |  |  |
| Standard of care | 8.4 |  | 25.106 | 550 | *Reference* |
| HR-HIVE – 1 dose | 7.8 |  | 25.203 | 537 | cost-saving^‡^ |
| HIVE – 1 dose | 7.7 |  | 25.212 | 557 | dominated |
| HR-HIVE – 2 doses | 7.7 |  | 25.218 | 548 | 671^§^ |
| HIVE – 2 doses | 7.6 |  | 25.232 | 587 | dominated |
| HR-HIVE – Extended | 7.1 |  | 25.283 | 668 | 1,867 |
| HIVE – Extended | 6.3 |  | 25.368 | 878 | 2,459 |
| ***bNAb effect duration: 6 months*** | | |  |  |  |
| Standard of care | 8.4 |  | 25.106 | 550 | *Reference* |
| HR-HIVE – 1 dose | 7.4 |  | 25.253 | 516 | dominated |
| HR-HIVE – 2 doses | 7.2 |  | 25.275 | 513 | dominated |
| HR-HIVE – Extended | 7.0 |  | 25.289 | 512 | dominated |
| HIVE – 1 dose | 7.1 |  | 25.294 | 521 | dominated |
| HIVE – 2 doses | 6.6 |  | 25.347 | 514 | dominated |
| HIVE – Extended | 6.2 |  | 25.380 | 512 | cost-saving^‡,§^ |
| **South Africa [CET: ICER < $1,200/YLS (20% GDP per capita), ICER < $3,001 (50% GDP per capita)]** | | | | | |
| ***bNAb effect duration: 1 month*** | | |  |  |  |
| Standard of care | 2.8 |  | 28.274 | 249 | *Reference* |
| HR-HIVE – 1 dose | 2.5 |  | 28.316 | 238 | cost-saving |
| HR-HIVE – 2 doses | 2.5 |  | 28.321 | 242 | 788^‡^ |
| HR-HIVE – Extended | 2.4 |  | 28.330 | 261 | dominated |
| HIVE – 1 dose | 2.4 |  | 28.336 | 264 | 1,423 |
| HIVE – 2 doses | 2.4 |  | 28.341 | 287 | dominated |
| HIVE – Extended | 1.9 |  | 28.417 | 411 | 1,817^§^ |
| ***bNAb effect duration: 6 months*** | | |  |  |  |
| Standard of care | 2.8 |  | 28.274 | 249 | *Reference* |
| HR-HIVE – 1 dose | 2.4 |  | 28.331 | 233 | cost-saving |
| HR-HIVE – 2 doses | 2.4 |  | 28.336 | 233 | 69 |
| HR-HIVE – Extended | 2.4 |  | 28.337 | 233 | dominated |
| HIVE – 1 dose | 2.1 |  | 28.385 | 243 | dominated |
| HIVE – 2 doses | 1.9 |  | 28.416 | 244 | dominated |
| HIVE – Extended | 1.9 |  | 28.423 | 245 | 131^‡,§^ |
| **Zimbabwe [CET: ICER < $293/YLS (20% GDP per capita), ICER < $732 (50% GDP per capita)]** | | | | | |
| ***bNAb effect duration: 1 month*** | | |  |  |  |
| Standard of care | 6.3 |  | 27.158 | 411 | *Reference* |
| HR-HIVE – 1 dose | 5.8 |  | 27.232 | 398 | cost-saving^‡^ |
| HR-HIVE – 2 doses | 5.7 |  | 27.246 | 405 | 488^§^ |
| HIVE – 1 dose | 5.7 |  | 27.249 | 428 | dominated |
| HIVE – 2 doses | 5.6 |  | 27.271 | 467 | dominated |
| HR-HIVE – Extended | 5.3 |  | 27.303 | 491 | 1,502 |
| HIVE – Extended | 4.1 |  | 27.480 | 846 | 2,002 |
| ***bNAb effect duration: 6 months*** | | |  |  |  |
| Standard of care | 6.3 |  | 27.158 | 411 | *Reference* |
| HR-HIVE – 1 dose | 5.6 |  | 27.274 | 385 | dominated |
| HR-HIVE – 2 doses | 5.4 |  | 27.293 | 385 | cost-saving |
| HR-HIVE – Extended | 5.3 |  | 27.305 | 386 | dominated |
| HIVE – 1 dose | 5.1 |  | 27.350 | 395 | dominated |
| HIVE – 2 doses | 4.5 |  | 27.434 | 394 | 64 |
| HIVE – Extended | 4.1 |  | 27.488 | 398 | 74^‡,§^ |

The preferred bNAb implementation strategy was the strategy that offered the greatest increase in overall population life expectancy while still having an ICER less than the cost-effectiveness threshold when compared to the next best performing, non-dominated strategy.

yr, year; ICER, incremental cost-effectiveness ratio; YLS, year of life saved; CET, cost-effectiveness threshold; HR-HIVE, high-risk HIV-exposed infants; HIVE, all HIV-exposed infants.

^†^ The bNAb dosing frequency was adjusted accordingly based on the tested bNAb effect duration. For example, when bNAb effect duration was reduced to 1 month, the *2 dose* strategy provided bNAbs at birth and 1 month, while the *Extended* strategy provided bNAbs every month for the duration of breastfeeding.

^‡^ Indicates the preferred strategy at a cost-effectiveness threshold of 20% GDP per capita.

^§^ Indicates the preferred strategy at a cost-effectiveness threshold of 50% GDP per capita.

# **Table A14. One-way sensitivity analysis: bNAb toxicity**

| **Country/strategy** | **Clinical outcomes** |  | **Lifetime efficacy and costs** | | |  |
| --- | --- | --- | --- | --- | --- | --- |
|  | **Total cumulative HIV incidence (%)** |  | **Discounted life expectancy (yrs)** | **Discounted**  **costs ($)** | **ICER ($/YLS)** | |
| **Côte d’Ivoire [CET: ICER < $455/YLS (20% GDP per capita), ICER < $1,138 (50% GDP per capita)]** | | | | | |  |
| ***bNAb major toxicity one-time probability: 0%*** | | |  |  |  |  |
| Standard of care | 8.4 |  | 25.106 | 550 | *Reference* | |
| HR-HIVE – 1 dose | 7.6 |  | 25.234 | 525 | cost-saving | |
| HR-HIVE – 2 doses | 7.4 |  | 25.252 | 532 | 367 | |
| HIVE – 1 dose | 7.4 |  | 25.255 | 540 | dominated | |
| HR-HIVE – Extended | 7.1 |  | 25.286 | 545 | 387^†^ | |
| HIVE – 2 doses | 7.1 |  | 25.295 | 559 | dominated | |
| HIVE – Extended | 6.3 |  | 25.374 | 589 | 497^‡^ | |
| ***bNAb major toxicity one-time probability: 5%*** | | |  |  |  |  |
| Standard of care | 8.4 |  | 25.106 | 550 | *Reference* | |
| HR-HIVE – 1 dose | 7.6 |  | 25.234 | 525 | cost-saving | |
| HR-HIVE – 2 doses | 7.4 |  | 25.251 | 532 | dominated | |
| HIVE – 1 dose | 7.4 |  | 25.255 | 540 | dominated | |
| HR-HIVE – Extended | 7.1 |  | 25.284 | 544 | 381^†^ | |
| HIVE – 2 doses | 7.1 |  | 25.294 | 558 | dominated | |
| HIVE – Extended | 6.3 |  | 25.369 | 587 | 501^‡^ | |
| **South Africa [CET: ICER < $1,200/YLS (20% GDP per capita), ICER < $3,001 (50% GDP per capita)]** | | | | | |  |
| ***bNAb major toxicity one-time probability: 0%*** | | |  |  |  |  |
| Standard of care | 2.8 |  | 28.274 | 249 | *Reference* | |
| HR-HIVE – 1 dose | 2.5 |  | 28.325 | 235 | cost-saving | |
| HR-HIVE – 2 doses | 2.4 |  | 28.331 | 238 | dominated | |
| HR-HIVE – Extended | 2.4 |  | 28.337 | 240 | 377 | |
| HIVE – 1 dose | 2.3 |  | 28.355 | 254 | dominated | |
| HIVE – 2 doses | 2.1 |  | 28.386 | 267 | dominated | |
| HIVE – Extended | 1.9 |  | 28.421 | 279 | 464^†,‡^ | |
| ***bNAb major toxicity one-time probability: 5%*** | | |  |  |  |  |
| Standard of care | 2.8 |  | 28.274 | 249 | *Reference* | |
| HR-HIVE – 1 dose | 2.5 |  | 28.325 | 235 | cost-saving | |
| HR-HIVE – 2 doses | 2.4 |  | 28.331 | 238 | dominated | |
| HR-HIVE – Extended | 2.4 |  | 28.336 | 239 | 363 | |
| HIVE – 1 dose | 2.3 |  | 28.355 | 255 | dominated | |
| HIVE – 2 doses | 2.1 |  | 28.384 | 268 | dominated | |
| HIVE – Extended | 1.9 |  | 28.417 | 278 | 472^†,‡^ | |
| **Zimbabwe [CET: ICER < $293/YLS (20% GDP per capita), ICER < $732 (50% GDP per capita)]** | | | | | |  |
| ***bNAb major toxicity one-time probability: 0%*** | | |  |  |  |  |
| Standard of care | 6.3 |  | 27.158 | 411 | *Reference* | |
| HR-HIVE – 1 dose | 5.7 |  | 27.260 | 390 | cost-saving^†^ | |
| HR-HIVE – 2 doses | 5.6 |  | 27.275 | 396 | 367 | |
| HIVE – 1 dose | 5.4 |  | 27.298 | 414 | dominated | |
| HR-HIVE – Extended | 5.3 |  | 27.304 | 407 | 389 | |
| HIVE – 2 doses | 5.1 |  | 27.353 | 440 | dominated | |
| HIVE – Extended | 4.1 |  | 27.484 | 489 | 455^‡^ | |
| ***bNAb major toxicity one-time probability: 5%*** | | |  |  |  |  |
| Standard of care | 6.3 |  | 27.158 | 411 | *Reference* | |
| HR-HIVE – 1 dose | 5.7 |  | 27.260 | 390 | cost-saving^†^ | |
| HR-HIVE – 2 doses | 5.6 |  | 27.274 | 395 | 362 | |
| HIVE – 1 dose | 5.4 |  | 27.298 | 414 | dominated | |
| HR-HIVE – Extended | 5.3 |  | 27.302 | 406 | 393 | |
| HIVE – 2 doses | 5.1 |  | 27.351 | 439 | dominated | |
| HIVE – Extended | 4.1 |  | 27.475 | 486 | 460^‡^ | |

The most cost-effective bNAb implementation strategy was the strategy that offered the greatest increase in overall population life expectancy while still having an ICER less than the cost-effectiveness threshold when compared to the next best performing, non-dominated strategy.

yr, year; ICER, incremental cost-effectiveness ratio; YLS, year of life saved; CET, cost-effectiveness threshold; HR-HIVE, high-risk HIV-exposed infants; HIVE, all HIV-exposed infants.

^†^ Indicates the preferred strategy at a cost-effectiveness threshold of 20% GDP per capita.

^‡^ Indicates the preferred strategy at a cost-effectiveness threshold of 50% GDP per capita.

# **Table A15. One-way sensitivity analysis: bNAb uptake**

| **Country/strategy** | **Clinical outcomes** |  | **Lifetime efficacy and costs** | | |  |
| --- | --- | --- | --- | --- | --- | --- |
|  | **Total cumulative HIV incidence (%)** |  | **Discounted life expectancy (yrs)** | **Discounted**  **costs ($)** | **ICER ($/YLS)** |  |
| **Côte d’Ivoire [CET: ICER < $455/YLS (20% GDP per capita), ICER < $1,138 (50% GDP per capita)]** | | | | | |  |
| ***Low uptake at all time points*** | | |  |  |  |  |
| Standard of care | 8.4 |  | 25.106 | 550 | *Reference* | |
| HR-HIVE – 1 dose | 7.6 |  | 25.223 | 526 | cost-saving | |
| HR-HIVE – 2 doses | 7.6 |  | 25.231 | 529 | 408 | |
| HIVE – 1 dose | 7.5 |  | 25.241 | 537 | dominated | |
| HR-HIVE – Extended | 7.4 |  | 25.250 | 537 | 420^†^ | |
| HIVE – 2 doses | 7.4 |  | 25.259 | 544 | dominated | |
| HIVE – Extended | 6.9 |  | 25.305 | 563 | 477^‡^ | |
| ***High uptake at all time points*** | | |  |  |  |  |
| Standard of care | 8.4 |  | 25.106 | 550 | *Reference* |  |
| HR-HIVE – 1 dose | 7.4 |  | 25.250 | 525 | cost-saving |  |
| HR-HIVE – 2 doses | 7.3 |  | 25.268 | 532 | 362 |  |
| HIVE – 1 dose | 7.3 |  | 25.275 | 545 | dominated |  |
| HR-HIVE – Extended | 6.9 |  | 25.311 | 548 | 388^†^ |  |
| HIVE – 2 doses | 7.0 |  | 25.315 | 563 | dominated |  |
| HIVE – Extended | 5.9 |  | 25.415 | 602 | 517^‡^ |  |
| **South Africa [CET: ICER < $1,200/YLS (20% GDP per capita), ICER < $3,001 (50% GDP per capita)]** | | | | | |  |
| ***Low uptake at all time points*** | | |  |  |  |  |
| Standard of care | 2.8 |  | 28.274 | 249 | *Reference* |  |
| HR-HIVE – 1 dose | 2.5 |  | 28.324 | 235 | cost-saving |  |
| HR-HIVE – 2 doses | 2.5 |  | 28.327 | 236 | dominated |  |
| HR-HIVE – Extended | 2.4 |  | 28.332 | 238 | 363 |  |
| HIVE – 1 dose | 2.3 |  | 28.353 | 252 | dominated |  |
| HIVE – 2 doses | 2.2 |  | 28.371 | 260 | dominated |  |
| HIVE – Extended | 2.0 |  | 28.398 | 269 | 471^†,‡^ |  |
| ***High uptake at all time points*** | | |  |  |  |  |
| Standard of care | 2.8 |  | 28.274 | 249 | *Reference* |  |
| HR-HIVE – 1 dose | 2.5 |  | 28.325 | 235 | cost-saving |  |
| HR-HIVE – 2 doses | 2.4 |  | 28.331 | 238 | dominated |  |
| HR-HIVE – Extended | 2.4 |  | 28.339 | 240 | 359 |  |
| HIVE – 1 dose | 2.3 |  | 28.356 | 255 | dominated |  |
| HIVE – 2 doses | 2.1 |  | 28.387 | 268 | dominated |  |
| HIVE – Extended | 1.8 |  | 28.431 | 282 | 458^†,‡^ |  |
| **Zimbabwe [CET: ICER < $293/YLS (20% GDP per capita), ICER < $732 (50% GDP per capita)]** | | | | | |  |
| ***Low uptake at all time points*** | | |  |  |  |  |
| Standard of care | 6.3 |  | 27.158 | 411 | *Reference* |  |
| HR-HIVE – 1 dose | 5.7 |  | 27.247 | 391 | cost-saving^†^ |  |
| HR-HIVE – 2 doses | 5.7 |  | 27.257 | 394 | 389 |  |
| HIVE – 1 dose | 5.6 |  | 27.278 | 406 | dominated |  |
| HR-HIVE – Extended | 5.5 |  | 27.279 | 404 | 420 |  |
| HIVE – 2 doses | 5.3 |  | 27.316 | 424 | dominated |  |
| HIVE – Extended | 4.5 |  | 27.419 | 464 | 432^‡^ |  |
| ***High uptake at all time points*** | | |  |  |  |  |
| Standard of care | 6.3 |  | 27.158 | 411 | *Reference* |  |
| HR-HIVE – 1 dose | 5.6 |  | 27.262 | 390 | cost-saving^†^ |  |
| HR-HIVE – 2 doses | 5.5 |  | 27.278 | 395 | 368 |  |
| HIVE – 1 dose | 5.4 |  | 27.303 | 415 | dominated |  |
| HR-HIVE – Extended | 5.3 |  | 27.312 | 409 | 389 |  |
| HIVE – 2 doses | 5.1 |  | 27.357 | 441 | dominated |  |
| HIVE – Extended | 3.9 |  | 27.509 | 497 | 448^‡^ |  |

The setting-specific base case uptake is specified in Table A2. The low and high uptake scenarios were modeled by assuming uptake was simultaneously at its lowest or highest plausible value as supported by published literature, respectively (Table A2). The most cost-effective bNAb implementation strategy was the strategy that offered the greatest increase in overall population life expectancy while still having an ICER less than the cost-effectiveness threshold when compared to the next best performing, non-dominated strategy.

yr, year; ICER: incremental cost-effectiveness ratio; YLS: year of life saved; CET: cost-effectiveness threshold; HR-HIVE: high-risk HIV-exposed infants; HIVE: all HIV-exposed infants.

^†^ Indicates the preferred strategy at a cost-effectiveness threshold of 20% GDP per capita.

^‡^ Indicates the preferred strategy at a cost-effectiveness threshold of 50% GDP per capita.

# **Table A16. One-way sensitivity analysis: proportion of mothers on antiretroviral therapy during pregnancy**

| **Country/strategy** | **Clinical outcomes** |  | **Lifetime efficacy and costs** | | |  |
| --- | --- | --- | --- | --- | --- | --- |
|  | **Total cumulative HIV incidence (%)** |  | **Discounted life expectancy (yrs)** | **Discounted**  **costs ($)** | **ICER ($/YLS)** |  |
| **Côte d’Ivoire [CET: ICER < $455/YLS (20% GDP per capita), ICER < $1,138 (50% GDP per capita)]** | | | | | |  |
| ***Proportion of mothers on ART during pregnancy: 60%*** | | |  |  |  |  |
| Standard of care | 11.7 |  | 24.265 | 710 | *Reference* | |
| HR-HIVE – 1 dose | 10.3 |  | 24.469 | 669 | cost-saving | |
| HIVE – 1 dose | 10.2 |  | 24.483 | 680 | dominated | |
| HR-HIVE – 2 doses | 10.1 |  | 24.499 | 676 | 225 | |
| HIVE – 2 doses | 9.9 |  | 24.528 | 695 | dominated | |
| HR-HIVE – Extended | 9.7 |  | 24.544 | 694 | 388^†^ | |
| HIVE – Extended | 9.1 |  | 24.605 | 724 | 497^‡^ | |
| ***Proportion of mothers on ART during pregnancy: 100%*** | | |  |  |  |  |
| Standard of care | 6.7 |  | 25.532 | 468 | *Reference* |  |
| HR-HIVE – 1 dose | 6.2 |  | 25.622 | 451 | cost-saving |  |
| HR-HIVE – 2 doses | 6.1 |  | 25.634 | 459 | dominated |  |
| HIVE – 1 dose | 6.0 |  | 25.645 | 468 | dominated |  |
| HR-HIVE – Extended | 5.8 |  | 25.662 | 469 | 447^†^ |  |
| HIVE – 2 doses | 5.7 |  | 25.684 | 490 | dominated |  |
| HIVE – Extended | 4.9 |  | 25.763 | 519 | 497^‡^ |  |
| **South Africa [CET: ICER < $1,200/YLS (20% GDP per capita), ICER < $3,001 (50% GDP per capita)]** | | | | | |  |
| ***Proportion of mothers on ART during pregnancy: 80%*** | | |  |  |  |  |
| Standard of care | 5.2 |  | 27.558 | 409 | *Reference* |  |
| HR-HIVE – 1 dose | 4.4 |  | 27.685 | 369 | cost-saving |  |
| HR-HIVE – 2 doses | 4.3 |  | 27.699 | 372 | 225 |  |
| HR-HIVE – Extended | 4.2 |  | 27.709 | 375 | 337 |  |
| HIVE – 1 dose | 4.2 |  | 27.710 | 384 | dominated |  |
| HIVE – 2 doses | 4.0 |  | 27.744 | 396 | dominated |  |
| HIVE – Extended | 3.8 |  | 27.777 | 407 | 464^†,‡^ |  |
| ***Proportion of mothers on ART during pregnancy: 100%*** | | |  |  |  |  |
| Standard of care | 2.4 |  | 28.381 | 225 | *Reference* |  |
| HR-HIVE – 1 dose | 2.2 |  | 28.420 | 215 | cost-saving |  |
| HR-HIVE – 2 doses | 2.2 |  | 28.425 | 217 | dominated |  |
| HR-HIVE – Extended | 2.1 |  | 28.431 | 219 | 404 |  |
| HIVE – 1 dose | 2.0 |  | 28.451 | 234 | dominated |  |
| HIVE – 2 doses | 1.8 |  | 28.481 | 247 | dominated |  |
| HIVE – Extended | 1.6 |  | 28.516 | 259 | 464^†,‡^ |  |
| **Zimbabwe [CET: ICER < $293/YLS (20% GDP per capita), ICER < $732 (50% GDP per capita)]** | | | | | |  |
| ***Proportion of mothers on ART during pregnancy: 60%*** | | |  |  |  |  |
| Standard of care | 9.9 |  | 26.088 | 607 | *Reference* |  |
| HR-HIVE – 1 dose | 8.6 |  | 26.300 | 561 | cost-saving |  |
| HIVE – 1 dose | 8.5 |  | 26.327 | 578 | dominated |  |
| HR-HIVE – 2 doses | 8.4 |  | 26.337 | 570 | 234^†^ |  |
| HIVE – 2 doses | 8.1 |  | 26.391 | 601 | dominated |  |
| HR-HIVE – Extended | 8.0 |  | 26.392 | 592 | 397 |  |
| HIVE – Extended | 7.1 |  | 26.518 | 649 | 455^‡^ |  |
| ***Proportion of mothers on ART during pregnancy: 100%*** | | |  |  |  |  |
| Standard of care | 5.3 |  | 27.448 | 357 | *Reference* |  |
| HR-HIVE – 1 dose | 4.9 |  | 27.519 | 343 | cost-saving^†^ |  |
| HR-HIVE – 2 doses | 4.8 |  | 27.529 | 348 | dominated |  |
| HR-HIVE – Extended | 4.6 |  | 27.552 | 357 | 425 |  |
| HIVE – 1 dose | 4.6 |  | 27.561 | 369 | dominated |  |
| HIVE – 2 doses | 4.3 |  | 27.613 | 396 | dominated |  |
| HIVE – Extended | 3.3 |  | 27.745 | 445 | 455^‡^ |  |

The most cost-effective bNAb implementation strategy was the strategy that offered the greatest increase in overall population life expectancy while still having an ICER less than the cost-effectiveness threshold when compared to the next best performing, non-dominated strategy.

yr, year; ICER, incremental cost-effectiveness ratio; YLS, year of life saved; CET, cost-effectiveness threshold; ART, antiretroviral therapy; HR-HIVE, high-risk HIV-exposed infants; HIVE, all HIV-exposed infants.

^†^ Indicates the preferred strategy at a cost-effectiveness threshold of 20% GDP per capita.

^‡^ Indicates the preferred strategy at a cost-effectiveness threshold of 50% GDP per capita.

# **Table A17. One-way sensitivity analysis:** **proportion of mothers with viral load <1,000 copies/mL at delivery**

| **Country/strategy** | **Clinical outcomes** |  | **Lifetime efficacy and costs** | | |  |
| --- | --- | --- | --- | --- | --- | --- |
|  | **Total cumulative HIV incidence (%)** |  | **Discounted life expectancy (yrs)** | **Discounted**  **costs ($)** | **ICER ($/YLS)** |  |
| **Côte d’Ivoire [CET: ICER < $455/YLS (20% GDP per capita), ICER < $1,138 (50% GDP per capita)]** | | | | | |  |
| ***Proportion with viral load <1,000 copies/mL: 65%*** | | |  |  |  |  |
| Standard of care | 8.7 |  | 25.056 | 568 | *Reference* | |
| HR-HIVE – 1 dose | 7.8 |  | 25.195 | 544 | cost-saving | |
| HIVE – 1 dose | 7.7 |  | 25.213 | 557 | dominated | |
| HR-HIVE – 2 doses | 7.6 |  | 25.217 | 552 | dominated | |
| HIVE – 2 doses | 7.4 |  | 25.253 | 575 | dominated | |
| HR-HIVE – Extended | 7.2 |  | 25.258 | 568 | 379^†^ | |
| HIVE – Extended | 6.6 |  | 25.332 | 605 | 497^‡^ | |
| ***Proportion with viral load <1,000 copies/mL: 85%*** | | |  |  |  |  |
| Standard of care | 8.2 |  | 25.143 | 536 | *Reference* |  |
| HR-HIVE – 1 dose | 7.4 |  | 25.264 | 512 | cost-saving |  |
| HR-HIVE – 2 doses | 7.2 |  | 25.280 | 518 | dominated |  |
| HIVE – 1 dose | 7.2 |  | 25.287 | 528 | dominated |  |
| HR-HIVE – Extended | 7.0 |  | 25.308 | 529 | 383^†^ |  |
| HIVE – 2 doses | 6.9 |  | 25.327 | 548 | dominated |  |
| HIVE – Extended | 6.1 |  | 25.406 | 577 | 497^‡^ |  |
| **South Africa [CET: ICER < $1,200/YLS (20% GDP per capita), ICER < $3,001 (50% GDP per capita)]** | | | | | |  |
| ***Proportion with viral load <1,000 copies/mL: 75%*** | | |  |  |  |  |
| Standard of care | 3.2 |  | 28.209 | 279 | *Reference* |  |
| HR-HIVE – 1 dose | 2.8 |  | 28.277 | 262 | cost-saving |  |
| HR-HIVE – 2 doses | 2.7 |  | 28.287 | 266 | dominated |  |
| HR-HIVE – Extended | 2.6 |  | 28.296 | 269 | 379 |  |
| HIVE – 1 dose | 2.6 |  | 28.303 | 278 | dominated |  |
| HIVE – 2 doses | 2.4 |  | 28.334 | 291 | dominated |  |
| HIVE – Extended | 2.2 |  | 28.369 | 303 | 464^†,‡^ |  |
| ***Proportion with viral load <1,000 copies/mL: 95%*** | | |  |  |  |  |
| Standard of care | 2.5 |  | 28.322 | 227 | *Reference* |  |
| HR-HIVE – 1 dose | 2.3 |  | 28.361 | 215 | cost-saving |  |
| HR-HIVE – 2 doses | 2.2 |  | 28.364 | 217 | dominated |  |
| HR-HIVE – Extended | 2.2 |  | 28.368 | 218 | 349 |  |
| HIVE – 1 dose | 2.1 |  | 28.393 | 236 | dominated |  |
| HIVE – 2 doses | 1.9 |  | 28.424 | 249 | dominated |  |
| HIVE – Extended | 1.7 |  | 28.460 | 260 | 464^†,‡^ |  |
| **Zimbabwe [CET: ICER < $293/YLS (20% GDP per capita), ICER < $732 (50% GDP per capita)]** | | | | | |  |
| ***Proportion with viral load <1,000 copies/mL: 75%*** | | |  |  |  |  |
| Standard of care | 6.8 |  | 27.072 | 443 | *Reference* |  |
| HR-HIVE – 1 dose | 6.0 |  | 27.196 | 421 | cost-saving^†^ |  |
| HR-HIVE – 2 doses | 5.9 |  | 27.218 | 430 | dominated |  |
| HIVE – 1 dose | 5.8 |  | 27.228 | 441 | dominated |  |
| HR-HIVE – Extended | 5.5 |  | 27.265 | 448 | 401 |  |
| HIVE – 2 doses | 5.5 |  | 27.283 | 467 | dominated |  |
| HIVE – Extended | 4.5 |  | 27.413 | 516 | 455^‡^ |  |
| ***Proportion with viral load <1,000 copies/mL: 95%*** | | |  |  |  |  |
| Standard of care | 6.1 |  | 27.180 | 402 | *Reference* |  |
| HR-HIVE – 1 dose | 5.6 |  | 27.276 | 382 | cost-saving^†^ |  |
| HR-HIVE – 2 doses | 5.5 |  | 27.289 | 387 | 348 |  |
| HR-HIVE – Extended | 5.3 |  | 27.315 | 397 | 397 |  |
| HIVE – 1 dose | 5.3 |  | 27.316 | 407 | dominated |  |
| HIVE – 2 doses | 5.0 |  | 27.371 | 433 | dominated |  |
| HIVE – Extended | 4.0 |  | 27.502 | 482 | 455^‡^ |  |

The most cost-effective bNAb implementation strategy was the strategy that offered the greatest increase in overall population life expectancy while still having an ICER less than the cost-effectiveness threshold when compared to the next best performing, non-dominated strategy.

yr, year; ICER, incremental cost-effectiveness ratio; YLS, year of life saved; CET, cost-effectiveness threshold; HR-HIVE, high-risk HIV-exposed infants; HIVE, all HIV-exposed infants.

^†^ Indicates the preferred strategy at a cost-effectiveness threshold of 20% GDP per capita.

^b^ Indicates the preferred strategy at a cost-effectiveness threshold of 50% GDP per capita.

# **Table A18. One-way sensitivity analysis: perinatal vertical transmission risk**

| **Country/strategy** | **Clinical outcomes** |  | **Lifetime efficacy and costs** | | |  |
| --- | --- | --- | --- | --- | --- | --- |
|  | **Total cumulative HIV incidence (%)** |  | **Discounted life expectancy (yrs)** | **Discounted**  **costs ($)** | **ICER ($/YLS)** |  |
| **Côte d’Ivoire [CET: ICER < $455/YLS (20% GDP per capita), ICER < $1,138 (50% GDP per capita)]** | | | | | |  |
| ***Perinatal vertical transmission risk: 0.5x base case*** | | |  |  |  |  |
| Standard of care | 6.5 |  | 25.407 | 455 | *Reference* | |
| HR-HIVE – 1 dose | 5.9 |  | 25.499 | 441 | cost-saving | |
| HIVE – 1 dose | 5.7 |  | 25.516 | 458 | dominated | |
| HR-HIVE – 2 doses | 5.7 |  | 25.518 | 448 | 351 | |
| HR-HIVE – Extended | 5.4 |  | 25.553 | 461 | 372^†^ | |
| HIVE – 2 doses | 5.4 |  | 25.557 | 476 | dominated | |
| HIVE – Extended | 4.6 |  | 25.638 | 506 | 526^‡^ | |
| ***Perinatal vertical transmission risk: 2.0x base case*** | | |  |  |  |  |
| Standard of care | 12.3 |  | 24.504 | 741 | *Reference* |  |
| HR-HIVE – 1 dose | 10.9 |  | 24.707 | 693 | cost-saving |  |
| HR-HIVE – 2 doses | 10.8 |  | 24.723 | 700 | dominated |  |
| HIVE – 1 dose | 10.8 |  | 24.733 | 706 | dominated |  |
| HR-HIVE – Extended | 10.5 |  | 24.753 | 712 | 414 |  |
| HIVE – 2 doses | 10.5 |  | 24.771 | 724 | dominated |  |
| HIVE – Extended | 9.7 |  | 24.847 | 753 | 445^†,‡^ |  |
| **South Africa [CET: ICER < $1,200/YLS (20% GDP per capita), ICER < $3,001 (50% GDP per capita)]** | | | | | |  |
| ***Perinatal vertical transmission risk: 0.5x base case*** | | |  |  |  |  |
| Standard of care | 2.0 |  | 28.405 | 192 | *Reference* |  |
| HR-HIVE – 1 dose | 1.8 |  | 28.438 | 185 | cost-saving |  |
| HR-HIVE – 2 doses | 1.7 |  | 28.444 | 188 | dominated |  |
| HR-HIVE – Extended | 1.7 |  | 28.450 | 190 | 381 |  |
| HIVE – 1 dose | 1.6 |  | 28.461 | 207 | dominated |  |
| HIVE – 2 doses | 1.5 |  | 28.492 | 220 | dominated |  |
| HIVE – Extended | 1.2 |  | 28.527 | 232 | 547^†,‡^ |  |
| ***Perinatal vertical transmission risk: 2.0x base case*** | | |  |  |  |  |
| Standard of care | 4.4 |  | 28.012 | 363 | *Reference* |  |
| HR-HIVE – 1 dose | 3.9 |  | 28.099 | 333 | cost-saving |  |
| HR-HIVE – 2 doses | 3.8 |  | 28.105 | 336 | dominated |  |
| HR-HIVE – Extended | 3.8 |  | 28.110 | 338 | dominated |  |
| HIVE – 1 dose | 3.6 |  | 28.142 | 347 | 320 |  |
| HIVE – 2 doses | 3.4 |  | 28.172 | 360 | dominated |  |
| HIVE – Extended | 3.2 |  | 28.207 | 371 | 367^†,‡^ |  |
| **Zimbabwe [CET: ICER < $293/YLS (20% GDP per capita), ICER < $732 (50% GDP per capita)]** | | | | | |  |
| ***Perinatal vertical transmission risk: 0.5x base case*** | | |  |  |  |  |
| Standard of care | 5.0 |  | 27.366 | 342 | *Reference* |  |
| HR-HIVE – 1 dose | 4.6 |  | 27.440 | 330 | cost-saving^†^ |  |
| HR-HIVE – 2 doses | 4.5 |  | 27.456 | 336 | 358 |  |
| HIVE – 1 dose | 4.4 |  | 27.473 | 357 | dominated |  |
| HR-HIVE – Extended | 4.3 |  | 27.487 | 348 | 380 |  |
| HIVE – 2 doses | 4.1 |  | 27.528 | 383 | dominated |  |
| HIVE – Extended | 3.0 |  | 27.660 | 432 | 483^‡^ |  |
| ***Perinatal vertical transmission risk: 2.0x base case*** | | |  |  |  |  |
| Standard of care | 8.7 |  | 26.741 | 547 | *Reference* |  |
| HR-HIVE – 1 dose | 7.8 |  | 26.899 | 508 | cost-saving^†^ |  |
| HR-HIVE – 2 doses | 7.7 |  | 26.913 | 514 | dominated |  |
| HR-HIVE – Extended | 7.5 |  | 26.940 | 525 | 409 |  |
| HIVE – 1 dose | 7.5 |  | 26.948 | 529 | dominated |  |
| HIVE – 2 doses | 7.2 |  | 27.002 | 555 | dominated |  |
| HIVE – Extended | 6.2 |  | 27.129 | 603 | 411^‡^ |  |

The most cost-effective bNAb implementation strategy was the strategy that offered the greatest increase in overall population life expectancy while still having an ICER less than the cost-effectiveness threshold when compared to the next best performing, non-dominated strategy.

yr, year; ICER, incremental cost-effectiveness ratio; YLS, year of life saved; CET, cost-effectiveness threshold; HR-HIVE, high-risk HIV-exposed infants; HIVE, all HIV-exposed infants.

^†^ Indicates the preferred strategy at a cost-effectiveness threshold of 20% GDP per capita.

^‡^ Indicates the preferred strategy at a cost-effectiveness threshold of 50% GDP per capita.

# **Table A19. One-way sensitivity analysis: breastfeeding duration**

| **Country/strategy** | **Clinical outcomes** |  | **Lifetime efficacy and costs** | | |
| --- | --- | --- | --- | --- | --- |
|  | **Total cumulative HIV incidence (%)** |  | **Discounted life expectancy (yrs)** | **Discounted**  **costs ($)** | **ICER ($/YLS)** |
| **Côte d’Ivoire [CET: ICER < $455/YLS (20% GDP per capita), ICER < $1,138 (50% GDP per capita)]** | | | | | |
| ***Mean breastfeeding duration: 8 months*** | | |  |  |  |
| Standard of care | 6.7 |  | 25.280 | 425 | *Reference* |
| HR-HIVE – 1 dose | 5.9 |  | 25.402 | 399 | cost-saving |
| HR-HIVE – 2 doses | 5.8 |  | 25.417 | 405 | 414 |
| HIVE – 1 dose | 5.8 |  | 25.419 | 412 | dominated |
| HR-HIVE – Extended | 5.6 |  | 25.434 | 413 | 437^†^ |
| HIVE – 2 doses | 5.6 |  | 25.450 | 428 | dominated |
| HIVE – Extended | 5.2 |  | 25.490 | 444 | 559^‡^ |
| ***Mean breastfeeding duration: 24 months*** | | |  |  |  |
| Standard of care | 11.1 |  | 24.862 | 755 | *Reference* |
| HR-HIVE – 1 dose | 10.2 |  | 24.990 | 733 | cost-saving |
| HR-HIVE – 2 doses | 10.1 |  | 25.012 | 740 | 352 |
| HIVE – 1 dose | 10.1 |  | 25.013 | 749 | dominated |
| HIVE – 2 doses | 9.8 |  | 25.056 | 768 | dominated |
| HR-HIVE – Extended | 9.4 |  | 25.071 | 766 | 440^†^ |
| HIVE – Extended | 8.2 |  | 25.202 | 829 | 477^‡^ |
| **South Africa [CET: ICER < $1,200/YLS (20% GDP per capita), ICER < $3,001 (50% GDP per capita)]** | | | | | |
| ***Mean breastfeeding duration: 2 months*** | | |  |  |  |
| Standard of care | 2.2 |  | 28.362 | 211 | *Reference* |
| HR-HIVE – 1 dose | 1.9 |  | 28.410 | 196 | cost-saving |
| HR-HIVE – 2 doses | 1.9 |  | 28.413 | 198 | dominated |
| HR-HIVE – Extended | 1.9 |  | 28.415 | 199 | 426 |
| HIVE – 1 dose | 1.8 |  | 28.436 | 208 | dominated |
| HIVE – 2 doses | 1.7 |  | 28.453 | 217 | dominated |
| HIVE – Extended | 1.6 |  | 28.467 | 222 | 458^†,‡^ |
| ***Mean breastfeeding duration: 18 months*** | | |  |  |  |
| Standard of care | 5.1 |  | 27.920 | 413 | *Reference* |
| HR-HIVE – 1 dose | 4.8 |  | 27.973 | 400 | cost-saving |
| HR-HIVE – 2 doses | 4.7 |  | 27.982 | 404 | dominated |
| HR-HIVE – Extended | 4.6 |  | 28.011 | 425 | dominated |
| HIVE – 1 dose | 4.5 |  | 28.011 | 412 | 295 |
| HIVE – 2 doses | 4.3 |  | 28.059 | 444 | dominated |
| HIVE – Extended | 3.3 |  | 28.217 | 487 | 366^†,‡^ |
| **Zimbabwe [CET: ICER < $293/YLS (20% GDP per capita), ICER < $732 (50% GDP per capita)]** | | | | | |
| ***Mean breastfeeding duration: 8 months*** | | |  |  |  |
| Standard of care | 4.9 |  | 27.337 | 323 | *Reference* |
| HR-HIVE – 1 dose | 4.4 |  | 27.434 | 302 | cost-saving^†^ |
| HR-HIVE – 2 doses | 4.3 |  | 27.446 | 307 | dominated |
| HR-HIVE – Extended | 4.2 |  | 27.461 | 313 | 413 |
| HIVE – 1 dose | 4.2 |  | 27.467 | 323 | dominated |
| HIVE – 2 doses | 3.9 |  | 27.509 | 344 | dominated |
| HIVE – Extended | 3.3 |  | 27.579 | 371 | 493^‡^ |
| ***Mean breastfeeding duration: 20 months*** | | |  |  |  |
| Standard of care | 8.2 |  | 26.917 | 538 | *Reference* |
| HR-HIVE – 1 dose | 7.6 |  | 27.019 | 519 | cost-saving^†^ |
| HR-HIVE – 2 doses | 7.5 |  | 27.035 | 524 | 356 |
| HIVE – 1 dose | 7.4 |  | 27.056 | 544 | dominated |
| HR-HIVE – Extended | 7.1 |  | 27.088 | 546 | 421 |
| HIVE – 2 doses | 7.0 |  | 27.120 | 572 | dominated |
| HIVE – Extended | 5.1 |  | 27.351 | 667 | 456^‡^ |

The most cost-effective bNAb implementation strategy was the strategy that offered the greatest increase in overall population life expectancy while still having an ICER less than the cost-effectiveness threshold when compared to the next best performing, non-dominated strategy.

yr, year; ICER, incremental cost-effectiveness ratio; YLS, year of life saved; CET, cost-effectiveness threshold; HR-HIVE, high-risk HIV-exposed infants; HIVE, all HIV-exposed infants.

^†^ Indicates the preferred strategy at a cost-effectiveness threshold of 20% GDP per capita.

^‡^ Indicates the preferred strategy at a cost-effectiveness threshold of 50% GDP per capita.

# **Table A20. One-way sensitivity analysis: postpartum vertical transmission risk**

| **Country/strategy** | **Clinical outcomes** |  | **Lifetime efficacy and costs** | | |
| --- | --- | --- | --- | --- | --- |
|  | **Total cumulative HIV incidence (%)** |  | **Discounted life expectancy (yrs)** | **Discounted**  **costs ($)** | **ICER ($/YLS)** |
| **Côte d’Ivoire [CET: ICER < $455/YLS (20% GDP per capita), ICER < $1,138 (50% GDP per capita)]** | | | | | |
| ***Postpartum vertical transmission risk: 0.5x base case*** | | | |  |  |
| Standard of care | 6.3 |  | 25.347 | 405 | *Reference* |
| HR-HIVE – 1 dose | 5.6 |  | 25.453 | 388 | cost-saving^†,‡^ |
| HR-HIVE – 2 doses | 5.5 |  | 25.462 | 400 | 1,273 |
| HIVE – 1 dose | 5.5 |  | 25.466 | 406 | dominated |
| HR-HIVE – Extended | 5.3 |  | 25.479 | 425 | dominated |
| HIVE – 2 doses | 5.4 |  | 25.487 | 434 | 1,339 |
| HIVE – Extended | 4.9 |  | 25.527 | 494 | 1,502 |
| ***Postpartum vertical transmission risk: 2.0x base case*** | | | |  |  |
| Standard of care | 12.4 |  | 24.643 | 828 | *Reference* |
| HR-HIVE – 1 dose | 11.3 |  | 24.811 | 788 | dominated |
| HIVE – 1 dose | 11.1 |  | 24.844 | 798 | dominated |
| HR-HIVE – 2 doses | 11.1 |  | 24.847 | 788 | dominated |
| HR-HIVE – Extended | 10.4 |  | 24.911 | 777 | dominated |
| HIVE – 2 doses | 10.5 |  | 24.922 | 800 | dominated |
| HIVE – Extended | 9.0 |  | 25.075 | 772 | cost-saving^†,‡^ |
| **South Africa [CET: ICER < $1,200/YLS (20% GDP per capita), ICER < $3,001 (50% GDP per capita)]** | | | | | |
| ***Postpartum vertical transmission risk: 0.5x base case*** | | |  |  |  |
| Standard of care | 2.2 |  | 28.367 | 212 | *Reference* |
| HR-HIVE – 1 dose | 2.0 |  | 28.411 | 201 | cost-saving |
| HR-HIVE – 2 doses | 1.9 |  | 28.415 | 204 | dominated |
| HR-HIVE – Extended | 1.9 |  | 28.417 | 208 | dominated |
| HIVE – 1 dose | 1.8 |  | 28.433 | 223 | 1,004 |
| HIVE – 2 doses | 1.7 |  | 28.450 | 241 | dominated |
| HIVE – Extended | 1.6 |  | 28.467 | 260 | 1,096^†,‡^ |
| ***Postpartum vertical transmission risk: 2.0x base case*** | | |  |  |  |
| Standard of care | 3.9 |  | 28.090 | 319 | *Reference* |
| HR-HIVE – 1 dose | 3.5 |  | 28.153 | 301 | dominated |
| HR-HIVE – 2 doses | 3.4 |  | 28.165 | 302 | dominated |
| HR-HIVE – Extended | 3.4 |  | 28.177 | 301 | cost-saving |
| HIVE – 1 dose | 3.2 |  | 28.201 | 314 | dominated |
| HIVE – 2 doses | 2.9 |  | 28.260 | 318 | dominated |
| HIVE – Extended | 2.5 |  | 28.328 | 315 | 91^†,‡^ |
| **Zimbabwe [CET: ICER < $293/YLS (20% GDP per capita), ICER < $732 (50% GDP per capita)]** | | | | | |
| ***Postpartum vertical transmission risk: 0.5x base case*** | | | |  |  |
| Standard of care | 4.5 |  | 27.414 | 299 | *Reference* |
| HR-HIVE – 1 dose | 4.0 |  | 27.497 | 284 | cost-saving^†,‡^ |
| HR-HIVE – 2 doses | 3.9 |  | 27.504 | 293 | 1,055 |
| HR-HIVE – Extended | 3.8 |  | 27.520 | 312 | dominated |
| HIVE – 1 dose | 3.8 |  | 27.523 | 313 | 1,116 |
| HIVE – 2 doses | 3.7 |  | 27.551 | 348 | 1,215 |
| HIVE – Extended | 3.1 |  | 27.618 | 431 | 1,246 |
| ***Postpartum vertical transmission risk: 2.0x base case*** | | | |  |  |
| Standard of care | 9.7 |  | 26.659 | 624 | *Reference* |
| HR-HIVE – 1 dose | 8.9 |  | 26.796 | 592 | dominated |
| HR-HIVE – 2 doses | 8.8 |  | 26.825 | 594 | dominated |
| HIVE – 1 dose | 8.6 |  | 26.860 | 610 | dominated |
| HR-HIVE – Extended | 8.3 |  | 26.883 | 591 | cost-saving |
| HIVE – 2 doses | 7.9 |  | 26.966 | 619 | dominated |
| HIVE – Extended | 5.9 |  | 27.219 | 603 | 36^†,‡^ |

The most cost-effective bNAb implementation strategy was the strategy that offered the greatest increase in overall population life expectancy while still having an ICER less than the cost-effectiveness threshold when compared to the next best performing, non-dominated strategy.

yr, year; ICER, incremental cost-effectiveness ratio; YLS, year of life saved; CET, cost-effectiveness threshold; HR-HIVE, high-risk HIV-exposed infants; HIVE, all HIV-exposed infants.

^†^ Indicates the preferred strategy at a cost-effectiveness threshold of 20% GDP per capita.

^‡^ Indicates the preferred strategy at a cost-effectiveness threshold of 50% GDP per capita.

# **Table A21. One-way sensitivity analysis: postpartum maternal retention in care**

| **Country/strategy** | **Clinical outcomes** |  | **Lifetime efficacy and costs** | | |
| --- | --- | --- | --- | --- | --- |
|  | **Total cumulative HIV incidence (%)** |  | **Discounted life expectancy (yrs)** | **Discounted**  **costs ($)** | **ICER ($/YLS)** |
| **Côte d’Ivoire [CET: ICER < $455/YLS (20% GDP per capita), ICER < $1,138 (50% GDP per capita)]** | | | | | |
| ***Postpartum maternal retention in care: 50% at all time points*** | | | |  |  |
| Standard of care | 10.5 |  | 24.887 | 702 | *Reference* |
| HR-HIVE – 1 dose | 9.7 |  | 25.013 | 678 | dominated |
| HIVE – 1 dose | 9.5 |  | 25.037 | 692 | dominated |
| HR-HIVE – 2 doses | 9.4 |  | 25.041 | 681 | dominated |
| HR-HIVE – Extended | 8.9 |  | 25.096 | 678 | cost-saving |
| HIVE – 2 doses | 9.0 |  | 25.097 | 701 | dominated |
| HIVE – Extended | 7.7 |  | 25.229 | 691 | 97^†,‡^ |
| ***Postpartum maternal retention in care: 100% at all time points*** | | | |  |  |
| Standard of care | 7.5 |  | 25.194 | 488 | *Reference* |
| HR-HIVE – 1 dose | 6.7 |  | 25.322 | 462 | cost-saving^†^ |
| HR-HIVE – 2 doses | 6.6 |  | 25.337 | 471 | 587 |
| HIVE – 1 dose | 6.6 |  | 25.341 | 478 | dominated |
| HR-HIVE – Extended | 6.3 |  | 25.362 | 491 | 772 |
| HIVE – 2 doses | 6.3 |  | 25.374 | 500 | dominated |
| HIVE – Extended | 5.7 |  | 25.433 | 547 | 795^‡^ |
| **South Africa [CET: ICER < $1,200/YLS (20% GDP per capita), ICER < $3,001 (50% GDP per capita)]** | | | | | |
| ***Postpartum maternal retention in care: 50% at all time points*** | | |  |  |  |
| Standard of care | 3.4 |  | 28.176 | 288 | *Reference* |
| HR-HIVE – 1 dose | 3.1 |  | 28.227 | 274 | cost-saving |
| HR-HIVE – 2 doses | 3.0 |  | 28.236 | 275 | dominated |
| HR-HIVE – Extended | 2.9 |  | 28.247 | 276 | 77 |
| HIVE – 1 dose | 2.9 |  | 28.261 | 291 | dominated |
| HIVE – 2 doses | 2.6 |  | 28.311 | 298 | dominated |
| HIVE – Extended | 2.2 |  | 28.370 | 299 | 190^†,‡^ |
| ***Postpartum maternal retention in care: 100% at all time points*** | | |  |  |  |
| Standard of care | 2.6 |  | 28.306 | 236 | *Reference* |
| HR-HIVE – 1 dose | 2.3 |  | 28.357 | 222 | cost-saving |
| HR-HIVE – 2 doses | 2.2 |  | 28.362 | 225 | 567 |
| HR-HIVE – Extended | 2.2 |  | 28.366 | 228 | 598 |
| HIVE – 1 dose | 2.1 |  | 28.386 | 241 | dominated |
| HIVE – 2 doses | 2.0 |  | 28.411 | 257 | dominated |
| HIVE – Extended | 1.8 |  | 28.438 | 272 | 611^†,‡^ |
| **Zimbabwe [CET: ICER < $293/YLS (20% GDP per capita), ICER < $732 (50% GDP per capita)]** | | | | | |
| ***Postpartum maternal retention in care: 50% at all time points*** | | | |  |  |
| Standard of care | 8.2 |  | 26.890 | 533 | *Reference* |
| HR-HIVE – 1 dose | 7.6 |  | 26.990 | 513 | cost-saving |
| HR-HIVE – 2 doses | 7.5 |  | 27.013 | 517 | dominated |
| HIVE – 1 dose | 7.4 |  | 27.037 | 536 | dominated |
| HR-HIVE – Extended | 7.1 |  | 27.063 | 518 | 68 |
| HIVE – 2 doses | 6.8 |  | 27.123 | 552 | dominated |
| HIVE – Extended | 5.1 |  | 27.341 | 555 | 133^†,‡^ |
| ***Postpartum maternal retention in care: 100% at all time points*** | | | |  |  |
| Standard of care | 5.5 |  | 27.256 | 363 | *Reference* |
| HR-HIVE – 1 dose | 4.9 |  | 27.358 | 343 | cost-saving^†^ |
| HR-HIVE – 2 doses | 4.8 |  | 27.371 | 349 | 503 |
| HR-HIVE – Extended | 4.7 |  | 27.393 | 365 | dominated |
| HIVE – 1 dose | 4.7 |  | 27.395 | 367 | dominated |
| HIVE – 2 doses | 4.4 |  | 27.440 | 396 | 691 |
| HIVE – Extended | 3.7 |  | 27.537 | 463 | 692^‡^ |

The most cost-effective bNAb implementation strategy was the strategy that offered the greatest increase in overall population life expectancy while still having an ICER less than the cost-effectiveness threshold when compared to the next best performing, non-dominated strategy.

yr, year; ICER, incremental cost-effectiveness ratio; YLS, year of life saved; CET, cost-effectiveness threshold; HR-HIVE, high-risk HIV-exposed infants; HIVE, all HIV-exposed infants.

^†^ Indicates the preferred strategy at a cost-effectiveness threshold of 20% GDP per capita.

^‡^ Indicates the preferred strategy at a cost-effectiveness threshold of 50% GDP per capita.

# **Table A22. One-way sensitivity analysis: postpartum maternal virologic suppression**

| **Country/strategy** | **Clinical outcomes** |  | **Lifetime efficacy and costs** | | |
| --- | --- | --- | --- | --- | --- |
|  | **Total cumulative HIV incidence (%)** |  | **Discounted life expectancy (yrs)** | **Discounted**  **costs ($)** | **ICER ($/YLS)** |
| **Côte d’Ivoire [CET: ICER < $455/YLS (20% GDP per capita), ICER < $1,138 (50% GDP per capita)]** | | | | | |
| ***Postpartum maternal virologic suppression: low suppression scenario*** | | | |  |  |
| Standard of care | 8.7 |  | 25.075 | 570 | *Reference* |
| HR-HIVE – 1 dose | 7.8 |  | 25.203 | 546 | cost-saving |
| HR-HIVE – 2 doses | 7.7 |  | 25.223 | 552 | 290 |
| HIVE – 1 dose | 7.7 |  | 25.224 | 561 | dominated |
| HR-HIVE – Extended | 7.3 |  | 25.259 | 563 | 312 |
| HIVE – 2 doses | 7.4 |  | 25.268 | 577 | dominated |
| HIVE – Extended | 6.5 |  | 25.354 | 603 | 420^†,‡^ |
| ***Postpartum maternal virologic suppression: high suppression scenario*** | | | |  |  |
| Standard of care | 8.1 |  | 25.139 | 528 | *Reference* |
| HR-HIVE – 1 dose | 7.3 |  | 25.267 | 504 | cost-saving |
| HR-HIVE – 2 doses | 7.1 |  | 25.283 | 511 | 450^†^ |
| HIVE – 1 dose | 7.1 |  | 25.286 | 519 | dominated |
| HR-HIVE – Extended | 6.8 |  | 25.315 | 526 | 476 |
| HIVE – 2 doses | 6.8 |  | 25.323 | 539 | dominated |
| HIVE – Extended | 6.1 |  | 25.396 | 574 | 596^‡^ |
| **South Africa [CET: ICER < $1,200/YLS (20% GDP per capita), ICER < $3,001 (50% GDP per capita)]** | | | | | |
| ***Postpartum maternal virologic suppression: low suppression scenario*** | | |  |  |  |
| Standard of care | 2.9 |  | 28.258 | 255 | *Reference* |
| HR-HIVE – 1 dose | 2.6 |  | 28.309 | 241 | cost-saving |
| HR-HIVE – 2 doses | 2.5 |  | 28.316 | 243 | dominated |
| HR-HIVE – Extended | 2.5 |  | 28.323 | 245 | 302 |
| HIVE – 1 dose | 2.4 |  | 28.341 | 260 | dominated |
| HIVE – 2 doses | 2.2 |  | 28.375 | 272 | dominated |
| HIVE – Extended | 1.9 |  | 28.413 | 281 | 404^†,‡^ |
| ***Postpartum maternal virologic suppression: high suppression scenario*** | | |  |  |  |
| Standard of care | 2.7 |  | 28.289 | 243 | *Reference* |
| HR-HIVE – 1 dose | 2.4 |  | 28.340 | 229 | cost-saving |
| HR-HIVE – 2 doses | 2.3 |  | 28.345 | 232 | dominated |
| HR-HIVE – Extended | 2.3 |  | 28.351 | 234 | 450 |
| HIVE – 1 dose | 2.2 |  | 28.369 | 248 | dominated |
| HIVE – 2 doses | 2.1 |  | 28.396 | 263 | dominated |
| HIVE – Extended | 1.9 |  | 28.428 | 276 | 534^†,‡^ |
| **Zimbabwe [CET: ICER < $293/YLS (20% GDP per capita), ICER < $732 (50% GDP per capita)]** | | | | | |
| ***Postpartum maternal virologic suppression: low suppression scenario*** | | | |  |  |
| Standard of care | 6.5 |  | 27.119 | 427 | *Reference* |
| HR-HIVE – 1 dose | 5.9 |  | 27.221 | 407 | cost-saving^†^ |
| HR-HIVE – 2 doses | 5.8 |  | 27.238 | 412 | 308 |
| HIVE – 1 dose | 5.7 |  | 27.261 | 431 | dominated |
| HR-HIVE – Extended | 5.6 |  | 27.270 | 422 | 322 |
| HIVE – 2 doses | 5.3 |  | 27.322 | 455 | dominated |
| HIVE – Extended | 4.2 |  | 27.463 | 497 | 390^‡^ |
| ***Postpartum maternal virologic suppression: high suppression scenario*** | | | |  |  |
| Standard of care | 6.0 |  | 27.197 | 393 | *Reference* |
| HR-HIVE – 1 dose | 5.4 |  | 27.298 | 373 | cost-saving^†^ |
| HR-HIVE – 2 doses | 5.3 |  | 27.313 | 379 | 432 |
| HIVE – 1 dose | 5.2 |  | 27.335 | 397 | dominated |
| HR-HIVE – Extended | 5.1 |  | 27.340 | 392 | 476 |
| HIVE – 2 doses | 4.9 |  | 27.385 | 426 | dominated |
| HIVE – Extended | 3.9 |  | 27.504 | 480 | 537^‡^ |

The most cost-effective bNAb implementation strategy was the strategy that offered the greatest increase in overall population life expectancy while still having an ICER less than the cost-effectiveness threshold when compared to the next best performing, non-dominated strategy.

yr, year; ICER, incremental cost-effectiveness ratio; YLS, year of life saved; CET, cost-effectiveness threshold; HR-HIVE, high-risk HIV-exposed infants; HIVE, all HIV-exposed infants.

^†^ Indicates the preferred strategy at a cost-effectiveness threshold of 20% GDP per capita.

^‡^ Indicates the preferred strategy at a cost-effectiveness threshold of 50% GDP per capita.

# **Table A23. One-way sensitivity analysis: antiretroviral therapy treatment cost**

| **Country/strategy** | **Clinical outcomes** |  | **Lifetime efficacy and costs** | | |
| --- | --- | --- | --- | --- | --- |
|  | **Total cumulative HIV incidence (%)** |  | **Discounted life expectancy (yrs)** | **Discounted**  **costs ($)** | **ICER ($/YLS)** |
| **Côte d’Ivoire [CET: ICER < $455/YLS (20% GDP per capita), ICER < $1,138 (50% GDP per capita)]** | | | | | |
| ***ART treatment cost: 0.5x base case*** | | |  |  |  |
| Standard of care | 8.4 |  | 25.106 | 469 | *Reference* |
| HR-HIVE – 1 dose | 7.6 |  | 25.234 | 451 | cost-saving^†^ |
| HR-HIVE – 2 doses | 7.4 |  | 25.252 | 460 | 490 |
| HIVE – 1 dose | 7.4 |  | 25.255 | 467 | dominated |
| HR-HIVE – Extended | 7.1 |  | 25.286 | 477 | 504 |
| HIVE – 2 doses | 7.1 |  | 25.295 | 489 | dominated |
| HIVE – Extended | 6.3 |  | 25.374 | 529 | 592^‡^ |
| ***ART treatment cost: 2.0x base case*** | | |  |  |  |
| Standard of care | 8.4 |  | 25.106 | 712 | *Reference* |
| HR-HIVE – 1 dose | 7.6 |  | 25.234 | 674 | cost-saving |
| HR-HIVE – 2 doses | 7.4 |  | 25.252 | 677 | dominated |
| HIVE – 1 dose | 7.4 |  | 25.255 | 686 | dominated |
| HR-HIVE – Extended | 7.1 |  | 25.286 | 682 | 161 |
| HIVE – 2 doses | 7.1 |  | 25.295 | 698 | dominated |
| HIVE – Extended | 6.3 |  | 25.374 | 708 | 293^†,‡^ |
| **South Africa [CET: ICER < $1,200/YLS (20% GDP per capita), ICER < $3,001 (50% GDP per capita)]** | | | | | |
| ***ART treatment cost: 0.5x base case*** | | |  |  |  |
| Standard of care | 2.8 |  | 28.274 | 228 | *Reference* |
| HR-HIVE – 1 dose | 2.5 |  | 28.325 | 216 | cost-saving |
| HR-HIVE – 2 doses | 2.4 |  | 28.331 | 219 | dominated |
| HR-HIVE – Extended | 2.4 |  | 28.337 | 221 | 408 |
| HIVE – 1 dose | 2.3 |  | 28.355 | 237 | dominated |
| HIVE – 2 doses | 2.1 |  | 28.386 | 251 | dominated |
| HIVE – Extended | 1.9 |  | 28.421 | 264 | 503^†,‡^ |
| ***ART treatment cost: 2.0x base case*** | | |  |  |  |
| Standard of care | 2.8 |  | 28.274 | 291 | *Reference* |
| HR-HIVE – 1 dose | 2.5 |  | 28.325 | 273 | cost-saving |
| HR-HIVE – 2 doses | 2.4 |  | 28.331 | 274 | dominated |
| HR-HIVE – Extended | 2.4 |  | 28.337 | 276 | 298 |
| HIVE – 1 dose | 2.3 |  | 28.355 | 289 | dominated |
| HIVE – 2 doses | 2.1 |  | 28.386 | 300 | dominated |
| HIVE – Extended | 1.9 |  | 28.421 | 309 | 387^†,‡^ |
| **Zimbabwe [CET: ICER < $293/YLS (20% GDP per capita), ICER < $732 (50% GDP per capita)]** | | | | | |
| ***ART treatment cost: 0.5x base case*** | | |  |  |  |
| Standard of care | 6.3 |  | 27.158 | 353 | *Reference* |
| HR-HIVE – 1 dose | 5.7 |  | 27.260 | 337 | cost-saving^†^ |
| HR-HIVE – 2 doses | 5.6 |  | 27.275 | 344 | 431 |
| HIVE – 1 dose | 5.4 |  | 27.298 | 364 | dominated |
| HR-HIVE – Extended | 5.3 |  | 27.304 | 358 | 472 |
| HIVE – 2 doses | 5.1 |  | 27.353 | 393 | dominated |
| HIVE – Extended | 4.1 |  | 27.484 | 451 | 523^‡^ |
| ***ART treatment cost: 2.0x base case*** | | |  |  |  |
| Standard of care | 6.3 |  | 27.158 | 525 | *Reference* |
| HR-HIVE – 1 dose | 5.7 |  | 27.260 | 495 | cost-saving |
| HR-HIVE – 2 doses | 5.6 |  | 27.275 | 499 | dominated |
| HIVE – 1 dose | 5.4 |  | 27.298 | 515 | dominated |
| HR-HIVE – Extended | 5.3 |  | 27.304 | 506 | 251^†^ |
| HIVE – 2 doses | 5.1 |  | 27.353 | 536 | dominated |
| HIVE – Extended | 4.1 |  | 27.484 | 563 | 320^‡^ |

The most cost-effective bNAb implementation strategy was the strategy that offered the greatest increase in overall population life expectancy while still having an ICER less than the cost-effectiveness threshold when compared to the next best performing, non-dominated strategy.

yr, year; ICER, incremental cost-effectiveness ratio; YLS, year of life saved; CET, cost-effectiveness threshold; ART, antiretroviral therapy; HR-HIVE, high-risk HIV-exposed infants; HIVE, all HIV-exposed infants*.*

^†^ Indicates the preferred strategy at a cost-effectiveness threshold of 20% GDP per capita.

^‡^ Indicates the preferred strategy at a cost-effectiveness threshold of 50% GDP per capita.

# **Table A24. One-way sensitivity analysis: infant oral prophylaxis adherence**

| **Country/strategy** | **Clinical outcomes** |  | **Lifetime efficacy and costs** | | |
| --- | --- | --- | --- | --- | --- |
|  | **Total cumulative HIV incidence (%)** |  | **Discounted life expectancy (yrs)** | **Discounted**  **costs ($)** | **ICER ($/YLS)** |
| **Côte d’Ivoire [CET: ICER < $455/YLS (20% GDP per capita), ICER < $1,138 (50% GDP per capita)]** | | | | | |
| ***Infant oral prophylaxis adherence: 65%*** | | |  |  |  |
| Standard of care | 8.7 |  | 25.060 | 562 | *Reference* |
| HR-HIVE – 1 dose | 7.7 |  | 25.204 | 530 | cost-saving |
| HR-HIVE – 2 doses | 7.6 |  | 25.225 | 538 | dominated |
| HIVE – 1 dose | 7.6 |  | 25.226 | 545 | dominated |
| HR-HIVE – Extended | 7.3 |  | 25.258 | 550 | 376^†^ |
| HIVE – 2 doses | 7.3 |  | 25.268 | 564 | dominated |
| HIVE – Extended | 6.5 |  | 25.348 | 593 | 481^‡^ |
| ***Infant oral prophylaxis adherence: 98%*** | | |  |  |  |
| Standard of care | 8.2 |  | 25.134 | 543 | *Reference* |
| HR-HIVE – 1 dose | 7.4 |  | 25.253 | 522 | cost-saving |
| HR-HIVE – 2 doses | 7.3 |  | 25.270 | 529 | dominated |
| HIVE – 1 dose | 7.3 |  | 25.272 | 537 | dominated |
| HR-HIVE – Extended | 7.0 |  | 25.303 | 542 | 406^†^ |
| HIVE – 2 doses | 7.0 |  | 25.310 | 557 | dominated |
| HIVE – Extended | 6.2 |  | 25.389 | 586 | 509^‡^ |
| **South Africa [CET: ICER < $1,200/YLS (20% GDP per capita), ICER < $3,001 (50% GDP per capita)]** | | | | | |
| ***Infant oral prophylaxis adherence: 65%*** | | |  |  |  |
| Standard of care | 2.8 |  | 28.262 | 251 | *Reference* |
| HR-HIVE – 1 dose | 2.5 |  | 28.318 | 235 | cost-saving |
| HR-HIVE – 2 doses | 2.5 |  | 28.324 | 238 | dominated |
| HR-HIVE – Extended | 2.4 |  | 28.330 | 240 | 381 |
| HIVE – 1 dose | 2.3 |  | 28.350 | 253 | dominated |
| HIVE – 2 doses | 2.1 |  | 28.382 | 267 | dominated |
| HIVE – Extended | 1.9 |  | 28.416 | 278 | 444^†,‡^ |
| ***Infant oral prophylaxis adherence: 98%*** | | |  |  |  |
| Standard of care | 2.7 |  | 28.281 | 248 | *Reference* |
| HR-HIVE – 1 dose | 2.5 |  | 28.329 | 235 | cost-saving |
| HR-HIVE – 2 doses | 2.4 |  | 28.334 | 237 | dominated |
| HR-HIVE – Extended | 2.4 |  | 28.341 | 239 | 383 |
| HIVE – 1 dose | 2.3 |  | 28.357 | 254 | dominated |
| HIVE – 2 doses | 2.1 |  | 28.388 | 267 | dominated |
| HIVE – Extended | 1.9 |  | 28.423 | 278 | 472^†,‡^ |
| **Zimbabwe [CET: ICER < $293/YLS (20% GDP per capita), ICER < $732 (50% GDP per capita)]** | | | | | |
| ***Infant oral prophylaxis adherence: 65%*** | | |  |  |  |
| Standard of care | 6.5 |  | 27.124 | 417 | *Reference* |
| HR-HIVE – 1 dose | 5.8 |  | 27.241 | 391 | cost-saving^†^ |
| HR-HIVE – 2 doses | 5.7 |  | 27.256 | 397 | 375 |
| HIVE – 1 dose | 5.5 |  | 27.281 | 416 | dominated |
| HR-HIVE – Extended | 5.4 |  | 27.285 | 409 | 389 |
| HIVE – 2 doses | 5.2 |  | 27.337 | 442 | dominated |
| HIVE – Extended | 4.2 |  | 27.466 | 489 | 446^‡^ |
| ***Infant oral prophylaxis adherence: 98%*** | | |  |  |  |
| Standard of care | 6.1 |  | 27.177 | 406 | *Reference* |
| HR-HIVE – 1 dose | 5.6 |  | 27.271 | 388 | cost-saving^†^ |
| HR-HIVE – 2 doses | 5.5 |  | 27.285 | 393 | dominated |
| HIVE – 1 dose | 5.4 |  | 27.308 | 413 | dominated |
| HR-HIVE – Extended | 5.3 |  | 27.315 | 405 | 390 |
| HIVE – 2 doses | 5.0 |  | 27.361 | 439 | dominated |
| HIVE – Extended | 4.0 |  | 27.493 | 488 | 468^‡^ |

The most cost-effective bNAb implementation strategy was the strategy that offered the greatest increase in overall population life expectancy while still having an ICER less than the cost-effectiveness threshold when compared to the next best performing, non-dominated strategy.

yr, year; ICER, incremental cost-effectiveness ratio; YLS, year of life saved; CET, cost-effectiveness threshold; HR-HIVE, high-risk HIV-exposed infants; HIVE, all HIV-exposed infants.

^†^ Indicates the preferred strategy at a cost-effectiveness threshold of 20% GDP per capita.

^‡^ Indicates the preferred strategy at a cost-effectiveness threshold of 50% GDP per capita.

# **Table A25. One-way sensitivity analysis: infant oral prophylaxis cost**

| **Country/strategy** | **Clinical outcomes** |  | **Lifetime efficacy and costs** | | |  |
| --- | --- | --- | --- | --- | --- | --- |
|  | **Total cumulative HIV incidence (%)** |  | **Discounted life expectancy (yrs)** | **Discounted**  **costs ($)** | **ICER ($/YLS)** |  |
| **Côte d’Ivoire [CET: ICER < $455/YLS (20% GDP per capita), ICER < $1,138 (50% GDP per capita)]** | | | | | |  |
| ***Infant oral prophylaxis cost: 0.5x base case*** | | |  |  |  |  |
| Standard of care | 8.4 |  | 25.106 | 539 | *Reference* | |
| HR-HIVE – 1 dose | 7.6 |  | 25.234 | 514 | cost-saving | |
| HR-HIVE – 2 doses | 7.4 |  | 25.252 | 521 | dominated | |
| HIVE – 1 dose | 7.4 |  | 25.255 | 529 | dominated | |
| HR-HIVE – Extended | 7.1 |  | 25.286 | 534 | 389^†^ | |
| HIVE – 2 doses | 7.1 |  | 25.295 | 547 | dominated | |
| HIVE – Extended | 6.3 |  | 25.374 | 577 | 490^‡^ | |
| ***Infant oral prophylaxis: 2.0x base case*** | | |  |  |  |  |
| Standard of care | 8.4 |  | 25.106 | 572 | *Reference* |  |
| HR-HIVE – 1 dose | 7.6 |  | 25.234 | 547 | cost-saving |  |
| HR-HIVE – 2 doses | 7.4 |  | 25.252 | 554 | 367 |  |
| HIVE – 1 dose | 7.4 |  | 25.255 | 562 | dominated |  |
| HR-HIVE – Extended | 7.1 |  | 25.286 | 567 | 387^†^ |  |
| HIVE – 2 doses | 7.1 |  | 25.295 | 580 | dominated |  |
| HIVE – Extended | 6.3 |  | 25.374 | 611 | 497^‡^ |  |
| **South Africa [CET: ICER < $1,200/YLS (20% GDP per capita), ICER < $3,001 (50% GDP per capita)]** | | | | | |  |
| ***Infant oral prophylaxis cost: 0.5x base case*** | | |  |  |  |  |
| Standard of care | 2.8 |  | 28.274 | 244 | *Reference* |  |
| HR-HIVE – 1 dose | 2.5 |  | 28.325 | 231 | cost-saving |  |
| HR-HIVE – 2 doses | 2.4 |  | 28.331 | 233 | dominated |  |
| HR-HIVE – Extended | 2.4 |  | 28.337 | 235 | 377 |  |
| HIVE – 1 dose | 2.3 |  | 28.355 | 250 | dominated |  |
| HIVE – 2 doses | 2.1 |  | 28.386 | 263 | dominated |  |
| HIVE – Extended | 1.9 |  | 28.421 | 274 | 464^†,‡^ |  |
| ***Infant oral prophylaxis: 2.0x base case*** | | |  |  |  |  |
| Standard of care | 2.8 |  | 28.274 | 257 | *Reference* |  |
| HR-HIVE – 1 dose | 2.5 |  | 28.325 | 243 | cost-saving |  |
| HR-HIVE – 2 doses | 2.4 |  | 28.331 | 246 | dominated |  |
| HR-HIVE – Extended | 2.4 |  | 28.337 | 248 | 377 |  |
| HIVE – 1 dose | 2.3 |  | 28.355 | 262 | dominated |  |
| HIVE – 2 doses | 2.1 |  | 28.386 | 275 | dominated |  |
| HIVE – Extended | 1.9 |  | 28.421 | 287 | 464^†,‡^ |  |
| **Zimbabwe [CET: ICER < $293/YLS (20% GDP per capita), ICER < $732 (50% GDP per capita)]** | | | | | |  |
| ***Infant oral prophylaxis cost: 0.5x base case*** | | |  |  |  |  |
| Standard of care | 6.3 |  | 27.158 | 403 | *Reference* |  |
| HR-HIVE – 1 dose | 5.7 |  | 27.260 | 382 | cost-saving^†^ |  |
| HR-HIVE – 2 doses | 5.6 |  | 27.275 | 388 | 367 |  |
| HIVE – 1 dose | 5.4 |  | 27.298 | 406 | dominated |  |
| HR-HIVE – Extended | 5.3 |  | 27.304 | 400 | 397 |  |
| HIVE – 2 doses | 5.1 |  | 27.353 | 433 | dominated |  |
| HIVE – Extended | 4.1 |  | 27.484 | 481 | 455^‡^ |  |
| ***Infant oral prophylaxis cost: 2.0x base case*** | | |  |  |  |  |
| Standard of care | 6.3 |  | 27.158 | 426 | *Reference* |  |
| HR-HIVE – 1 dose | 5.7 |  | 27.260 | 405 | cost-saving^†^ |  |
| HR-HIVE – 2 doses | 5.6 |  | 27.275 | 411 | 367 |  |
| HIVE – 1 dose | 5.4 |  | 27.298 | 429 | dominated |  |
| HR-HIVE – Extended | 5.3 |  | 27.304 | 423 | 397 |  |
| HIVE – 2 doses | 5.1 |  | 27.353 | 455 | dominated |  |
| HIVE – Extended | 4.1 |  | 27.484 | 504 | 455^‡^ |  |

The most cost-effective bNAb implementation strategy was the strategy that offered the greatest increase in overall population life expectancy while still having an ICER less than the cost-effectiveness threshold when compared to the next best performing, non-dominated strategy.

yr, year; ICER, incremental cost-effectiveness ratio; YLS, year of life saved; CET, cost-effectiveness threshold; HR-HIVE, high-risk HIV-exposed infants; HIVE, all HIV-exposed infants.

^†^ Indicates the preferred strategy at a cost-effectiveness threshold of 20% GDP per capita.

^‡^ Indicates the preferred strategy at a cost-effectiveness threshold of 50% GDP per capita.

# **Table A26. One-way sensitivity analysis: infant oral prophylaxis efficacy**

| **Country/strategy** | **Clinical outcomes** |  | **Lifetime efficacy and costs** | | |
| --- | --- | --- | --- | --- | --- |
|  | **Total cumulative HIV incidence (%)** |  | **Discounted life expectancy (yrs)** | **Discounted**  **costs ($)** | **ICER ($/YLS)** |
| **Côte d’Ivoire [CET: ICER < $455/YLS (20% GDP per capita), ICER < $1,138 (50% GDP per capita)]** | | | | | |
| ***Infant oral prophylaxis efficacy: 30%*** | | |  |  |  |
| Standard of care | 9.1 |  | 24.999 | 591 | *Reference* |
| HR-HIVE – 1 dose | 8.0 |  | 25.168 | 551 | cost-saving |
| HR-HIVE – 2 doses | 7.9 |  | 25.186 | 558 | dominated |
| HIVE – 1 dose | 7.9 |  | 25.191 | 564 | dominated |
| HR-HIVE – Extended | 7.5 |  | 25.220 | 571 | 385^†^ |
| HIVE – 2 doses | 7.6 |  | 25.231 | 583 | dominated |
| HIVE – Extended | 6.7 |  | 25.310 | 613 | 464^‡^ |
| ***Infant oral prophylaxis efficacy: 95%*** | | |  |  |  |
| Standard of care | 7.9 |  | 25.171 | 526 | *Reference* |
| HR-HIVE – 1 dose | 7.3 |  | 25.274 | 510 | cost-saving |
| HR-HIVE – 2 doses | 7.1 |  | 25.292 | 517 | 368 |
| HIVE – 1 dose | 7.1 |  | 25.293 | 526 | dominated |
| HR-HIVE – Extended | 6.8 |  | 25.326 | 530 | 385^†^ |
| HIVE – 2 doses | 6.8 |  | 25.333 | 544 | dominated |
| HIVE – Extended | 6.0 |  | 25.413 | 574 | 506^‡^ |
| **South Africa [CET: ICER < $1,200/YLS (20% GDP per capita), ICER < $3,001 (50% GDP per capita)]** | | | | | |
| ***Infant oral prophylaxis efficacy: 30%*** | | |  |  |  |
| Standard of care | 2.9 |  | 28.246 | 259 | *Reference* |
| HR-HIVE – 1 dose | 2.6 |  | 28.309 | 241 | cost-saving |
| HR-HIVE – 2 doses | 2.5 |  | 28.315 | 243 | dominated |
| HR-HIVE – Extended | 2.5 |  | 28.321 | 245 | 379 |
| HIVE – 1 dose | 2.4 |  | 28.343 | 258 | dominated |
| HIVE – 2 doses | 2.2 |  | 28.374 | 272 | dominated |
| HIVE – Extended | 2.0 |  | 28.408 | 283 | 426^†,‡^ |
| ***Infant oral prophylaxis efficacy: 95%*** | | |  |  |  |
| Standard of care | 2.7 |  | 28.290 | 243 | *Reference* |
| HR-HIVE – 1 dose | 2.4 |  | 28.334 | 232 | cost-saving |
| HR-HIVE – 2 doses | 2.4 |  | 28.340 | 234 | dominated |
| HR-HIVE – Extended | 2.3 |  | 28.346 | 236 | 375 |
| HIVE – 1 dose | 2.3 |  | 28.362 | 251 | dominated |
| HIVE – 2 doses | 2.1 |  | 28.393 | 265 | dominated |
| HIVE – Extended | 1.9 |  | 28.428 | 276 | 486^†,‡^ |
| **Zimbabwe [CET: ICER < $293/YLS (20% GDP per capita), ICER < $732 (50% GDP per capita)]** | | | | | |
| ***Infant oral prophylaxis efficacy: 30%*** | | |  |  |  |
| Standard of care | 6.7 |  | 27.078 | 435 | *Reference* |
| HR-HIVE – 1 dose | 5.9 |  | 27.215 | 404 | cost-saving^†^ |
| HR-HIVE – 2 doses | 5.8 |  | 27.230 | 410 | 388 |
| HIVE – 1 dose | 5.7 |  | 27.258 | 426 | dominated |
| HR-HIVE – Extended | 5.6 |  | 27.260 | 421 | 391 |
| HIVE – 2 doses | 5.3 |  | 27.313 | 453 | dominated |
| HIVE – Extended | 4.3 |  | 27.443 | 501 | 435^‡^ |
| ***Infant oral prophylaxis efficacy: 95%*** | | |  |  |  |
| Standard of care | 6.0 |  | 27.205 | 395 | *Reference* |
| HR-HIVE – 1 dose | 5.5 |  | 27.286 | 381 | cost-saving^†^ |
| HR-HIVE – 2 doses | 5.4 |  | 27.302 | 387 | 363 |
| HIVE – 1 dose | 5.3 |  | 27.321 | 406 | dominated |
| HR-HIVE – Extended | 5.2 |  | 27.331 | 399 | 395 |
| HIVE – 2 doses | 5.0 |  | 27.377 | 433 | dominated |
| HIVE – Extended | 3.9 |  | 27.507 | 482 | 472^‡^ |

The most cost-effective bNAb implementation strategy was the strategy that offered the greatest increase in overall population life expectancy while still having an ICER less than the cost-effectiveness threshold when compared to the next best performing, non-dominated strategy.

yr, year; ICER, incremental cost-effectiveness ratio; YLS, year of life saved; CET, cost-effectiveness threshold; HR-HIVE, high-risk HIV-exposed infants; HIVE, all HIV-exposed infants.

^†^ Indicates the preferred strategy at a cost-effectiveness threshold of 20% GDP per capita.

^‡^ Indicates the preferred strategy at a cost-effectiveness threshold of 50% GDP per capita.

# **Table A27. One-way sensitivity analysis: infant oral prophylaxis major toxicity leading to discontinuation**

| **Country/strategy** | **Clinical outcomes** |  | **Lifetime efficacy and costs** | | |  |
| --- | --- | --- | --- | --- | --- | --- |
|  | **Total cumulative HIV incidence (%)** |  | **Discounted life expectancy (yrs)** | **Discounted**  **costs ($)** | **ICER ($/YLS)** |  |
| **Côte d’Ivoire [CET: ICER < $455/YLS (20% GDP per capita), ICER < $1,138 (50% GDP per capita)]** | | | | | |  |
| ***Infant oral prophylaxis major toxicity one-time probability: 0%*** | | | |  |  |  |
| Standard of care | 8.4 |  | 25.108 | 549 | *Reference* | |
| HR-HIVE – 1 dose | 7.5 |  | 25.236 | 525 | cost-saving | |
| HR-HIVE – 2 doses | 7.4 |  | 25.254 | 532 | dominated | |
| HIVE – 1 dose | 7.4 |  | 25.256 | 540 | dominated | |
| HR-HIVE – Extended | 7.1 |  | 25.288 | 544 | 381^†^ | |
| HIVE – 2 doses | 7.1 |  | 25.296 | 559 | dominated | |
| HIVE – Extended | 6.3 |  | 25.376 | 588 | 498^‡^ | |
| ***Infant oral prophylaxis major toxicity one-time probability: 2%*** | | | |  |  |  |
| Standard of care | 8.4 |  | 25.106 | 550 | *Reference* |  |
| HR-HIVE – 1 dose | 7.6 |  | 25.234 | 525 | cost-saving |  |
| HR-HIVE – 2 doses | 7.4 |  | 25.252 | 532 | 369 |  |
| HIVE – 1 dose | 7.4 |  | 25.255 | 540 | dominated |  |
| HR-HIVE – Extended | 7.1 |  | 25.286 | 545 | 387^†^ |  |
| HIVE – 2 doses | 7.1 |  | 25.295 | 559 | dominated |  |
| HIVE – Extended | 6.3 |  | 25.374 | 589 | 497^‡^ |  |
| **South Africa [CET: ICER < $1,200/YLS (20% GDP per capita), ICER < $3,001 (50% GDP per capita)]** | | | | | |  |
| ***Infant oral prophylaxis major toxicity one-time probability: 0%*** | | | |  |  |  |
| Standard of care | 2.8 |  | 28.274 | 249 | *Reference* |  |
| HR-HIVE – 1 dose | 2.5 |  | 28.325 | 235 | cost-saving |  |
| HR-HIVE – 2 doses | 2.4 |  | 28.331 | 238 | dominated |  |
| HR-HIVE – Extended | 2.4 |  | 28.337 | 239 | 361 |  |
| HIVE – 1 dose | 2.3 |  | 28.355 | 254 | dominated |  |
| HIVE – 2 doses | 2.1 |  | 28.386 | 267 | dominated |  |
| HIVE – Extended | 1.9 |  | 28.420 | 278 | 466^†,‡^ |  |
| ***Infant oral prophylaxis major toxicity one-time probability: 2%*** | | | |  |  |  |
| Standard of care | 2.8 |  | 28.274 | 249 | *Reference* |  |
| HR-HIVE – 1 dose | 2.5 |  | 28.325 | 235 | cost-saving |  |
| HR-HIVE – 2 doses | 2.4 |  | 28.331 | 238 | dominated |  |
| HR-HIVE – Extended | 2.4 |  | 28.337 | 240 | 376 |  |
| HIVE – 1 dose | 2.3 |  | 28.354 | 254 | dominated |  |
| HIVE – 2 doses | 2.1 |  | 28.386 | 268 | dominated |  |
| HIVE – Extended | 1.9 |  | 28.420 | 279 | 465^†,‡^ |  |
| **Zimbabwe [CET: ICER < $293/YLS (20% GDP per capita), ICER < $732 (50% GDP per capita)]** | | | | | |  |
| ***Infant oral prophylaxis major toxicity one-time probability: 0%*** | | | |  |  |  |
| Standard of care | 6.3 |  | 27.159 | 410 | *Reference* |  |
| HR-HIVE – 1 dose | 5.7 |  | 27.256 | 390 | cost-saving^†^ |  |
| HR-HIVE – 2 doses | 5.6 |  | 27.271 | 396 | 368 |  |
| HIVE – 1 dose | 5.5 |  | 27.292 | 411 | dominated |  |
| HR-HIVE – Extended | 5.4 |  | 27.301 | 407 | 394 |  |
| HIVE – 2 doses | 5.1 |  | 27.347 | 437 | dominated |  |
| HIVE – Extended | 4.1 |  | 27.477 | 486 | 444^‡^ |  |
| ***Infant oral prophylaxis major toxicity one-time probability: 2%*** | | | |  |  |  |
| Standard of care | 6.3 |  | 27.157 | 411 | *Reference* |  |
| HR-HIVE – 1 dose | 5.7 |  | 27.259 | 390 | cost-saving^†^ |  |
| HR-HIVE – 2 doses | 5.6 |  | 27.275 | 396 | 368 |  |
| HIVE – 1 dose | 5.4 |  | 27.298 | 414 | dominated |  |
| HR-HIVE – Extended | 5.3 |  | 27.304 | 407 | 396 |  |
| HIVE – 2 doses | 5.1 |  | 27.353 | 440 | dominated |  |
| HIVE – Extended | 4.1 |  | 27.483 | 489 | 455^‡^ |  |

The most cost-effective bNAb implementation strategy was the strategy that offered the greatest increase in overall population life expectancy while still having an ICER less than the cost-effectiveness threshold when compared to the next best performing, non-dominated strategy.

yr, year; ICER, incremental cost-effectiveness ratio; YLS, year of life saved; CET, cost-effectiveness threshold; HR-HIVE, high-risk HIV-exposed infants; HIVE, all HIV-exposed infants.

^†^ Indicates the preferred strategy at a cost-effectiveness threshold of 20% GDP per capita.

^‡^ Indicates the preferred strategy at a cost-effectiveness threshold of 50% GDP per capita.

# **Table A28. One-way sensitivity analysis: birth early infant diagnosis uptake**

| **Country/strategy** | **Clinical outcomes** |  | **Lifetime efficacy and costs** | | |  |
| --- | --- | --- | --- | --- | --- | --- |
|  | **Total cumulative HIV incidence (%)** |  | **Discounted life expectancy (yrs)** | **Discounted**  **costs ($)** | **ICER ($/YLS)** |  |
| **Côte d’Ivoire [CET: ICER < $455/YLS (20% GDP per capita), ICER < $1,138 (50% GDP per capita)]** | | | | | |  |
| ***Birth early infant diagnosis uptake: 0%*** | | |  |  |  |  |
| Standard of care | 8.4 |  | 25.106 | 550 | *Reference* | |
| HR-HIVE – 1 dose | 7.6 |  | 25.234 | 525 | cost-saving | |
| HR-HIVE – 2 doses | 7.4 |  | 25.252 | 532 | 367 | |
| HIVE – 1 dose | 7.4 |  | 25.255 | 540 | dominated | |
| HR-HIVE – Extended | 7.1 |  | 25.286 | 545 | 387^†^ | |
| HIVE – 2 doses | 7.1 |  | 25.295 | 559 | dominated | |
| HIVE – Extended | 6.3 |  | 25.374 | 589 | 497^‡^ | |
| ***Birth early infant diagnosis uptake: 50%*** | | |  |  |  |  |
| Standard of care | 8.4 |  | 25.125 | 575 | *Reference* |  |
| HR-HIVE – 1 dose | 7.6 |  | 25.250 | 549 | cost-saving |  |
| HR-HIVE – 2 doses | 7.4 |  | 25.269 | 555 | 318 |  |
| HIVE – 1 dose | 7.4 |  | 25.274 | 564 | dominated |  |
| HR-HIVE – Extended | 7.1 |  | 25.302 | 568 | 391^†^ |  |
| HIVE – 2 doses | 7.1 |  | 25.315 | 581 | dominated |  |
| HIVE – Extended | 6.3 |  | 25.393 | 611 | 469^‡^ |  |
| **South Africa [CET: ICER < $1,200/YLS (20% GDP per capita), ICER < $3,001 (50% GDP per capita)]** | | | | | |  |
| ***Birth early infant diagnosis uptake: 30%*** | | |  |  |  |  |
| Standard of care | 2.8 |  | 28.261 | 224 | *Reference* |  |
| HR-HIVE – 1 dose | 2.5 |  | 28.313 | 212 | cost-saving |  |
| HR-HIVE – 2 doses | 2.4 |  | 28.320 | 214 | dominated |  |
| HR-HIVE – Extended | 2.4 |  | 28.326 | 216 | 360 |  |
| HIVE – 1 dose | 2.3 |  | 28.345 | 231 | dominated |  |
| HIVE – 2 doses | 2.1 |  | 28.377 | 244 | dominated |  |
| HIVE – Extended | 1.9 |  | 28.411 | 256 | 466^†,‡^ |  |
| ***Birth early infant diagnosis uptake: 100%*** | | |  |  |  |  |
| Standard of care | 2.8 |  | 28.276 | 252 | *Reference* |  |
| HR-HIVE – 1 dose | 2.5 |  | 28.326 | 238 | cost-saving |  |
| HR-HIVE – 2 doses | 2.4 |  | 28.333 | 240 | dominated |  |
| HR-HIVE – Extended | 2.4 |  | 28.339 | 242 | 358 |  |
| HIVE – 1 dose | 2.3 |  | 28.356 | 256 | dominated |  |
| HIVE – 2 doses | 2.1 |  | 28.387 | 270 | dominated |  |
| HIVE – Extended | 1.9 |  | 28.421 | 281 | 470^†,‡^ |  |
| **Zimbabwe [CET: ICER < $293/YLS (20% GDP per capita), ICER < $732 (50% GDP per capita)]** | | | | | |  |
| ***Birth early infant diagnosis uptake: 0%*** | | |  |  |  |  |
| Standard of care | 6.3 |  | 27.155 | 406 | *Reference* |  |
| HR-HIVE – 1 dose | 5.7 |  | 27.257 | 386 | cost-saving^†^ |  |
| HR-HIVE – 2 doses | 5.6 |  | 27.272 | 392 | dominated |  |
| HIVE – 1 dose | 5.4 |  | 27.296 | 410 | dominated |  |
| HR-HIVE – Extended | 5.3 |  | 27.302 | 403 | 385 |  |
| HIVE – 2 doses | 5.1 |  | 27.351 | 436 | dominated |  |
| HIVE – Extended | 4.1 |  | 27.481 | 485 | 454^‡^ |  |
| ***Birth early infant diagnosis uptake: 50%*** | | |  |  |  |  |
| Standard of care | 6.3 |  | 27.173 | 430 | *Reference* |  |
| HR-HIVE – 1 dose | 5.7 |  | 27.272 | 408 | cost-saving^†^ |  |
| HR-HIVE – 2 doses | 5.6 |  | 27.287 | 414 | 348 |  |
| HIVE – 1 dose | 5.4 |  | 27.308 | 433 | dominated |  |
| HR-HIVE – Extended | 5.3 |  | 27.318 | 425 | 380 |  |
| HIVE – 2 doses | 5.1 |  | 27.364 | 458 | dominated |  |
| HIVE – Extended | 4.1 |  | 27.496 | 507 | 457^‡^ |  |

The most cost-effective bNAb implementation strategy was the strategy that offered the greatest increase in overall population life expectancy while still having an ICER less than the cost-effectiveness threshold when compared to the next best performing, non-dominated strategy.

yr, year; ICER, incremental cost-effectiveness ratio; YLS, year of life saved; CET, cost-effectiveness threshold; HR-HIVE, high-risk HIV-exposed infants; HIVE, all HIV-exposed infants.

^†^ Indicates the preferred strategy at a cost-effectiveness threshold of 20% GDP per capita.

^‡^ Indicates the preferred strategy at a cost-effectiveness threshold of 50% GDP per capita.

# **Table A29. One-way sensitivity analysis: six to eight week early infant diagnosis uptake**

| **Country/strategy** | **Clinical outcomes** |  | **Lifetime efficacy and costs** | | |  |
| --- | --- | --- | --- | --- | --- | --- |
|  | **Total cumulative HIV incidence (%)** |  | **Discounted life expectancy (yrs)** | **Discounted**  **costs ($)** | **ICER ($/YLS)** |  |
| **Côte d’Ivoire [CET: ICER < $455/YLS (20% GDP per capita), ICER < $1,138 (50% GDP per capita)]** | | | | | |  |
| ***6-8 week early infant diagnosis uptake: 40%*** | | |  |  |  |  |
| Standard of care | 8.4 |  | 25.084 | 530 | *Reference* | |
| HR-HIVE – 1 dose | 7.6 |  | 25.215 | 507 | cost-saving | |
| HR-HIVE – 2 doses | 7.4 |  | 25.233 | 514 | dominated | |
| HIVE – 1 dose | 7.4 |  | 25.237 | 522 | dominated | |
| HR-HIVE – Extended | 7.1 |  | 25.267 | 527 | 401^†^ | |
| HIVE – 2 doses | 7.1 |  | 25.275 | 541 | dominated | |
| HIVE – Extended | 6.3 |  | 25.355 | 572 | 501^‡^ | |
| ***6-8 week early infant diagnosis uptake: 75%*** | | |  |  |  |  |
| Standard of care | 8.4 |  | 25.111 | 553 | *Reference* |  |
| HR-HIVE – 1 dose | 7.6 |  | 25.238 | 529 | cost-saving |  |
| HR-HIVE – 2 doses | 7.4 |  | 25.257 | 535 | 358 |  |
| HIVE – 1 dose | 7.4 |  | 25.259 | 544 | dominated |  |
| HR-HIVE – Extended | 7.1 |  | 25.290 | 549 | 396^†^ |  |
| HIVE – 2 doses | 7.1 |  | 25.299 | 562 | dominated |  |
| HIVE – Extended | 6.3 |  | 25.378 | 592 | 496^‡^ |  |
| **South Africa [CET: ICER < $1,200/YLS (20% GDP per capita), ICER < $3,001 (50% GDP per capita)]** | | | | | |  |
| ***6-8 week early infant diagnosis uptake: 40%*** | | |  |  |  |  |
| Standard of care | 2.8 |  | 28.269 | 240 | *Reference* |  |
| HR-HIVE – 1 dose | 2.5 |  | 28.321 | 226 | cost-saving |  |
| HR-HIVE – 2 doses | 2.4 |  | 28.326 | 229 | dominated |  |
| HR-HIVE – Extended | 2.4 |  | 28.332 | 231 | 385 |  |
| HIVE – 1 dose | 2.3 |  | 28.351 | 246 | dominated |  |
| HIVE – 2 doses | 2.1 |  | 28.382 | 259 | dominated |  |
| HIVE – Extended | 1.9 |  | 28.417 | 271 | 470^†,‡^ |  |
| ***6-8 week early infant diagnosis uptake: 90%*** | | |  |  |  |  |
| Standard of care | 2.8 |  | 28.278 | 259 | *Reference* |  |
| HR-HIVE – 1 dose | 2.5 |  | 28.328 | 244 | cost-saving |  |
| HR-HIVE – 2 doses | 2.4 |  | 28.334 | 247 | dominated |  |
| HR-HIVE – Extended | 2.4 |  | 28.340 | 249 | 364 |  |
| HIVE – 1 dose | 2.3 |  | 28.358 | 263 | dominated |  |
| HIVE – 2 doses | 2.1 |  | 28.389 | 276 | dominated |  |
| HIVE – Extended | 1.9 |  | 28.423 | 287 | 459^†,‡^ |  |
| **Zimbabwe [CET: ICER < $293/YLS (20% GDP per capita), ICER < $732 (50% GDP per capita)]** | | | | | |  |
| ***6-8 week early infant diagnosis uptake: 20%*** | | |  |  |  |  |
| Standard of care | 6.3 |  | 27.140 | 390 | *Reference* |  |
| HR-HIVE – 1 dose | 5.7 |  | 27.245 | 371 | cost-saving^†^ |  |
| HR-HIVE – 2 doses | 5.6 |  | 27.260 | 377 | 369 |  |
| HIVE – 1 dose | 5.4 |  | 27.283 | 396 | dominated |  |
| HR-HIVE – Extended | 5.3 |  | 27.290 | 389 | 401 |  |
| HIVE – 2 doses | 5.1 |  | 27.339 | 422 | dominated |  |
| HIVE – Extended | 4.1 |  | 27.471 | 471 | 457^‡^ |  |
| ***6-8 week early infant diagnosis uptake: 100%*** | | |  |  |  |  |
| Standard of care | 6.3 |  | 27.183 | 438 | *Reference* |  |
| HR-HIVE – 1 dose | 5.7 |  | 27.279 | 415 | cost-saving^†^ |  |
| HR-HIVE – 2 doses | 5.6 |  | 27.296 | 420 | 344 |  |
| HIVE – 1 dose | 5.4 |  | 27.317 | 438 | dominated |  |
| HR-HIVE – Extended | 5.3 |  | 27.325 | 432 | 380 |  |
| HIVE – 2 doses | 5.1 |  | 27.371 | 464 | dominated |  |
| HIVE – Extended | 4.1 |  | 27.502 | 513 | 456^‡^ |  |

The most cost-effective bNAb implementation strategy was the strategy that offered the greatest increase in overall population life expectancy while still having an ICER less than the cost-effectiveness threshold when compared to the next best performing, non-dominated strategy.

yr, year; ICER, incremental cost-effectiveness ratio; YLS, year of life saved; CET, cost-effectiveness threshold; HR-HIVE, high-risk HIV-exposed infants; HIVE, all HIV-exposed infants.

^†^ Indicates the preferred strategy at a cost-effectiveness threshold of 20% GDP per capita.

^‡^ Indicates the preferred strategy at a cost-effectiveness threshold of 50% GDP per capita.

# **Table A30. One-way sensitivity analysis: six/nine month early infant diagnosis uptake**

| **Country/strategy** | **Clinical outcomes** |  | **Lifetime efficacy and costs** | | |  |
| --- | --- | --- | --- | --- | --- | --- |
|  | **Total cumulative HIV incidence (%)** |  | **Discounted life expectancy (yrs)** | **Discounted**  **costs ($)** | **ICER ($/YLS)** |  |
| **Côte d’Ivoire [CET: ICER < $455/YLS (20% GDP per capita), ICER < $1,138 (50% GDP per capita)]** | | | | | |  |
| ***9 month early infant diagnosis uptake: 0%*** | | |  |  |  |  |
| Standard of care | 8.4 |  | 25.100 | 539 | *Reference* | |
| HR-HIVE – 1 dose | 7.6 |  | 25.229 | 515 | cost-saving | |
| HR-HIVE – 2 doses | 7.4 |  | 25.247 | 522 | 386 | |
| HIVE – 1 dose | 7.4 |  | 25.248 | 530 | dominated | |
| HR-HIVE – Extended | 7.1 |  | 25.280 | 535 | 396^†^ | |
| HIVE – 2 doses | 7.1 |  | 25.290 | 549 | dominated | |
| HIVE – Extended | 6.3 |  | 25.370 | 579 | 489^‡^ | |
| ***9 month early infant diagnosis uptake: 50%*** | | |  |  |  |  |
| Standard of care | 8.4 |  | 25.114 | 561 | *Reference* |  |
| HR-HIVE – 1 dose | 7.6 |  | 25.241 | 535 | cost-saving |  |
| HR-HIVE – 2 doses | 7.4 |  | 25.258 | 543 | dominated |  |
| HIVE – 1 dose | 7.4 |  | 25.262 | 550 | dominated |  |
| HR-HIVE – Extended | 7.1 |  | 25.292 | 555 | 386^†^ |  |
| HIVE – 2 doses | 7.1 |  | 25.301 | 569 | dominated |  |
| HIVE – Extended | 6.3 |  | 25.379 | 597 | 489^‡^ |  |
| **South Africa [CET: ICER < $1,200/YLS (20% GDP per capita), ICER < $3,001 (50% GDP per capita)]** | | | | | |  |
| ***6 month early infant diagnosis uptake: 20%*** | | |  |  |  |  |
| Standard of care | 2.8 |  | 28.274 | 247 | *Reference* |  |
| HR-HIVE – 1 dose | 2.5 |  | 28.325 | 233 | cost-saving |  |
| HR-HIVE – 2 doses | 2.4 |  | 28.331 | 236 | dominated |  |
| HR-HIVE – Extended | 2.4 |  | 28.337 | 238 | 365 |  |
| HIVE – 1 dose | 2.3 |  | 28.355 | 253 | dominated |  |
| HIVE – 2 doses | 2.1 |  | 28.386 | 266 | dominated |  |
| HIVE – Extended | 1.9 |  | 28.421 | 277 | 463^†,‡^ |  |
| ***6 month early infant diagnosis uptake: 40%*** | | |  |  |  |  |
| Standard of care | 2.8 |  | 28.276 | 253 | *Reference* |  |
| HR-HIVE – 1 dose | 2.5 |  | 28.327 | 239 | cost-saving |  |
| HR-HIVE – 2 doses | 2.4 |  | 28.332 | 242 | dominated |  |
| HR-HIVE – Extended | 2.4 |  | 28.339 | 244 | 381 |  |
| HIVE – 1 dose | 2.3 |  | 28.358 | 259 | dominated |  |
| HIVE – 2 doses | 2.1 |  | 28.387 | 272 | dominated |  |
| HIVE – Extended | 1.9 |  | 28.422 | 283 | 464^†,‡^ |  |
| **Zimbabwe [CET: ICER < $293/YLS (20% GDP per capita), ICER < $732 (50% GDP per capita)]** | | | | | |  |
| ***9 month early infant diagnosis uptake: 20%*** | | |  |  |  |  |
| Standard of care | 6.3 |  | 27.157 | 408 | *Reference* |  |
| HR-HIVE – 1 dose | 5.7 |  | 27.259 | 387 | cost-saving^†^ |  |
| HR-HIVE – 2 doses | 5.6 |  | 27.274 | 393 | 386 |  |
| HIVE – 1 dose | 5.4 |  | 27.296 | 411 | dominated |  |
| HR-HIVE – Extended | 5.3 |  | 27.303 | 404 | 388 |  |
| HIVE – 2 doses | 5.1 |  | 27.352 | 438 | dominated |  |
| HIVE – Extended | 4.1 |  | 27.483 | 486 | 459^‡^ |  |
| ***9 month early infant diagnosis uptake: 40%*** | | |  |  |  |  |
| Standard of care | 6.3 |  | 27.160 | 416 | *Reference* |  |
| HR-HIVE – 1 dose | 5.7 |  | 27.262 | 396 | cost-saving^†^ |  |
| HR-HIVE – 2 doses | 5.6 |  | 27.277 | 401 | 366 |  |
| HIVE – 1 dose | 5.4 |  | 27.300 | 420 | dominated |  |
| HR-HIVE – Extended | 5.3 |  | 27.306 | 413 | 393 |  |
| HIVE – 2 doses | 5.1 |  | 27.355 | 446 | dominated |  |
| HIVE – Extended | 4.1 |  | 27.485 | 493 | 452^‡^ |  |

The most cost-effective bNAb implementation strategy was the strategy that offered the greatest increase in overall population life expectancy while still having an ICER less than the cost-effectiveness threshold when compared to the next best performing, non-dominated strategy.

yr, year; ICER, incremental cost-effectiveness ratio; YLS, year of life saved; CET, cost-effectiveness threshold; HR-HIVE, high-risk HIV-exposed infants; HIVE, all HIV-exposed infants.

^†^ Indicates the preferred strategy at a cost-effectiveness threshold of 20% GDP per capita.

^‡^ Indicates the preferred strategy at a cost-effectiveness threshold of 50% GDP per capita.

# **Table A31. One-way sensitivity analysis: 18 month early infant diagnosis uptake**

| **Country/strategy** | **Clinical outcomes** |  | **Lifetime efficacy and costs** | | |
| --- | --- | --- | --- | --- | --- |
|  | **Total cumulative HIV incidence (%)** |  | **Discounted life expectancy (yrs)** | **Discounted**  **costs ($)** | **ICER ($/YLS)** |
| **Côte d’Ivoire [CET: ICER < $455/YLS (20% GDP per capita), ICER < $1,138 (50% GDP per capita)]** | | | | | |
| ***18 month early infant diagnosis uptake: 0%*** | | |  |  |  |
| Standard of care | 8.4 |  | 25.092 | 541 | *Reference* |
| HR-HIVE – 1 dose | 7.6 |  | 25.221 | 517 | cost-saving |
| HR-HIVE – 2 doses | 7.4 |  | 25.240 | 523 | 358 |
| HIVE – 1 dose | 7.4 |  | 25.244 | 532 | dominated |
| HR-HIVE – Extended | 7.1 |  | 25.275 | 537 | 399^†^ |
| HIVE – 2 doses | 7.1 |  | 25.282 | 550 | dominated |
| HIVE – Extended | 6.3 |  | 25.366 | 582 | 493^‡^ |
| ***18 month early infant diagnosis uptake: 100%*** | | |  |  |  |
| Standard of care | 8.4 |  | 25.153 | 582 | *Reference* |
| HR-HIVE – 1 dose | 7.6 |  | 25.278 | 557 | cost-saving |
| HR-HIVE – 2 doses | 7.4 |  | 25.296 | 563 | dominated |
| HIVE – 1 dose | 7.4 |  | 25.299 | 571 | dominated |
| HR-HIVE – Extended | 7.1 |  | 25.323 | 573 | 369^†^ |
| HIVE – 2 doses | 7.1 |  | 25.339 | 589 | dominated |
| HIVE – Extended | 6.3 |  | 25.405 | 612 | 468^‡^ |
| **South Africa [CET: ICER < $1,200/YLS (20% GDP per capita), ICER < $3,001 (50% GDP per capita)]** | | | | | |
| ***18 month early infant diagnosis uptake: 0%*** | | |  |  |  |
| Standard of care | 2.8 |  | 28.271 | 247 | *Reference* |
| HR-HIVE – 1 dose | 2.5 |  | 28.322 | 233 | cost-saving |
| HR-HIVE – 2 doses | 2.4 |  | 28.328 | 236 | dominated |
| HR-HIVE – Extended | 2.4 |  | 28.334 | 238 | 372 |
| HIVE – 1 dose | 2.3 |  | 28.352 | 252 | dominated |
| HIVE – 2 doses | 2.1 |  | 28.384 | 266 | dominated |
| HIVE – Extended | 1.9 |  | 28.419 | 277 | 457^†,‡^ |
| ***18 month early infant diagnosis uptake: 100%*** | | |  |  |  |
| Standard of care | 2.8 |  | 28.281 | 256 | *Reference* |
| HR-HIVE – 1 dose | 2.5 |  | 28.332 | 242 | cost-saving |
| HR-HIVE – 2 doses | 2.4 |  | 28.338 | 245 | dominated |
| HR-HIVE – Extended | 2.4 |  | 28.344 | 246 | 368 |
| HIVE – 1 dose | 2.3 |  | 28.363 | 261 | dominated |
| HIVE – 2 doses | 2.1 |  | 28.393 | 274 | dominated |
| HIVE – Extended | 1.9 |  | 28.424 | 285 | 471^†,‡^ |
| **Zimbabwe [CET: ICER < $293/YLS (20% GDP per capita), ICER < $732 (50% GDP per capita)]** | | | | | |
| ***18 month early infant diagnosis uptake: 0%*** | | |  |  |  |
| Standard of care | 6.3 |  | 27.147 | 402 | *Reference* |
| HR-HIVE – 1 dose | 5.7 |  | 27.249 | 382 | cost-saving^†^ |
| HR-HIVE – 2 doses | 5.6 |  | 27.265 | 387 | 357 |
| HIVE – 1 dose | 5.4 |  | 27.287 | 406 | dominated |
| HR-HIVE – Extended | 5.3 |  | 27.295 | 399 | 405 |
| HIVE – 2 doses | 5.1 |  | 27.344 | 432 | dominated |
| HIVE – Extended | 4.1 |  | 27.477 | 483 | 460^‡^ |
| ***18 month early infant diagnosis uptake: 100%*** | | |  |  |  |
| Standard of care | 6.3 |  | 27.194 | 442 | *Reference* |
| HR-HIVE – 1 dose | 5.7 |  | 27.295 | 420 | cost-saving^†^ |
| HR-HIVE – 2 doses | 5.6 |  | 27.309 | 425 | dominated |
| HIVE – 1 dose | 5.4 |  | 27.332 | 444 | dominated |
| HR-HIVE – Extended | 5.3 |  | 27.336 | 435 | 358 |
| HIVE – 2 doses | 5.1 |  | 27.383 | 468 | dominated |
| HIVE – Extended | 4.1 |  | 27.506 | 508 | 432^‡^ |

The most cost-effective bNAb implementation strategy was the strategy that offered the greatest increase in overall population life expectancy while still having an ICER less than the cost-effectiveness threshold when compared to the next best performing, non-dominated strategy.

yr, year; ICER, incremental cost-effectiveness ratio; YLS, year of life saved; CET, cost-effectiveness threshold; HR-HIVE, high-risk HIV-exposed infants; HIVE, all HIV-exposed infants.

^†^ Indicates the preferred strategy at a cost-effectiveness threshold of 20% GDP per capita.

^‡^ Indicates the preferred strategy at a cost-effectiveness threshold of 50% GDP per capita.

# **Table A32. One-way sensitivity analysis: overall early infant diagnosis uptake**

| **Country/strategy** | **Clinical outcomes** |  | **Lifetime efficacy and costs** | | |
| --- | --- | --- | --- | --- | --- |
|  | **Total cumulative HIV incidence (%)** |  | **Discounted life expectancy (yrs)** | **Discounted**  **costs ($)** | **ICER ($/YLS)** |
| **Côte d’Ivoire [CET: ICER < $455/YLS (20% GDP per capita), ICER < $1,138 (50% GDP per capita)]** | | | | | |
| ***Overall early infant diagnosis uptake: low uptake at all time points*** | | | |  |  |
| Standard of care | 8.4 |  | 25.059 | 508 | *Reference* |
| HR-HIVE – 1 dose | 7.6 |  | 25.191 | 486 | cost-saving |
| HR-HIVE – 2 doses | 7.4 |  | 25.210 | 493 | dominated |
| HIVE – 1 dose | 7.4 |  | 25.212 | 501 | dominated |
| HR-HIVE – Extended | 7.1 |  | 25.245 | 508 | 408^†^ |
| HIVE – 2 doses | 7.1 |  | 25.253 | 521 | dominated |
| HIVE – Extended | 6.3 |  | 25.337 | 554 | 498^‡^ |
| ***Overall early infant diagnosis uptake: high uptake at all time points*** | | | |  |  |
| Standard of care | 8.4 |  | 25.175 | 616 | *Reference* |
| HR-HIVE – 1 dose | 7.6 |  | 25.298 | 589 | cost-saving |
| HR-HIVE – 2 doses | 7.4 |  | 25.317 | 595 | 314 |
| HIVE – 1 dose | 7.4 |  | 25.319 | 604 | dominated |
| HR-HIVE – Extended | 7.1 |  | 25.345 | 605 | 351^†^ |
| HIVE – 2 doses | 7.1 |  | 25.360 | 621 | dominated |
| HIVE – Extended | 6.3 |  | 25.425 | 643 | 473^‡^ |
| **South Africa [CET: ICER < $1,200/YLS (20% GDP per capita), ICER < $3,001 (50% GDP per capita)]** | | | | | |
| ***Overall early infant diagnosis uptake: low uptake at all time points*** | | |  |  |  |
| Standard of care | 2.8 |  | 28.246 | 211 | *Reference* |
| HR-HIVE – 1 dose | 2.5 |  | 28.301 | 199 | cost-saving |
| HR-HIVE – 2 doses | 2.4 |  | 28.306 | 201 | dominated |
| HR-HIVE – Extended | 2.4 |  | 28.313 | 203 | 359 |
| HIVE – 1 dose | 2.3 |  | 28.334 | 218 | dominated |
| HIVE – 2 doses | 2.1 |  | 28.365 | 231 | dominated |
| HIVE – Extended | 1.9 |  | 28.401 | 243 | 451^†,‡^ |
| ***Overall early infant diagnosis uptake: high uptake at all time points*** | | |  |  |  |
| Standard of care | 2.8 |  | 28.287 | 273 | *Reference* |
| HR-HIVE – 1 dose | 2.5 |  | 28.337 | 258 | cost-saving |
| HR-HIVE – 2 doses | 2.4 |  | 28.342 | 261 | dominated |
| HR-HIVE – Extended | 2.4 |  | 28.348 | 263 | 371 |
| HIVE – 1 dose | 2.3 |  | 28.369 | 277 | dominated |
| HIVE – 2 doses | 2.1 |  | 28.397 | 289 | dominated |
| HIVE – Extended | 1.9 |  | 28.429 | 300 | 462^†,‡^ |
| **Zimbabwe [CET: ICER < $293/YLS (20% GDP per capita), ICER < $732 (50% GDP per capita)]** | | | | | |
| ***Overall early infant diagnosis uptake: low uptake at all time points*** | | | |  |  |
| Standard of care | 6.3 |  | 27.124 | 373 | *Reference* |
| HR-HIVE – 1 dose | 5.7 |  | 27.229 | 355 | cost-saving^†^ |
| HR-HIVE – 2 doses | 5.6 |  | 27.245 | 361 | 359 |
| HIVE – 1 dose | 5.4 |  | 27.267 | 381 | dominated |
| HR-HIVE – Extended | 5.3 |  | 27.276 | 373 | 407 |
| HIVE – 2 doses | 5.1 |  | 27.326 | 406 | dominated |
| HIVE – Extended | 4.1 |  | 27.462 | 458 | 458^‡^ |
| ***Overall early infant diagnosis uptake: high uptake at all time points*** | | | |  |  |
| Standard of care | 6.3 |  | 27.225 | 487 | *Reference* |
| HR-HIVE – 1 dose | 5.7 |  | 27.320 | 463 | cost-saving^†^ |
| HR-HIVE – 2 doses | 5.6 |  | 27.335 | 467 | 336 |
| HIVE – 1 dose | 5.4 |  | 27.356 | 485 | dominated |
| HR-HIVE – Extended | 5.3 |  | 27.361 | 477 | 350 |
| HIVE – 2 doses | 5.1 |  | 27.408 | 510 | dominated |
| HIVE – Extended | 4.1 |  | 27.528 | 549 | 435^‡^ |

The most cost-effective bNAb implementation strategy was the strategy that offered the greatest increase in overall population life expectancy while still having an ICER less than the cost-effectiveness threshold when compared to the next best performing, non-dominated strategy.

yr, year; ICER, incremental cost-effectiveness ratio; YLS, year of life saved; CET, cost-effectiveness threshold; HR-HIVE, high-risk HIV-exposed infants; HIVE, all HIV-exposed infants.

^†^ Indicates the preferred strategy at a cost-effectiveness threshold of 20% GDP per capita.

^‡^ Indicates the preferred strategy at a cost-effectiveness threshold of 50% GDP per capita.

# **Table A33. One-way sensitivity analysis: early infant diagnosis result return rate, return time, & linkage to care**

| **Country/strategy** | **Clinical outcomes** |  | **Lifetime efficacy and costs** | | |
| --- | --- | --- | --- | --- | --- |
|  | **Total cumulative HIV incidence (%)** |  | **Discounted life expectancy (yrs)** | **Discounted**  **costs ($)** | **ICER ($/YLS)** |
| **Côte d’Ivoire [CET: ICER < $455/YLS (20% GDP per capita), ICER < $1,138 (50% GDP per capita)]** | | | | | |
| ***Worst case scenario*** | | |  |  |  |
| Standard of care | 8.4 |  | 24.995 | 492 | *Reference* |
| HR-HIVE – 1 dose | 7.6 |  | 25.135 | 472 | cost-saving |
| HR-HIVE – 2 doses | 7.4 |  | 25.155 | 480 | 394 |
| HIVE – 1 dose | 7.4 |  | 25.158 | 488 | dominated |
| HR-HIVE – Extended | 7.1 |  | 25.192 | 496 | 419^†^ |
| HIVE – 2 doses | 7.1 |  | 25.202 | 509 | dominated |
| HIVE – Extended | 6.3 |  | 25.290 | 544 | 494^‡^ |
| ***Best case scenario*** | | |  |  |  |
| Standard of care | 8.4 |  | 25.221 | 609 | *Reference* |
| HR-HIVE – 1 dose | 7.6 |  | 25.333 | 575 | cost-saving |
| HR-HIVE – 2 doses | 7.4 |  | 25.349 | 581 | 349 |
| HIVE – 1 dose | 7.4 |  | 25.351 | 589 | dominated |
| HR-HIVE – Extended | 7.1 |  | 25.382 | 593 | 371^†^ |
| HIVE – 2 doses | 7.1 |  | 25.388 | 606 | dominated |
| HIVE – Extended | 6.3 |  | 25.465 | 634 | 491^‡^ |
| **South Africa [CET: ICER < $1,200/YLS (20% GDP per capita), ICER < $3,001 (50% GDP per capita)]** | | | | | |
| ***Worst case scenario*** | | |  |  |  |
| Standard of care | 2.8 |  | 28.261 | 246 | *Reference* |
| HR-HIVE – 1 dose | 2.5 |  | 28.312 | 232 | cost-saving |
| HR-HIVE – 2 doses | 2.4 |  | 28.319 | 235 | dominated |
| HR-HIVE – Extended | 2.4 |  | 28.325 | 237 | 355 |
| HIVE – 1 dose | 2.3 |  | 28.343 | 252 | dominated |
| HIVE – 2 doses | 2.1 |  | 28.375 | 265 | dominated |
| HIVE – Extended | 1.9 |  | 28.413 | 277 | 454^†,‡^ |
| ***Best case scenario*** | | |  |  |  |
| Standard of care | 2.8 |  | 28.240 | 231 | *Reference* |
| HR-HIVE – 1 dose | 2.5 |  | 28.295 | 220 | cost-saving |
| HR-HIVE – 2 doses | 2.4 |  | 28.301 | 223 | dominated |
| HR-HIVE – Extended | 2.4 |  | 28.308 | 225 | 389 |
| HIVE – 1 dose | 2.3 |  | 28.326 | 240 | dominated |
| HIVE – 2 doses | 2.1 |  | 28.359 | 254 | dominated |
| HIVE – Extended | 1.9 |  | 28.396 | 266 | 469^†,‡^ |
| **Zimbabwe [CET: ICER < $293/YLS (20% GDP per capita), ICER < $732 (50% GDP per capita)]** | | | | | |
| ***Worst case scenario*** | | |  |  |  |
| Standard of care | 6.3 |  | 27.104 | 377 | *Reference* |
| HR-HIVE – 1 dose | 5.7 |  | 27.211 | 359 | cost-saving^†^ |
| HR-HIVE – 2 doses | 5.6 |  | 27.227 | 366 | dominated |
| HIVE – 1 dose | 5.4 |  | 27.251 | 384 | dominated |
| HR-HIVE – Extended | 5.3 |  | 27.259 | 378 | 405 |
| HIVE – 2 doses | 5.1 |  | 27.308 | 412 | dominated |
| HIVE – Extended | 4.1 |  | 27.448 | 465 | 459^‡^ |
| ***Best case scenario*** | | |  |  |  |
| Standard of care | 6.3 |  | 27.204 | 438 | *Reference* |
| HR-HIVE – 1 dose | 5.7 |  | 27.299 | 413 | cost-saving^†^ |
| HR-HIVE – 2 doses | 5.6 |  | 27.314 | 419 | 359 |
| HIVE – 1 dose | 5.4 |  | 27.335 | 437 | dominated |
| HR-HIVE – Extended | 5.3 |  | 27.343 | 430 | 386 |
| HIVE – 2 doses | 5.1 |  | 27.389 | 462 | dominated |
| HIVE – Extended | 4.1 |  | 27.517 | 508 | 452^‡^ |

The most cost-effective bNAb implementation strategy was the strategy that offered the greatest increase in overall population life expectancy while still having an ICER less than the cost-effectiveness threshold when compared to the next best performing, non-dominated strategy.

yr, year; ICER, incremental cost-effectiveness ratio; YLS, year of life saved; CET, cost-effectiveness threshold; HR-HIVE, high-risk HIV-exposed infants; HIVE, all HIV-exposed infants.

^†^ Indicates the preferred strategy at a cost-effectiveness threshold of 20% GDP per capita.

^‡^ Indicates the preferred strategy at a cost-effectiveness threshold of 50% GDP per capita.

# **Table A34. One-way sensitivity analysis: bNAb reduction in nucleic acid amplification test sensitivity**

| **Country/strategy** | **Clinical outcomes** |  | **Lifetime efficacy and costs** | | |
| --- | --- | --- | --- | --- | --- |
|  | **Total cumulative HIV incidence (%)** |  | **Discounted life expectancy (yrs)** | **Discounted**  **costs ($)** | **ICER ($/YLS)** |
| **Côte d’Ivoire [CET: ICER < $455/YLS (20% GDP per capita), ICER < $1,138 (50% GDP per capita)]** | | | | | |
| ***bNAb reduction in sensitivity: 25%*** | | |  |  |  |
| Standard of care | 8.4 |  | 25.106 | 550 | *Reference* |
| HR-HIVE – 1 dose | 7.6 |  | 25.225 | 520 | cost-saving |
| HR-HIVE – 2 doses | 7.4 |  | 25.236 | 523 | 284 |
| HIVE – 1 dose | 7.4 |  | 25.245 | 535 | dominated |
| HR-HIVE – Extended | 7.1 |  | 25.266 | 534 | 372^†^ |
| HIVE – 2 doses | 7.1 |  | 25.277 | 549 | dominated |
| HIVE – Extended | 6.3 |  | 25.350 | 576 | 490^‡^ |
| ***bNAb reduction in sensitivity: 50%*** | | |  |  |  |
| Standard of care | 8.4 |  | 25.106 | 550 | *Reference* |
| HR-HIVE – 1 dose | 7.6 |  | 25.216 | 515 | cost-saving |
| HR-HIVE – 2 doses | 7.4 |  | 25.222 | 516 | 82 |
| HIVE – 1 dose | 7.4 |  | 25.235 | 529 | dominated |
| HR-HIVE – Extended | 7.1 |  | 25.246 | 524 | 337^†^ |
| HIVE – 2 doses | 7.1 |  | 25.261 | 541 | dominated |
| HIVE – Extended | 6.3 |  | 25.328 | 564 | 490^‡^ |
| ***bNAb reduction in sensitivity: 75%*** | | |  |  |  |
| Standard of care | 8.4 |  | 25.106 | 550 | *Reference* |
| HR-HIVE – 1 dose | 7.6 |  | 25.207 | 511 | dominated |
| HR-HIVE – 2 doses | 7.4 |  | 25.210 | 509 | cost-saving |
| HIVE – 1 dose | 7.4 |  | 25.226 | 525 | dominated |
| HR-HIVE – Extended | 7.1 |  | 25.229 | 515 | 299^†^ |
| HIVE – 2 doses | 7.1 |  | 25.249 | 534 | dominated |
| HIVE – Extended | 6.3 |  | 25.307 | 553 | 487^‡^ |
| ***bNAb reduction in sensitivity: 100%*** | | |  |  |  |
| Standard of care | 8.4 |  | 25.106 | 550 | *Reference* |
| HR-HIVE – 1 dose | 7.6 |  | 25.199 | 506 | dominated |
| HR-HIVE – 2 doses | 7.4 |  | 25.202 | 505 | cost-saving |
| HR-HIVE – Extended | 7.1 |  | 25.214 | 508 | 179^†^ |
| HIVE – 1 dose | 7.4 |  | 25.217 | 520 | dominated |
| HIVE – 2 doses | 7.1 |  | 25.240 | 529 | dominated |
| HIVE – Extended | 6.3 |  | 25.289 | 544 | 484^‡^ |
| **South Africa [CET: ICER < $1,200/YLS (20% GDP per capita), ICER < $3,001 (50% GDP per capita)]** | | | | | |
| ***bNAb reduction in sensitivity: 25%*** | | |  |  |  |
| Standard of care | 2.8 |  | 28.274 | 249 | *Reference* |
| HR-HIVE – 1 dose | 2.5 |  | 28.321 | 233 | cost-saving |
| HR-HIVE – 2 doses | 2.4 |  | 28.326 | 235 | dominated |
| HR-HIVE – Extended | 2.4 |  | 28.331 | 237 | 379 |
| HIVE – 1 dose | 2.3 |  | 28.349 | 251 | dominated |
| HIVE – 2 doses | 2.1 |  | 28.378 | 263 | dominated |
| HIVE – Extended | 1.9 |  | 28.412 | 275 | 468^†,‡^ |
| ***bNAb reduction in sensitivity: 50%*** | | |  |  |  |
| Standard of care | 2.8 |  | 28.274 | 249 | *Reference* |
| HR-HIVE – 1 dose | 2.5 |  | 28.316 | 231 | cost-saving |
| HR-HIVE – 2 doses | 2.4 |  | 28.320 | 233 | dominated |
| HR-HIVE – Extended | 2.4 |  | 28.325 | 234 | 360 |
| HIVE – 1 dose | 2.3 |  | 28.342 | 248 | dominated |
| HIVE – 2 doses | 2.1 |  | 28.370 | 260 | dominated |
| HIVE – Extended | 1.9 |  | 28.403 | 271 | 466^†,‡^ |
| ***bNAb reduction in sensitivity: 75%*** | | |  |  |  |
| Standard of care | 2.8 |  | 28.274 | 249 | *Reference* |
| HR-HIVE – 1 dose | 2.5 |  | 28.311 | 229 | cost-saving |
| HR-HIVE – 2 doses | 2.4 |  | 28.315 | 230 | dominated |
| HR-HIVE – Extended | 2.4 |  | 28.319 | 232 | 350 |
| HIVE – 1 dose | 2.3 |  | 28.336 | 245 | dominated |
| HIVE – 2 doses | 2.1 |  | 28.363 | 257 | dominated |
| HIVE – Extended | 1.9 |  | 28.395 | 267 | 471^†,‡^ |
| ***bNAb reduction in sensitivity: 100%*** | | |  |  |  |
| Standard of care | 2.8 |  | 28.274 | 249 | *Reference* |
| HR-HIVE – 1 dose | 2.5 |  | 28.308 | 227 | cost-saving |
| HR-HIVE – 2 doses | 2.4 |  | 28.312 | 229 | dominated |
| HR-HIVE – Extended | 2.4 |  | 28.315 | 230 | 377 |
| HIVE – 1 dose | 2.3 |  | 28.331 | 243 | dominated |
| HIVE – 2 doses | 2.1 |  | 28.359 | 255 | dominated |
| HIVE – Extended | 1.9 |  | 28.389 | 265 | 472^†,‡^ |
| **Zimbabwe [CET: ICER < $293/YLS (20% GDP per capita), ICER < $732 (50% GDP per capita)]** | | | | | |
| ***bNAb reduction in sensitivity: 25%*** | | |  |  |  |
| Standard of care | 6.3 |  | 27.158 | 411 | *Reference* |
| HR-HIVE – 1 dose | 5.7 |  | 27.254 | 386 | cost-saving |
| HR-HIVE – 2 doses | 5.6 |  | 27.265 | 389 | 267^†^ |
| HIVE – 1 dose | 5.4 |  | 27.291 | 410 | dominated |
| HR-HIVE – Extended | 5.3 |  | 27.291 | 399 | 368 |
| HIVE – 2 doses | 5.1 |  | 27.341 | 432 | dominated |
| HIVE – Extended | 4.1 |  | 27.466 | 478 | 450^‡^ |
| ***bNAb reduction in sensitivity: 50%*** | | |  |  |  |
| Standard of care | 6.3 |  | 27.158 | 411 | *Reference* |
| HR-HIVE – 1 dose | 5.7 |  | 27.248 | 383 | cost-saving |
| HR-HIVE – 2 doses | 5.6 |  | 27.256 | 384 | 154^†^ |
| HR-HIVE – Extended | 5.3 |  | 27.279 | 391 | 325 |
| HIVE – 1 dose | 5.4 |  | 27.284 | 405 | dominated |
| HIVE – 2 doses | 5.1 |  | 27.330 | 426 | dominated |
| HIVE – Extended | 4.1 |  | 27.449 | 467 | 444^‡^ |
| ***bNAb reduction in sensitivity: 75%*** | | |  |  |  |
| Standard of care | 6.3 |  | 27.158 | 411 | *Reference* |
| HR-HIVE – 1 dose | 5.7 |  | 27.243 | 379 | cost-saving |
| HR-HIVE – 2 doses | 5.6 |  | 27.249 | 380 | 71 |
| HR-HIVE – Extended | 5.3 |  | 27.268 | 384 | 257^†^ |
| HIVE – 1 dose | 5.4 |  | 27.278 | 401 | dominated |
| HIVE – 2 doses | 5.1 |  | 27.322 | 421 | dominated |
| HIVE – Extended | 4.1 |  | 27.433 | 457 | 438^‡^ |
| ***bNAb reduction in sensitivity: 100%*** | | |  |  |  |
| Standard of care | 6.3 |  | 27.158 | 411 | *Reference* |
| HR-HIVE – 1 dose | 5.7 |  | 27.238 | 376 | cost-saving |
| HR-HIVE – 2 doses | 5.6 |  | 27.245 | 377 | 126 |
| HR-HIVE – Extended | 5.3 |  | 27.258 | 379 | 131^†^ |
| HIVE – 1 dose | 5.4 |  | 27.272 | 397 | dominated |
| HIVE – 2 doses | 5.1 |  | 27.317 | 418 | dominated |
| HIVE – Extended | 4.1 |  | 27.420 | 449 | 433^‡^ |

The most cost-effective bNAb implementation strategy was the strategy that offered the greatest increase in overall population life expectancy while still having an ICER less than the cost-effectiveness threshold when compared to the next best performing, non-dominated strategy.

yr, year; ICER, incremental cost-effectiveness ratio; YLS, year of life saved; CET, cost-effectiveness threshold; HR-HIVE, high-risk HIV-exposed infants; HIVE, all HIV-exposed infants.

^†^ Indicates the preferred strategy at a cost-effectiveness threshold of 20% GDP per capita.

^‡^ Indicates the preferred strategy at a cost-effectiveness threshold of 50% GDP per capita.

# **Table A35. One-way sensitivity analysis: bNAb reduction in antibody test specificity (stop bNAb if positive)**

| **Country/strategy** | **Clinical outcomes** |  | **Lifetime efficacy and costs** | | |
| --- | --- | --- | --- | --- | --- |
|  | **Total cumulative HIV incidence (%)** |  | **Discounted life expectancy (yrs)** | **Discounted**  **costs ($)** | **ICER ($/YLS)** |
| **Côte d’Ivoire [CET: ICER < $455/YLS (20% GDP per capita), ICER < $1,138 (50% GDP per capita)]** | | | | | |
| ***bNAb reduction in specificity: 25%*** | | |  |  |  |
| Standard of care | 8.4 |  | 25.106 | 550 | *Reference* |
| HR-HIVE – 1 dose | 7.6 |  | 25.234 | 525 | cost-saving |
| HR-HIVE – 2 doses | 7.4 |  | 25.252 | 532 | 368 |
| HIVE – 1 dose | 7.4 |  | 25.255 | 540 | dominated |
| HR-HIVE – Extended | 7.1 |  | 25.286 | 545 | 388^†^ |
| HIVE – 2 doses | 7.1 |  | 25.295 | 559 | dominated |
| HIVE – Extended | 6.3 |  | 25.374 | 589 | 497^‡^ |
| ***bNAb reduction in specificity: 50%*** | | |  |  |  |
| Standard of care | 8.4 |  | 25.106 | 550 | *Reference* |
| HR-HIVE – 1 dose | 7.6 |  | 25.234 | 525 | cost-saving |
| HR-HIVE – 2 doses | 7.4 |  | 25.252 | 532 | 368 |
| HIVE – 1 dose | 7.4 |  | 25.255 | 540 | dominated |
| HR-HIVE – Extended | 7.1 |  | 25.286 | 545 | 389^†^ |
| HIVE – 2 doses | 7.1 |  | 25.295 | 559 | dominated |
| HIVE – Extended | 6.3 |  | 25.374 | 589 | 496^‡^ |
| ***bNAb reduction in specificity: 75%*** | | |  |  |  |
| Standard of care | 8.4 |  | 25.106 | 550 | *Reference* |
| HR-HIVE – 1 dose | 7.6 |  | 25.234 | 525 | cost-saving |
| HR-HIVE – 2 doses | 7.4 |  | 25.252 | 532 | 368 |
| HIVE – 1 dose | 7.4 |  | 25.255 | 540 | dominated |
| HR-HIVE – Extended | 7.1 |  | 25.286 | 545 | 390^†^ |
| HIVE – 2 doses | 7.1 |  | 25.295 | 559 | dominated |
| HIVE – Extended | 6.3 |  | 25.374 | 589 | 497^‡^ |
| ***bNAb reduction in specificity: 100%*** | | |  |  |  |
| Standard of care | 8.4 |  | 25.106 | 550 | *Reference* |
| HR-HIVE – 1 dose | 7.6 |  | 25.234 | 525 | cost-saving |
| HR-HIVE – 2 doses | 7.4 |  | 25.252 | 532 | 368 |
| HIVE – 1 dose | 7.4 |  | 25.255 | 540 | dominated |
| HR-HIVE – Extended | 7.1 |  | 25.286 | 545 | 390^†^ |
| HIVE – 2 doses | 7.1 |  | 25.295 | 559 | dominated |
| HIVE – Extended | 6.3 |  | 25.374 | 589 | 497^‡^ |
| **South Africa [CET: ICER < $1,200/YLS (20% GDP per capita), ICER < $3,001 (50% GDP per capita)]** | | | | | |
| ***bNAb reduction in specificity: 25%*** | | |  |  |  |
| Standard of care | 2.8 |  | 28.274 | 249 | *Reference* |
| HR-HIVE – 1 dose | 2.5 |  | 28.325 | 235 | cost-saving |
| HR-HIVE – 2 doses | 2.4 |  | 28.331 | 238 | dominated |
| HR-HIVE – Extended | 2.4 |  | 28.337 | 240 | 377 |
| HIVE – 1 dose | 2.3 |  | 28.355 | 254 | dominated |
| HIVE – 2 doses | 2.1 |  | 28.386 | 267 | dominated |
| HIVE – Extended | 1.9 |  | 28.421 | 279 | 464^†,‡^ |
| ***bNAb reduction in specificity: 50%*** | | |  |  |  |
| Standard of care | 2.8 |  | 28.274 | 249 | *Reference* |
| HR-HIVE – 1 dose | 2.5 |  | 28.325 | 235 | cost-saving |
| HR-HIVE – 2 doses | 2.4 |  | 28.331 | 238 | dominated |
| HR-HIVE – Extended | 2.4 |  | 28.337 | 240 | 377 |
| HIVE – 1 dose | 2.3 |  | 28.355 | 254 | dominated |
| HIVE – 2 doses | 2.1 |  | 28.386 | 267 | dominated |
| HIVE – Extended | 1.9 |  | 28.420 | 279 | 465^†,‡^ |
| ***bNAb reduction in specificity: 75%*** | | |  |  |  |
| Standard of care | 2.8 |  | 28.274 | 249 | *Reference* |
| HR-HIVE – 1 dose | 2.5 |  | 28.325 | 235 | cost-saving |
| HR-HIVE – 2 doses | 2.4 |  | 28.331 | 238 | dominated |
| HR-HIVE – Extended | 2.4 |  | 28.337 | 240 | 377 |
| HIVE – 1 dose | 2.3 |  | 28.355 | 254 | dominated |
| HIVE – 2 doses | 2.1 |  | 28.386 | 267 | dominated |
| HIVE – Extended | 1.9 |  | 28.420 | 279 | 464^†,‡^ |
| ***bNAb reduction in specificity: 100%*** | | |  |  |  |
| Standard of care | 2.8 |  | 28.274 | 249 | *Reference* |
| HR-HIVE – 1 dose | 2.5 |  | 28.325 | 235 | cost-saving |
| HR-HIVE – 2 doses | 2.4 |  | 28.331 | 238 | dominated |
| HR-HIVE – Extended | 2.4 |  | 28.337 | 240 | 377 |
| HIVE – 1 dose | 2.3 |  | 28.355 | 254 | dominated |
| HIVE – 2 doses | 2.1 |  | 28.386 | 267 | dominated |
| HIVE – Extended | 1.9 |  | 28.420 | 279 | 465^†,‡^ |
| **Zimbabwe [CET: ICER < $293/YLS (20% GDP per capita), ICER < $732 (50% GDP per capita)]** | | | | | |
| ***bNAb reduction in specificity: 25%*** | | |  |  |  |
| Standard of care | 6.3 |  | 27.158 | 411 | *Reference* |
| HR-HIVE – 1 dose | 5.7 |  | 27.260 | 390 | cost-saving^†^ |
| HR-HIVE – 2 doses | 5.6 |  | 27.275 | 396 | 367 |
| HIVE – 1 dose | 5.4 |  | 27.298 | 414 | dominated |
| HR-HIVE – Extended | 5.3 |  | 27.304 | 407 | 390 |
| HIVE – 2 doses | 5.1 |  | 27.353 | 440 | dominated |
| HIVE – Extended | 4.1 |  | 27.483 | 489 | 455^‡^ |
| ***bNAb reduction in specificity: 50%*** | | |  |  |  |
| Standard of care | 6.3 |  | 27.158 | 411 | *Reference* |
| HR-HIVE – 1 dose | 5.7 |  | 27.260 | 390 | cost-saving^†^ |
| HR-HIVE – 2 doses | 5.6 |  | 27.275 | 396 | 367 |
| HIVE – 1 dose | 5.4 |  | 27.298 | 414 | dominated |
| HR-HIVE – Extended | 5.3 |  | 27.304 | 407 | 392 |
| HIVE – 2 doses | 5.1 |  | 27.353 | 440 | dominated |
| HIVE – Extended | 4.1 |  | 27.483 | 489 | 456^‡^ |
| ***bNAb reduction in specificity: 75%*** | | |  |  |  |
| Standard of care | 6.3 |  | 27.158 | 411 | *Reference* |
| HR-HIVE – 1 dose | 5.7 |  | 27.260 | 390 | cost-saving^†^ |
| HR-HIVE – 2 doses | 5.6 |  | 27.275 | 396 | 367 |
| HIVE – 1 dose | 5.4 |  | 27.298 | 414 | dominated |
| HR-HIVE – Extended | 5.3 |  | 27.304 | 407 | 393 |
| HIVE – 2 doses | 5.1 |  | 27.353 | 440 | dominated |
| HIVE – Extended | 4.1 |  | 27.483 | 489 | 457^‡^ |
| ***bNAb reduction in specificity: 100%*** | | |  |  |  |
| Standard of care | 6.3 |  | 27.158 | 411 | *Reference* |
| HR-HIVE – 1 dose | 5.7 |  | 27.260 | 390 | cost-saving^†^ |
| HR-HIVE – 2 doses | 5.6 |  | 27.275 | 396 | 367 |
| HIVE – 1 dose | 5.4 |  | 27.298 | 414 | dominated |
| HR-HIVE – Extended | 5.3 |  | 27.304 | 407 | 393 |
| HIVE – 2 doses | 5.1 |  | 27.353 | 440 | dominated |
| HIVE – Extended | 4.1 |  | 27.483 | 489 | 458^‡^ |

The most cost-effective bNAb implementation strategy was the strategy that offered the greatest increase in overall population life expectancy while still having an ICER less than the cost-effectiveness threshold when compared to the next best performing, non-dominated strategy.

yr, year; ICER, incremental cost-effectiveness ratio; YLS, year of life saved; CET, cost-effectiveness threshold; HR-HIVE, high-risk HIV-exposed infants; HIVE, all HIV-exposed infants.

^†^ Indicates the preferred strategy at a cost-effectiveness threshold of 20% GDP per capita.

^‡^ Indicates the preferred strategy at a cost-effectiveness threshold of 50% GDP per capita.

# **Table A36. One-way sensitivity analysis: bNAb reduction in antibody test specificity (continue bNAb if positive)**

| **Country/strategy** | **Clinical outcomes** |  | **Lifetime efficacy and costs** | | |
| --- | --- | --- | --- | --- | --- |
|  | **Total cumulative HIV incidence (%)** |  | **Discounted life expectancy (yrs)** | **Discounted**  **costs ($)** | **ICER ($/YLS)** |
| **Côte d’Ivoire [CET: ICER < $455/YLS (20% GDP per capita), ICER < $1,138 (50% GDP per capita)]** | | | | | |
| ***bNAb reduction in specificity: 25%*** | | |  |  |  |
| Standard of care | 8.4 |  | 25.106 | 550 | *Reference* |
| HR-HIVE – 1 dose | 7.6 |  | 25.234 | 525 | cost-saving |
| HR-HIVE – 2 doses | 7.4 |  | 25.252 | 532 | 391 |
| HIVE – 1 dose | 7.4 |  | 25.255 | 540 | dominated |
| HR-HIVE – Extended | 7.1 |  | 25.286 | 547 | 428^†^ |
| HIVE – 2 doses | 7.1 |  | 25.295 | 559 | dominated |
| HIVE – Extended | 6.3 |  | 25.375 | 591 | 501^‡^ |
| ***bNAb reduction in specificity: 50%*** | | |  |  |  |
| Standard of care | 8.4 |  | 25.106 | 550 | *Reference* |
| HR-HIVE – 1 dose | 7.6 |  | 25.234 | 525 | cost-saving |
| HR-HIVE – 2 doses | 7.4 |  | 25.252 | 532 | 391 |
| HIVE – 1 dose | 7.4 |  | 25.255 | 540 | dominated |
| HR-HIVE – Extended | 7.1 |  | 25.286 | 547 | 428^†^ |
| HIVE – 2 doses | 7.1 |  | 25.295 | 559 | dominated |
| HIVE – Extended | 6.3 |  | 25.375 | 591 | 501^‡^ |
| ***bNAb reduction in specificity: 75%*** | | |  |  |  |
| Standard of care | 8.4 |  | 25.106 | 550 | *Reference* |
| HR-HIVE – 1 dose | 7.6 |  | 25.234 | 525 | cost-saving |
| HR-HIVE – 2 doses | 7.4 |  | 25.252 | 532 | 391 |
| HIVE – 1 dose | 7.4 |  | 25.255 | 540 | dominated |
| HR-HIVE – Extended | 7.1 |  | 25.286 | 547 | 441^†^ |
| HIVE – 2 doses | 7.1 |  | 25.295 | 559 | dominated |
| HIVE – Extended | 6.3 |  | 25.375 | 591 | 502^‡^ |
| ***bNAb reduction in specificity: 100%*** | | |  |  |  |
| Standard of care | 8.4 |  | 25.106 | 550 | *Reference* |
| HR-HIVE – 1 dose | 7.6 |  | 25.234 | 525 | cost-saving |
| HR-HIVE – 2 doses | 7.4 |  | 25.252 | 532 | 391 |
| HIVE – 1 dose | 7.4 |  | 25.255 | 540 | dominated |
| HR-HIVE – Extended | 7.1 |  | 25.286 | 547 | 441^†^ |
| HIVE – 2 doses | 7.1 |  | 25.295 | 559 | dominated |
| HIVE – Extended | 6.3 |  | 25.375 | 591 | 502^‡^ |
| **South Africa [CET: ICER < $1,200/YLS (20% GDP per capita), ICER < $3,001 (50% GDP per capita)]** | | | | | |
| ***bNAb reduction in specificity: 25%*** | | |  |  |  |
| Standard of care | 2.8 |  | 28.274 | 249 | *Reference* |
| HR-HIVE – 1 dose | 2.5 |  | 28.325 | 235 | cost-saving |
| HR-HIVE – 2 doses | 2.4 |  | 28.331 | 238 | dominated |
| HR-HIVE – Extended | 2.4 |  | 28.337 | 240 | 391 |
| HIVE – 1 dose | 2.3 |  | 28.355 | 254 | dominated |
| HIVE – 2 doses | 2.1 |  | 28.386 | 268 | dominated |
| HIVE – Extended | 1.9 |  | 28.421 | 279 | 464^†,‡^ |
| ***bNAb reduction in specificity: 50%*** | | |  |  |  |
| Standard of care | 2.8 |  | 28.274 | 249 | *Reference* |
| HR-HIVE – 1 dose | 2.5 |  | 28.325 | 235 | cost-saving |
| HR-HIVE – 2 doses | 2.4 |  | 28.331 | 238 | dominated |
| HR-HIVE – Extended | 2.4 |  | 28.337 | 240 | 391 |
| HIVE – 1 dose | 2.3 |  | 28.355 | 254 | dominated |
| HIVE – 2 doses | 2.1 |  | 28.386 | 268 | dominated |
| HIVE – Extended | 1.9 |  | 28.421 | 279 | 464^†,‡^ |
| ***bNAb reduction in specificity: 75%*** | | |  |  |  |
| Standard of care | 2.8 |  | 28.274 | 249 | *Reference* |
| HR-HIVE – 1 dose | 2.5 |  | 28.325 | 235 | cost-saving |
| HR-HIVE – 2 doses | 2.4 |  | 28.331 | 238 | dominated |
| HR-HIVE – Extended | 2.4 |  | 28.337 | 240 | 391 |
| HIVE – 1 dose | 2.3 |  | 28.355 | 254 | dominated |
| HIVE – 2 doses | 2.1 |  | 28.386 | 268 | dominated |
| HIVE – Extended | 1.9 |  | 28.421 | 279 | 464^†,‡^ |
| ***bNAb reduction in specificity: 100%*** | | |  |  |  |
| Standard of care | 2.8 |  | 28.274 | 249 | *Reference* |
| HR-HIVE – 1 dose | 2.5 |  | 28.325 | 235 | cost-saving |
| HR-HIVE – 2 doses | 2.4 |  | 28.331 | 238 | dominated |
| HR-HIVE – Extended | 2.4 |  | 28.337 | 240 | 391 |
| HIVE – 1 dose | 2.3 |  | 28.355 | 254 | dominated |
| HIVE – 2 doses | 2.1 |  | 28.386 | 268 | dominated |
| HIVE – Extended | 1.9 |  | 28.421 | 279 | 464^†,‡^ |
| **Zimbabwe [CET: ICER < $293/YLS (20% GDP per capita), ICER < $732 (50% GDP per capita)]** | | | | | |
| ***bNAb reduction in specificity: 25%*** | | |  |  |  |
| Standard of care | 6.3 |  | 27.158 | 411 | *Reference* |
| HR-HIVE – 1 dose | 5.7 |  | 27.260 | 390 | cost-saving^†^ |
| HR-HIVE – 2 doses | 5.6 |  | 27.275 | 396 | 368 |
| HIVE – 1 dose | 5.4 |  | 27.298 | 414 | dominated |
| HR-HIVE – Extended | 5.3 |  | 27.304 | 408 | 434 |
| HIVE – 2 doses | 5.1 |  | 27.353 | 440 | dominated |
| HIVE – Extended | 4.1 |  | 27.484 | 491 | 459^‡^ |
| ***bNAb reduction in specificity: 50%*** | | |  |  |  |
| Standard of care | 6.3 |  | 27.158 | 411 | *Reference* |
| HR-HIVE – 1 dose | 5.7 |  | 27.260 | 390 | cost-saving^†^ |
| HR-HIVE – 2 doses | 5.6 |  | 27.275 | 396 | 368 |
| HIVE – 1 dose | 5.4 |  | 27.298 | 414 | dominated |
| HR-HIVE – Extended | 5.3 |  | 27.304 | 408 | 435 |
| HIVE – 2 doses | 5.1 |  | 27.353 | 440 | dominated |
| HIVE – Extended | 4.1 |  | 27.484 | 491 | 459^‡^ |
| ***bNAb reduction in specificity: 75%*** | | |  |  |  |
| Standard of care | 6.3 |  | 27.158 | 411 | *Reference* |
| HR-HIVE – 1 dose | 5.7 |  | 27.260 | 390 | cost-saving^†^ |
| HR-HIVE – 2 doses | 5.6 |  | 27.275 | 396 | 368 |
| HIVE – 1 dose | 5.4 |  | 27.298 | 414 | dominated |
| HR-HIVE – Extended | 5.3 |  | 27.304 | 408 | 435 |
| HIVE – 2 doses | 5.1 |  | 27.353 | 440 | dominated |
| HIVE – Extended | 4.1 |  | 27.484 | 491 | 459^‡^ |
| ***bNAb reduction in specificity: 100%*** | | |  |  |  |
| Standard of care | 6.3 |  | 27.158 | 411 | *Reference* |
| HR-HIVE – 1 dose | 5.7 |  | 27.260 | 390 | cost-saving^†^ |
| HR-HIVE – 2 doses | 5.6 |  | 27.275 | 396 | 368 |
| HIVE – 1 dose | 5.4 |  | 27.298 | 414 | dominated |
| HR-HIVE – Extended | 5.3 |  | 27.304 | 408 | 435 |
| HIVE – 2 doses | 5.1 |  | 27.353 | 440 | dominated |
| HIVE – Extended | 4.1 |  | 27.484 | 492 | 463^‡^ |

The most cost-effective bNAb implementation strategy was the strategy that offered the greatest increase in overall population life expectancy while still having an ICER less than the cost-effectiveness threshold when compared to the next best performing, non-dominated strategy.

yr, year; ICER, incremental cost-effectiveness ratio; YLS, year of life saved; CET, cost-effectiveness threshold; HR-HIVE, high-risk HIV-exposed infants; HIVE, all HIV-exposed infants.

^†^ Indicates the preferred strategy at a cost-effectiveness threshold of 20% GDP per capita.

^‡^ Indicates the preferred strategy at a cost-effectiveness threshold of 50% GDP per capita.

# **Table A37. One-way sensitivity analysis: 1^st^-line pediatric antiretroviral therapy efficacy**

| **Country/strategy** | **Clinical outcomes** |  | **Lifetime efficacy and costs** | | |
| --- | --- | --- | --- | --- | --- |
|  | **Total cumulative HIV incidence (%)** |  | **Discounted life expectancy (yrs)** | **Discounted**  **costs ($)** | **ICER ($/YLS)** |
| **Côte d’Ivoire [CET: ICER < $455/YLS (20% GDP per capita), ICER < $1,138 (50% GDP per capita)]** | | | | | |
| ***ART efficacy: 95%*** | | |  |  |  |
| Standard of care | 8.4% |  | 25.128 | 488 | *Reference* |
| HR-HIVE – 1 dose | 7.6% |  | 25.252 | 468 | cost-saving |
| HR-HIVE – 2 doses | 7.4% |  | 25.270 | 476 | 454^†^ |
| HIVE – 1 dose | 7.4% |  | 25.272 | 483 | dominated |
| HR-HIVE – Extended | 7.1% |  | 25.303 | 493 | 529 |
| HIVE – 2 doses | 7.1% |  | 25.313 | 505 | dominated |
| HIVE – Extended | 6.3% |  | 25.390 | 544 | 581^‡^ |
| **South Africa [CET: ICER < $1,200/YLS (20% GDP per capita), ICER < $3,001 (50% GDP per capita)]** | | | | | |
| ***ART efficacy: 95%*** | | |  |  |  |
| Standard of care | 2.8% |  | 28.278 | 244 | *Reference* |
| HR-HIVE – 1 dose | 2.5% |  | 28.328 | 231 | cost-saving |
| HR-HIVE – 2 doses | 2.4% |  | 28.334 | 233 | dominated |
| HR-HIVE – Extended | 2.4% |  | 28.340 | 236 | 411 |
| HIVE – 1 dose | 2.3% |  | 28.357 | 250 | dominated |
| HIVE – 2 doses | 2.1% |  | 28.388 | 264 | dominated |
| HIVE – Extended | 1.9% |  | 28.423 | 276 | 486^†,‡^ |
| **Zimbabwe [CET: ICER < $293/YLS (20% GDP per capita), ICER < $732 (50% GDP per capita)]** | | | | | |
| ***ART efficacy: 95%*** | | |  |  |  |
| Standard of care | 6.3% |  | 27.171 | 406 | *Reference* |
| HR-HIVE – 1 dose | 5.7% |  | 27.270 | 385 | cost-saving^†^ |
| HR-HIVE – 2 doses | 5.6% |  | 27.285 | 390 | 387 |
| HIVE – 1 dose | 5.4% |  | 27.308 | 409 | dominated |
| HR-HIVE – Extended | 5.3% |  | 27.314 | 403 | 438 |
| HIVE – 2 doses | 5.1% |  | 27.362 | 435 | dominated |
| HIVE – Extended | 4.1% |  | 27.493 | 488 | 474^‡^ |

The preferred bNAb implementation strategy was the strategy that offered the greatest increase in overall population life expectancy while still having an ICER less than the cost-effectiveness threshold when compared to the next best performing, non-dominated strategy.

yr, year; ICER, incremental cost-effectiveness ratio; YLS, year of life saved; CET, cost-effectiveness threshold; HR-HIVE, high-risk HIV-exposed infants; HIVE, all HIV-exposed infants.

^†^ Indicates the preferred strategy at a cost-effectiveness threshold of 20% GDP per capita.

^‡^ Indicates the preferred strategy at a cost-effectiveness threshold of 50% GDP per capita.

# **Table A38. Minimum bNAb efficacy required for cost-effectiveness (50% GDP per capita) of a *HIVE-Extended* strategy for a variety of product characteristics across all 3 settings.**

| **bNAb cost/dose** | **bNAb effect duration** | |
| --- | --- | --- |
|  | **3 months** | **6 months** |
| **Côte d’Ivoire (CET: ICER < $1,138/YLS)** | | |
| **$20** | 20% | 10% |
| **$60** | 40% | 30% |
| **$120** | 80%** | 60% |
| **$200** | Not cost-effective at any bNAb efficacy | 90%** |
| **South Africa (CET: ICER < $3,001/YLS)** | | |
| **$20** | 10% | 10% |
| **$60** | 20% | 10% |
| **$120** | 30% | 30% |
| **$200** | 50% | 40% |
| **Zimbabwe (CET: ICER < $732/YLS)** | | |
| **$20** | 20% | 20% |
| **$60** | 50% | 40% |
| **$120** | 100%** | 70%** |
| **$200** | Not cost-effective at any bNAb efficacy | Not cost-effective at any bNAb efficacy |

bNAb, broadly neutralizing antibody; HIVE, all HIVE-exposed infants; CET, cost-effectiveness threshold; YLS, year of life saved. **denotes combinations of product characteristics that would require bNAb efficacy to be higher than the 60% assumed in the base case in order for a *HIVE-Extended* strategy to be cost-effective.

# **References**

1. Ciaranello AL, Morris BL, Walensky RP, et al. Validation and calibration of a computer simulation model of pediatric HIV infection. PLoS One **2013**; 8:e83389.

2. Ciaranello AL, Doherty K, Penazzato M, et al. Cost-effectiveness of first-line antiretroviral therapy for HIV-infected African children less than 3 years of age. AIDS **2015**; 29:1247–59.

3. Francke JA, Penazzato M, Hou T, et al. Clinical impact and cost-effectiveness of diagnosing HIV infection during early infancy in South Africa: Test timing and frequency. J Infect Dis **2016**; 214:1319–1328.

4. Frank SC, Cohn J, Dunning L, et al. Clinical effect and cost-effectiveness of incorporation of point-of-care assays into early infant HIV diagnosis programmes in Zimbabwe: a modelling study. Lancet HIV **2019**; 6:e182–e190.

5. Walensky RP, Borre ED, Bekker LG, et al. The anticipated clinical and economic effects of 90-90-90 in South Africa. Ann Intern Med **2016**; 165:325–33.

6. Ciaranello AL, Perez F, Keatinge J, et al. What will it take to eliminate pediatric HIV? Reaching WHO target rates of mother-to-child HIV transmission in Zimbabwe: a model-based analysis. PLoS Med **2012**; 9:e1001156.

7. Goldie SJ, Yazdanpanah Y, Losina E, et al. Cost-effectiveness of HIV treatment in resource-poor settings–the case of Côte d’Ivoire. N Engl J Med **2006**; 355:1141–53.

8. Walensky RP, Ross EL, Kumarasamy N, et al. Cost-effectiveness of HIV treatment as prevention in serodiscordant couples. New Engl J Med **2013**; 369:1715–1725. Available at: https://doi.org/10.1056/NEJMsa1214720. Accessed 5 April 2021.

9. Dugdale CM, Ciaranello AL, Bekker L-G, et al. Risks and benefits of dolutegravir- and efavirenz-based strategies for South African women with HIV of child-bearing potential: a modeling study. Ann Intern Med **2019**; 170:614–625.

10. Dunning L, Francke JA, Mallampati D, et al. The value of confirmatory testing in early infant HIV diagnosis programmes in South Africa: A cost-effectiveness analysis. PLoS Med **2017**; 14:e1002446.

11. World Health Organization. Consolidated guidelines on the use of antiretroviral drugs for the treating and preventing HIV infection: recommendations for a public health approach, 2nd ed. 2016. Available at: https://apps.who.int/iris/handle/10665/208825. Accessed 23 July 2020.

12. South African National Department of Health. National Consolidated Guidelines for the Management of HIV in Adults, Adolescents, Children and Infants and Prevention of Mother-to-Child Transmission, 2020. South African National Department of Health, 2020. Available at: https://www.knowledgehub.org.za/elibrary/national-consolidated-guidelines-management-hiv-adults-adolescents-children-and-infants. Accessed 23 July 2020.

13. National Medicines and Therapeutics Policy Advisory Committee, The AIDS and TB Directorate. Guidelines for Antiretroviral Therapy for the Prevention and Treatment of HIV in Zimbabwe. Ministry of Health and Child Care, 2016. Available at: https://depts.washington.edu/edgh/zw/vl/project-resources/ZIM_ART_Guidelines_2016_-_review_final.pdf. Accessed 28 September 2020.

14. Smith ER, Hudgens M, Sheahan AD, et al. Timing of HIV seroreversion among HIV-exposed, breastfed infants in Malawi: Type of HIV rapid test matters. Matern Child Health J **2017**; 21:248–252.

15. Bianchi F, Cohn J, Sacks E, et al. Evaluation of a routine point-of-care intervention for early infant diagnosis of HIV: an observational study in eight African countries. Lancet HIV **2019**; 6:e373–e381.

16. Bianchi F, Nzima V, Chadambuka A, et al. Comparing conventional to point-of-care (POC) early infant diagnosis (EID): Pre and post intervention data from a multi-country evaluation (Abstract #TUSA1302). 2017; Available at: http://programme.ias2017.org/Programme/Session/98. Accessed 14 October 2020.

17. Desmonde S, Bangali M, Amorissani-Folquet M, et al. Effectiveness of a web-based information system to improve HIV early diagnosis and hepatitis B immunization coverages in Abidjan, Cote d’Ivoire. The DEPISTNEO project. 2019; Available at: https://academicmedicaleducation.com/meeting/international-workshop-hiv-pediatrics-2019/abstract/effectiveness-web-based-information.

18. World Health Organization. Update of recommendations on first- and second-line antiretroviral regimens. 2019. Available at: https://www.who.int/publications/i/item/WHO-CDS-HIV-19.15. Accessed 23 April 2021.

19. World Health Organization. Updated recommendations on HIV prevention, infant diagnosis, antiretroviral initiation and monitoring. 2021. Available at: https://www.who.int/publications/i/item/9789240022232. Accessed 23 April 2021.

20. Losina E, Yazdanpanah Y, Deuffic-Burban S, et al. The independent effect of highly active antiretroviral therapy on severe opportunistic disease incidence and mortality in HIV-infected adults in Côte d’Ivoire. Antiviral Therapy **2007**; 12:543–551.

21. Mellors JW, Munoz A, Giorgi JV, et al. Plasma viral load and CD4+ lymphocytes as prognostic markers of HIV-1 infection. Ann Intern Med **1997**; 126:946–954.

22. Chi BH, Yiannoutsos CT, Westfall AO, et al. Universal definition of loss to follow-up in HIV treatment programs: a statistical analysis of 111 facilities in Africa, Asia, and Latin America. PLoS Med **2011**; 8:e1001111.

23. Stover J, Glaubius R, Mofenson L, et al. Updates to the Spectrum/AIM model for estimating key HIV indicators at national and subnational levels. AIDS **2019**; 33 Suppl 3:S227–S234.

24. UNAIDS Reference Group on Estimates, Modelling, and Projections. Modelling Paediatric HIV and the need for ART. 2020. Available at: http://epidem.org/modelling-paediatric-hiv-and-the-need-for-art-october-2020. Accessed 21 April 2021.

25. Sanders GD, Neumann PJ, Basu A, et al. Recommendations for conduct, methodological practices, and reporting of cost-effectiveness analyses: Second panel on cost-effectiveness in health and medicine. JAMA **2016**; 316:1093–103.

26. Desmonde S, Avit D, Petit J, et al. Costs of care of HIV-infected children initiating lopinavir/ritonavir-based antiretroviral therapy before the age of two in Cote d’Ivoire. PLoS One **2016**; 11:e0166466.

27. Menzies NA, Berruti AA, Berzon R, et al. The cost of providing comprehensive HIV treatment in PEPFAR-supported programs. AIDS **2011**; 25:1753–60.

28. Magure T, Manenji A, Gboun M, et al. Zimbabwe National AIDS Spending Assessment: Consolidated Report 2011 and 2012. National AIDS Council of Zimbabwe and UNAIDS, 2012.

29. Anglaret X, Chene G, Attia A, et al. Early chemoprophylaxis with trimethoprim-sulphamethoxazole for HIV-1-infected adults in Abidjan, Côte d’Ivoire: a randomised trial. Cotrimo-CI Study Group. Lancet **1999**; 353:1463–1468.

30. Thomas LS. Costing of HIV/AIDS services at a tertiary level hospital in Gauteng Province. 2007. Available at: http://hdl.handle.net/10539/2008.

31. Massyn N, Barron P, Day C, Padarath A. District Health Barometer 2018/19. Durban, South Africa: Health Systems Trust, 2020. Available at: https://www.hst.org.za/publications/Pages/DISTRICT-HEALTH-BAROMETER-201819.aspx. Accessed 17 August 2020.

32. Holmes CB, Wood R, Badri M, et al. CD4 decline and incidence of opportunistic infections in Cape Town, South Africa: implications for prophylaxis and treatment. J Acquir Immune Defic Syndr **2006**; 42:464–9.

33. The Global Fund. HIV Viral Load and Early Infant Diagnosis Selection and Procurement Information Tool. The Global Fund, 2017. Available at: https://www.theglobalfund.org/media/5765/psm_viralloadearlyinfantdiagnosis_content_en.pdf?u=637298970370000000. Accessed 5 August 2020.

34. The Global Fund. Pooled Procurement Mechanism Reference Pricing: Advanced HIV disease products. The Global Fund, 2020. Available at: https://www.theglobalfund.org/media/9274/ppm_advancedhivproductsreferencepricing_table_en.pdf?u=637319004622070000. Accessed 20 August 2020.

35. Doherty K, Essajee S, Penazzato M, Holmes C, Resch S, Ciaranello A. Estimating age-based antiretroviral therapy costs for HIV-infected children in resource-limited settings based on World Health Organization weight-based dosing recommendations. BMC Health Serv Res **2014**; 14:201.

36. Beste S, Essajee S, Siberry G, et al. Optimal anti-retroviral prophylaxis in infants at high-risk of acquiring human immunodeficiency virus: a systematic review. Pediatr Infect Dis J **2018**; 37:169–175.

37. Kumwenda NI, Hoover DR, Mofenson LM, et al. Extended antiretroviral prophylaxis to reduce breast-milk HIV-1 transmission. N Engl J Med **2008**; 359:119–29.

38. Hudgens MG, Taha TE, Omer SB, et al. Pooled individual data analysis of 5 randomized trials of infant nevirapine prophylaxis to prevent breast-milk HIV-1 transmission. Clin Infect Dis **2013**; 56:131–9.

39. Corey L, Gilbert PB, Juraska M, et al. Two randomized trials of neutralizing antibodies to prevent HIV-1 acquisition. N Engl J Med **2021**; 384:1003–1014. Available at: https://doi.org/10.1056/NEJMoa2031738. Accessed 2 April 2021.

40. Fouda GG, Mahlokozera T, Salazar-Gonzalez JF, et al. Postnatally-transmitted HIV-1 envelope variants have similar neutralization-sensitivity and function to that of nontransmitted breast milk variants. Retrovirology **2013**; 10:3. Available at: https://doi.org/10.1186/1742-4690-10-3. Accessed 23 September 2020.

41. Nakamura KJ, Cerini C, Sobrera ER, et al. Coverage of primary mother-to-child HIV transmission isolates by second-generation broadly neutralizing antibodies. AIDS **2013**; 27:337–46.

42. Russell ES, Ojeda S, Fouda GG, et al. Short communication: HIV type 1 subtype C variants transmitted through the bottleneck of breastfeeding are sensitive to new generation broadly neutralizing antibodies directed against quaternary and CD4-binding site epitopes. AIDS Res Hum Retroviruses **2013**; 29:511–5.

43. Mabuka J, Goo L, Omenda MM, Nduati R, Overbaugh J. HIV-1 maternal and infant variants show similar sensitivity to broadly neutralizing antibodies, but sensitivity varies by subtype. AIDS **2013**; 27:1535–44.

44. Kumar A, Smith CEP, Giorgi EE, et al. Infant transmitted/founder HIV-1 viruses from peripartum transmission are neutralization resistant to paired maternal plasma. PLOS Pathogens **2018**; 14:e1006944. Available at: https://journals.plos.org/plospathogens/article?id=10.1371/journal.ppat.1006944. Accessed 23 September 2020.

45. Martinez DR, Tu JJ, Kumar A, et al. Maternal broadly neutralizing antibodies can select for neutralization-resistant, infant-transmitted/founder HIV variants. mBio **2020**; 11:e00176-20. Available at: https://mbio.asm.org/content/11/2/e00176-20.

46. Cunningham C. Safety and PK of potent anti-HIV monoclonal Ab VRC07-523LS in HIV-exposed infants (Oral Abstract 03.02). 2021; Available at: https://programme.hivr4p.org/Abstract/Abstract/363.

47. Lorenzi JCC, Mendoza P, Cohen YZ, et al. Neutralizing activity of broadly neutralizing anti-HIV-1 antibodies against primary African isolates. J Virol **2020**; 95:e01909-20.

48. Hessell AJ, Jaworski JP, Epson E, et al. Early short-term treatment with neutralizing human monoclonal antibodies halts SHIV infection in infant macaques. Nat Med **2016**; 22:362–8.

49. Shapiro MB, Cheever T, Malherbe DC, et al. Single-dose bNAb cocktail or abbreviated ART post-exposure regimens achieve tight SHIV control without adaptive immunity. Nat Commun **2020**; 11:70. Available at: https://www.nature.com/articles/s41467-019-13972-y. Accessed 2 April 2021.

50. Anderson DJ, Politch JA, Zeitlin L, et al. Systemic and topical use of monoclonal antibodies to prevent the sexual transmission of HIV. AIDS **2017**; 31:1505–1517.

51. COVAX Working Group on delivery costs. Costs of delivering COVID-19 vaccine in 92 AMC countries. 2021. Available at: https://www.who.int/publications/m/item/costs-of-delivering-covid-19-vaccine-in-92-amc-countries. Accessed 23 April 2021.

52. Mvundura M, Lorenson K, Chweya A, et al. Estimating the costs of the vaccine supply chain and service delivery for selected districts in Kenya and Tanzania. Vaccine **2015**; 33:2697–2703.

53. Cunnama L, Abrams EJ, Myer L, et al. Provider- and patient-level costs associated with providing antiretroviral therapy during the postpartum phase to women living with HIV in South Africa: A cost comparison of three postpartum models of care. Trop Med Int Health **2020**; 25:1553–1567.

54. Woods B, Revill P, Sculpher M, Claxton K. Country-level cost-effectiveness thresholds: Initial estimates and the need for further research. Value Health **2016**; 19:929–935.

55. Robinson LA, Hammitt JK, Chang AY, Resch S. Understanding and improving the one and three times GDP per capita cost-effectiveness thresholds. Health Policy Plan **2017**; 32:141–145.

56. Meyer-Rath G, van Rensburg C, Larson B, Jamieson L, Rosen S. Revealed willingness-to-pay versus standard cost-effectiveness thresholds: evidence from the South African HIV investment case. PLoS One **2017**; 12:e0186496.

57. Marseille E, Larson B, Kazi DS, Kahn JG, Rosen S. Thresholds for the cost-effectiveness of interventions: alternative approaches. Bull World Health Organ **2015**; 93:118–24.

58. Ochalek J, Lomas J, Claxton K. Estimating health opportunity costs in low-income and middle-income countries: a novel approach and evidence from cross-country data. BMJ Global Health **2018**; 3:e000964. Available at: https://gh.bmj.com/content/3/6/e000964. Accessed 18 August 2021.

59. Jit M. Informing global cost-effectiveness thresholds using country investment decisions: human papillomavirus vaccine introductions in 2006-2018. Value Health **2021**; 24:61–66.

60. Edoka IP, Stacey NK. Estimating a cost-effectiveness threshold for health care decision-making in South Africa. Health Policy Plan **2020**; 35:546–555.

61. Egbe TO, Tazinya R-MA, Halle-Ekane GE, Egbe E-N, Achidi EA. Estimating HIV incidence during pregnancy and knowledge of prevention of mother-to-child transmission with an ad hoc analysis of potential cofactors. J Pregnancy **2016**; 2016:7397695.

62. Imade GE, Sagay AS, Musa J, et al. Declining rate of infection with maternal human immunodeficiency virus at delivery units in north-central Nigeria. Afr J Reprod Health **2013**; 17:138–145.

63. Dinh T-H, Delaney KP, Goga A, et al. Impact of maternal HIV seroconversion during pregnancy on early mother to child transmission of HIV (MTCT) measured at 4-8 weeks postpartum in South Africa 2011-2012: A national population-based evaluation. PLoS One **2015**; 10:e0125525.

64. Mbizvo MT, Kasule J, Mahomed K, Nathoo K. HIV-1 seroconversion incidence following pregnancy and delivery among women seronegative at recruitment in Harare, Zimbabwe. Cent Afr J Med **2001**; 47:115–118.

65. Morrison CS, Wang J, Van Der Pol B, Padian N, Salata RA, Richardson BA. Pregnancy and the risk of HIV-1 acquisition among women in Uganda and Zimbabwe. AIDS **2007**; 21:1027–1034.

66. Teasdale CA, Abrams EJ, Chiasson MA, Justman J, Blanchard K, Jones HE. Incidence of sexually transmitted infections during pregnancy. PLoS One **2018**; 13:e0197696.

67. Mandala J, Kasonde P, Badru T, Dirks R, Torpey K. HIV retesting of HIV-negative pregnant women in the context of prevention of mother-to-child transmission of HIV in primary health centers in rural Zambia: What did we learn? J Int Assoc Provid AIDS Care **2019**; 18:2325958218823530.

68. Rogers AJ, Akama E, Weke E, et al. Implementation of repeat HIV testing during pregnancy in southwestern Kenya: progress and missed opportunities. J Int AIDS Soc **2017**; 20:e25036. Available at: https://www.ncbi.nlm.nih.gov/pmc/articles/PMC5810348/. Accessed 24 July 2020.

69. Heemelaar S, Habets N, Makukula Z, Roosmalen J van, Akker T van den. Repeat HIV testing during pregnancy and delivery: missed opportunities in a rural district hospital in Zambia. Trop Med Int Health **2015**; 20:277–283. Available at: https://onlinelibrary.wiley.com/doi/abs/10.1111/tmi.12432. Accessed 24 July 2020.

70. de Beer S, Kalk E, Kroon M, et al. A longitudinal analysis of the completeness of maternal HIV testing, including repeat testing in Cape Town, South Africa. J Int AIDS Soc **2020**; 23:e25441. Available at: https://www.ncbi.nlm.nih.gov/pmc/articles/PMC6989397/. Accessed 29 July 2020.

71. Elizabeth Glaser Pediatric AIDS Foundation. Cote d’Ivoire Annual Report 2018. Elizabeth Glaser Pediatric AIDS Foundation, 2019. Available at: https://www.pedaids.org/resource/cote-divoire-annual-report-2018/. Accessed 4 August 2020.

72. Ministère du Plan et du Développement. La Situation des Femmes et des Enfants en Côte d’Ivoire - Enquête à Indicateurs Multiples 2016 - MICS5. 2016. Available at: https://mics-surveys-prod.s3.amazonaws.com/MICS5/West%20and%20Central%20Africa/C%C3%B4te%20d%27Ivoire/2016/Final/Cote%20d%27Ivoire%202016%20MICS_French.pdf. Accessed 6 October 2022.

73. South Africa National Department of Health. South Africa Demographic and Health Survey 2016. 2019. Available at: https://dhsprogram.com/pubs/pdf/FR337/FR337.pdf. Accessed 6 October 2022.

74. Zimbabwe National Statistics Agency (ZIMSTAT), UNICEF. Zimbabwe Multiple Indicator Cluster Survey 2019, Survey Findings Report. Harare, Zimbabwe: ZIMSTAT and UNICEF, 2019. Available at: https://mics-surveys-prod.s3.amazonaws.com/MICS6/Eastern%20and%20Southern%20Africa/Zimbabwe/2019/Survey%20findings/Zimbabwe%202019%20MICS%20Survey%20Findings%20Report-31012020_English.pdf. Accessed 4 August 2020.

75. ICAP at Columbia University. Cote d’Ivoire Population-Based HIV Impact Assessment (CIPHIA) 2017-2018: Summary Sheet. Population-based HIV Impact Assessment (PHIA) Project, 2018. Available at: https://phia.icap.columbia.edu/wp-content/uploads/2018/08/CIPHIA_Cote-DIvoire-SS_FINAL.pdf.

76. South Africa National Department of Health. National Antenatal Sentinel HIV Survey Key Findings. South Africa: 2017. Available at: https://www.nicd.ac.za/wp-content/uploads/2019/07/Antenatal_survey-report_24July19.pdf. Accessed 6 October 2022.

77. McCoy S, Koyuncu A, Kang-Dufour M, et al. Approaching eMTCT in Zimbabwe: Expansion of PMTCT services and declining MTCT, 2012-2018. In: IAS 2019: Abstract #TUPEC473. Mexico City, MX: International AIDS Society, 2019. Available at: http://programme.ias2019.org/Abstract/Abstract/1921. Accessed 6 October 2022.

78. Moyo F, Mazanderani AH, Murray T, Sherman GG, Kufa T. Achieving maternal viral load suppression for elimination of mother-to-child transmission of HIV in South Africa. AIDS **2021**; 35:307–316.

79. Zimbabwe Ministry of Health and Child Care (MOHCC). Zimbabwe Population-Based HIV Impact Assessment (ZIMPHIA) 2020: Summary Sheet. Harare: 2020. Available at: https://phia.icap.columbia.edu/wp-content/uploads/2020/11/ZIMPHIA-2020-Summary-Sheet_Web.pdf. Accessed 28 December 2020.

80. Zimbabwe Ministry of Health and Child Care (MOHCC). Zimbabwe Population-Based HIV Impact Assessment (ZIMPHIA) 2015-2016: Final Report. Harare: 2019. Available at: https://phia.icap.columbia.edu/wp-content/uploads/2019/08/ZIMPHIA-Final-Report_integrated_Web-1.pdf. Accessed 6 October 2022.

81. Joint United Nations Programme on HIV/AIDS (UNAIDS). UNAIDS Data 2020. UNAIDS, 2020. Available at: https://www.unaids.org/en/resources/documents/2020/unaids-data. Accessed 9 August 2021.

82. Elizabeth Glaser Pediatric AIDS Foundation. The Elizabeth Glaser Pediatric AIDS Foundation - Zimbabwe Annual Report, January - December 2018. Elizabeth Glaser Pediatric AIDS Foundation, 2019. Available at: https://www.pedaids.org/resource/the-elizabeth-glaser-pediatric-aids-foundation-zimbabwe-annual-report/. Accessed 4 August 2020.

83. Zimbabwe National Statistics Agency. Demographic and Health Survey 2015. Harare, Zimbabwe: 2016. Available at: https://dhsprogram.com/pubs/pdf/FR322/FR322.pdf.

84. Dinh T, Mushavi A, Balachandra S, et al. Impact of option B+ and maternal HIV RNA viral load on mother-to-child HIV transmission: Findings from an 18-month prospective cohort study of a nationally representative sample of mother-infant pairs, Zimbabwe 2016-2017. 2018; Available at: http://programme.aids2018.org/Abstract/Abstract/6374.

85. Nguyen K. Breastfeeding cessation, maternal adherence to antiretroviral therapy and HIV viremia in the early postpartum period: a prospective cohort study. 2016;

86. Watt MH, Cichowitz C, Kisigo G, et al. Predictors of postpartum HIV care engagement for women enrolled in prevention of mother-to-child transmission (PMTCT) programs in Tanzania. AIDS Care **2019**; 31:687–698.

87. Myer L, Phillips TK, Zerbe A, et al. Integration of postpartum healthcare services for HIV-infected women and their infants in South Africa: A randomised controlled trial. PLoS Med **2018**; 15:e1002547. Available at: https://www.ncbi.nlm.nih.gov/pmc/articles/PMC5877834/. Accessed 23 July 2020.

88. Harrington BJ, Pence BW, Maliwichi M, et al. Probable antenatal depression at antiretroviral initiation and postpartum viral suppression and engagement in care. AIDS **2018**; 32:2827–2833.

89. Luoga E, Vanobberghen F, Bircher R, et al. Brief report: No HIV transmission from virally suppressed mothers during breastfeeding in rural Tanzania. J Acquir Immune Defic Syndr **2018**; 79:e17–e20.

90. Gill MM, Hoffman HJ, Ndatimana D, et al. 24-month HIV-free survival among infants born to HIV-positive women enrolled in Option B+ program in Kigali, Rwanda: The Kabeho Study. Medicine (Baltimore) **2017**; 96:e9445.

91. Hosseinipour M, Nelson JAE, Trapence C, et al. Viral suppression and HIV drug resistance at 6 months among women in Malawi’s Option B+ program: Results from the PURE Malawi Study. J Acquir Immune Defic Syndr **2017**; 75 Suppl 2:S149–S155.

92. Onoya D, Sineke T, Brennan AT, Long L, Fox MP. Timing of pregnancy, postpartum risk of virologic failure and loss to follow-up among HIV-positive women. AIDS **2017**; 31:1593–1602.

93. Davis NL, Miller WC, Hudgens MG, et al. Maternal and breastmilk viral load: Impacts of adherence on peripartum HIV infections averted–The Breastfeeding, Antiretrovirals, and Nutrition Study. J Acquir Immune Defic Syndr **2016**; 73:572–580.

94. Stover J, Glaubius R, Kassanjee R, Dugdale C. Updates to the Spectrum/AIM model for the UNAIDS 2020 HIV estimates. J Int AIDS Soc **2021**; 24:e25778.

95. UNICEF. Countdown to 2030 - Countdown Country Dashboards. Available at: https://www.countdown2030.org/landing_page. Accessed 6 October 2022.

96. Ciaranello A, Lu Z, Ayaya S, et al. Incidence of World Health Organization stage 3 and 4 events, tuberculosis and mortality in untreated, HIV-infected children enrolling in care before 1 year of age: an IeDEA (International Epidemiologic Databases to Evaluate AIDS) East Africa regional analysis. Pediatr Infect Dis J **2014**; 33:623–9.

97. West NS, Schwartz SR, Yende N, et al. Infant feeding by South African mothers living with HIV: implications for future training of health care workers and the need for consistent counseling. Int Breastfeed J **2019**; 14:11. Available at: https://doi.org/10.1186/s13006-019-0205-1. Accessed 24 July 2020.

98. Patel MR, Mushavi A, Balachandra S, et al. HIV-exposed uninfected infant morbidity and mortality within a nationally representative prospective cohort of mother-infant pairs in Zimbabwe. AIDS **2020**; 34:1339–1346.

99. Komtenza B, Satyanarayana S, Takarinda KC, et al. Identifying high or low risk of mother to child transmission of HIV: How Harare City, Zimbabwe is doing? PLoS One **2019**; 14:e0212848.

100. Moyo F, Haeri Mazanderani A, Barron P, et al. Introduction of routine HIV birth testing in the South African National Consolidated Guidelines. Pediatr Infect Dis J **2018**; 37:559–563.

101. Kalk E, Kroon M, Boulle A, et al. Neonatal and infant diagnostic HIV-PCR uptake and associations during three sequential policy periods in Cape Town, South Africa: a longitudinal analysis. J Int AIDS Soc **2018**; 21:e25212.

102. Phelanyane F. Prevention of mother-to-child-transmission (PMTCT) of HIV in Khayelitsha, South Africa: A contemporary review of the service 20 years later. International AIDS Conference, 2020. Available at: https://cattendee.abstractsonline.com/meeting/9289/presentation/188. Accessed 28 July 2020.

103. Mnyani CN, Tait CL, Peters RPH, et al. Implementation of a PMTCT programme in a high HIV prevalence setting in Johannesburg, South Africa: 2002-2015. South Afr J HIV Med **2020**; 21:1024.

104. Spooner E, Govender K, Reddy T, et al. Point-of-care HIV testing best practice for early infant diagnosis: an implementation study. BMC Public Health **2019**; 19:731.

105. Bisschoff C, Coulon J, Isaacs Z, et al. HIV testing at birth: Are we getting it right? South Afr J HIV Med **2019**; 20:951.

106. Onoya D, Jinga N, Nattey C, Mongwenyana C, Mngadi S, Sherman G. Motivational interviewing retention counseling and child HIV testing in South Africa. In: CROI 2020: Abstract 786. Boston, MA: International Antiviral Society-USA, 2020. Available at: https://www.croiconference.org/wp-content/uploads/sites/2/resources/2020/ebook/croi2020-boston-abstract-ebook.pdf. Accessed 24 July 2020.

107. Massyn N, Pillay Y, Padarath A. District Health Barometer 2017/18. Durban, South Africa: Health Systems Trust, 2019. Available at: https://www.hst.org.za/publications/Pages/DHB20172018.aspx. Accessed 11 August 2020.

108. Mallampati D, Ford N, Hannaford A, Sugandhi N, Penazzato M. Performance of virological testing for early infant diagnosis: a systematic review. J Acquir Immune Defic Syndr **2017**; 75:308–314.

109. Mazanderani A, Moyo F, Sherman G. Missed diagnostic opportunities within South Africa’s early infant diagnosis program, 2010-2015. PLoS One **2017**; 12:e0177173.

110. Moyo F, Haeri Mazanderani A, Feucht UD, et al. Monitoring diagnosis, retention in care and viral load suppression in children testing HIV polymerase chain reaction-positive in two districts in South Africa. S Afr Med J **2019**; 109:686–692.

111. Smith ER, Sheahan AD, Heyderman RS, et al. Performance of HIV rapid tests among breastfeeding, Malawian infants. Pediatr Infect Dis J **2017**; 36:405–411.

112. Buchanan AM, Nadjm B, Amos B, et al. Utility of rapid antibody tests to exclude HIV-1 infection among infants and children aged <18 months in a low-resource setting. J Clin Virol **2012**; 55:244–9.

113. Desmond AC, Moodley D, Conolly CA, Castel SA, Coovadia HM. Evaluation of adherence measures of antiretroviral prophylaxis in HIV exposed infants in the first 6 weeks of life. BMC Pediatr **2015**; 15:23.

114. Kumwenda NI, Hoover DR, Mofenson LM, et al. Extended antiretroviral prophylaxis to reduce breast-milk HIV-1 transmission. N Engl J Med **2008**; 359:119–29.

115. McFarland EJ, Cunningham CK, Muresan P, et al. Safety, tolerability, and pharmacokinetics of a long-acting broadly neutralizing HIV-1 monoclonal antibody VRC01LS in HIV-1-exposed newborn infants. J Infect Dis **2021**; 224:1916–1924.

116. Chasela CS, Hudgens MG, Jamieson DJ, et al. Maternal or infant antiretroviral drugs to reduce HIV-1 transmission. N Engl J Med **2010**; 362:2271–81.

117. Coovadia HM, Brown ER, Fowler MG, et al. Efficacy and safety of an extended nevirapine regimen in infant children of breastfeeding mothers with HIV-1 infection for prevention of postnatal HIV-1 transmission (HPTN 046): a randomised, double-blind, placebo-controlled trial. Lancet **2012**; 379:221–8.

118. Flynn PM, Taha TE, Cababasay M, et al. Prevention of HIV-1 transmission through breastfeeding: Efficacy and safety of maternal antiretroviral therapy versus infant nevirapine prophylaxis for duration of breastfeeding in HIV-1-infected women with high CD4 cell count (IMPAACT PROMISE): A randomized, open-label, clinical trial. J Acquir Immune Defic Syndr **2018**; 77:383–392.

119. Marston M, Becquet R, Zaba B, et al. Net survival of perinatally and postnatally HIV-infected children: a pooled analysis of individual data from sub-Saharan Africa. Int J Epidemiol **2011**; 40:385–96.

120. Becquet R, Marston M, Dabis F, et al. Children who acquire HIV infection perinatally are at higher risk of early death than those acquiring infection through breastmilk: a meta-analysis. PLoS One **2012**; 7:e28510.

121. United Nations, Department of Economic and Social Affairs, Population Division. World Population Prospects 2019, Online Edition. Rev. 1. 2019. Available at: https://population.un.org/wpp/Download/Standard/CSV/. Accessed 27 July 2021.

122. World Health Organization. Global Health Estimates 2020: Deaths by Cause, Age, Sex, by Country and by Region, 2000-2019. 2020. Available at: https://www.who.int/data/maternal-newborn-child-adolescent-ageing/advisory-groups/gama/gama-advisory-group-members. Accessed 27 July 2021.

123. Yazdanpanah Y, Losina E, Anglaret X, et al. Clinical impact and cost-effectiveness of co-trimoxazole prophylaxis in patients with HIV/AIDS in Cote d’Ivoire: a trial-based analysis. AIDS **2005**; 19:1299--1308.

124. Amani-Bosse C, Dahourou DL, Malateste K, et al. Virological response and resistances over 12 months among HIV-infected children less than two years receiving first-line lopinavir/ritonavir-based antiretroviral therapy in Cote d’Ivoire and Burkina Faso: the MONOD ANRS 12206 cohort. J Int AIDS Soc **2017**; 20:1–13.

125. Mulenga V, Musiime V, Kekitiinwa A, et al. Abacavir, zidovudine, or stavudine as paediatric tablets for African HIV-infected children (CHAPAS-3): an open-label, parallel-group, randomised controlled trial. Lancet Infect Dis **2016**; 16:169–179.

126. Ferrand RA, Simms V, Dauya E, et al. The effect of community-based support for caregivers on the risk of virological failure in children and adolescents with HIV in Harare, Zimbabwe (ZENITH): an open-label, randomised controlled trial. Lancet Child Adolesc Health **2017**; 1:175–183.

127. Jiamsakul A, Kariminia A, Althoff KN, et al. HIV viral load suppression in adults and children receiving antiretroviral therapy–results from the IeDEA Collaboration. J Acquir Immune Defic Syndr **2017**; 76:319–329.

128. Kityo C, Boerma RS, Sigaloff KCE, et al. Pretreatment HIV drug resistance results in virological failure and accumulation of additional resistance mutations in Ugandan children. J Antimicrob Chemother **2017**; 72:2587–2595.

129. Teasdale CA, Sogaula N, Yuengling KA, et al. HIV viral suppression and longevity among a cohort of children initiating antiretroviral therapy in Eastern Cape, South Africa. J Int AIDS Soc **2018**; 21:e25168.

130. Moyo S, Ncube RT, Shewade HD, et al. Children and adolescents on anti-retroviral therapy in Bulawayo, Zimbabwe: How many are virally suppressed by month six? F1000Res **2020**; 9:191.

131. Boerma RS, Bunupuradah T, Dow D, et al. Multicentre analysis of second-line antiretroviral treatment in HIV-infected children: adolescents at high risk of failure. J Int AIDS Soc **2017**; 20:21930.

132. Venter WDF, Moorhouse M, Sokhela S, et al. Dolutegravir plus two different prodrugs of tenofovir to treat HIV. N Engl J Med **2019**; 381:803–815.

133. NAMSAL ANRS 12313 Study Group, Kouanfack C, Mpoudi-Etame M, et al. Dolutegravir-based or low-dose efavirenz-based regimen for the treatment of HIV-1. N Engl J Med **2019**; 381:816–826.

134. Stockdale AJ, Saunders MJ, Boyd MA, et al. Effectiveness of protease inhibitor/nucleos(t)ide reverse transcriptase inhibitor-based second-line antiretroviral therapy for the treatment of Human immunodeficiency virus type 1 infection in sub-Saharan Africa: a systematic review and meta-analysis. Clin Infect Dis **2018**; 66:1846–1857.

135. Venter WDF, Sokhela S, Simmons B, et al. Dolutegravir with emtricitabine and tenofovir alafenamide or tenofovir disoproxil fumarate versus efavirenz, emtricitabine, and tenofovir disoproxil fumarate for initial treatment of HIV-1 infection (ADVANCE): week 96 results from a randomised, phase 3, non-inferiority trial. Lancet HIV **2020**; 7:e666–e676.

136. Calmy A, Tovar Sanchez T, Kouanfack C, et al. Dolutegravir-based and low-dose efavirenz-based regimen for the initial treatment of HIV-1 infection (NAMSAL): week 96 results from a two-group, multicentre, randomised, open label, phase 3 non-inferiority trial in Cameroon. Lancet HIV **2020**; 7:e677–e687.

137. Paton NI, Kityo C, Hoppe A, et al. Assessment of second-line antiretroviral regimens for HIV therapy in Africa. N Engl J Med **2014**; 371:234–47.

138. Ford N, Orrell C, Shubber Z, Apollo T, Vojnov L. HIV viral resuppression following an elevated viral load: a systematic review and meta-analysis. J Int AIDS Soc **2019**; 22:e25415.

139. Pepperrell T, Venter WDF, McCann K, et al. Participants on dolutegravir re-suppress HIV RNA after virologic failure: updated data from the ADVANCE trial. Clin Infect Dis **2021**; 73:e1008–e1010.

140. Eholie SP, Moh R, Benalycherif A, et al. Implementation of an intensive adherence intervention in patients with second-line antiretroviral therapy failure in four west African countries with little access to genotypic resistance testing: a prospective cohort study. Lancet HIV **2019**; 6:e750–e759.

141. Violari A, Lindsey JC, Hughes MD, et al. Nevirapine versus ritonavir-boosted lopinavir for HIV-infected children. N Engl J Med **2012**; 366:2380–9.

142. Palumbo P, Lindsey JC, Hughes MD, et al. Antiretroviral treatment for children with peripartum nevirapine exposure. N Engl J Med **2010**; 363:1510–20.

143. Walmsley SL, Antela A, Clumeck N, et al. Dolutegravir plus abacavir-lamivudine for the treatment of HIV-1 infection. N Engl J Med **2013**; 369:1807–18.

144. Carlucci JG, Liu Y, Clouse K, Vermund SH. Attrition of HIV-positive children from HIV services in low and middle-income countries. AIDS **2019**; 33:2375–2386.

145. Makurumidze R, Mutasa-Apollo T, Decroo T, et al. Retention and predictors of attrition among patients who started antiretroviral therapy in Zimbabwe’s national antiretroviral therapy programme between 2012 and 2015. PLoS One **2020**; 15:e0222309. Available at: https://journals.plos.org/plosone/article?id=10.1371/journal.pone.0222309. Accessed 14 August 2020.

146. Fenner L, Brinkhof MWG, Keiser O, et al. Early mortality and loss to follow-up in HIV-infected children starting antiretroviral therapy in Southern Africa. J Acquir Immune Defic Syndr **2010**; 54:524–532.

147. Sengayi M, Dwane N, Marinda E, Sipambo N, Fairlie L, Moultrie H. Predictors of loss to follow-up among children in the first and second years of antiretroviral treatment in Johannesburg, South Africa. Glob Health Action **2013**; 6:19248.

148. Chhagan MK, Kauchali S, Broeck JV den. Clinical and contextual determinants of anthropometric failure at baseline and longitudinal improvements after starting antiretroviral treatment among South African children. Trop Med Int Health **2012**; 17:1092–1099. Available at: https://onlinelibrary.wiley.com/doi/abs/10.1111/j.1365-3156.2012.03026.x. Accessed 14 August 2020.

149. Janssen N, Ndirangu J, Newell M-L, Bland RM. Successful paediatric HIV treatment in rural primary care in Africa. Arch Dis Child **2010**; 95:414–421.

150. Zanoni BC, Phungula T, Zanoni HM, France H, Feeney ME. Risk factors associated with increased mortality among HIV infected children initiating antiretroviral therapy (ART) in South Africa. PLoS One **2011**; 6:e22706.

151. Meyer-Rath G, Brennan A, Long L, et al. Cost and outcomes of paediatric antiretroviral treatment in South Africa. AIDS **2013**; 27:243–250.

152. Chandiwana N, Sawry S, Chersich M, Kachingwe E, Makhathini B, Fairlie L. High loss to follow-up of children on antiretroviral treatment in a primary care HIV clinic in Johannesburg, South Africa. Medicine (Baltimore) **2018**; 97:e10901.

153. Kalawan V, Naidoo K, Archary M. Impact of routine birth early infant diagnosis on neonatal HIV treatment cascade in eThekwini district, South Africa. South Afr J HIV Med **2020**; 21:1084.

154. Bernard C, Balestre E, Coffie PA, et al. Aging with HIV: what effect on mortality and loss to follow-up in the course of antiretroviral therapy? The IeDEA West Africa Cohort Collaboration. HIV AIDS (Auckl) **2018**; 10:239–252.

155. Matare T, Shewade HD, Ncube RT, et al. Anti-retroviral therapy after ‘Treat All’ in Harare, Zimbabwe: What are the changes in uptake, time to initiation and retention? F1000Res **2020**; 9:287.

156. Makurumidze R, Buyze J, Decroo T, et al. Patient-mix, programmatic characteristics, retention and predictors of attrition among patients starting antiretroviral therapy (ART) before and after the implementation of HIV ‘Treat All’ in Zimbabwe. PLoS One **2020**; 15:e0240865.

157. Dorward J, Sookrajh Y, Gate K, et al. HIV treatment outcomes among people with initiation CD4 counts >500 cells/µL after implementation of Treat All in South African public clinics: a retrospective cohort study. J Int AIDS Soc **2020**; 23:e25479.

158. Hirasen K, Fox MP, Hendrickson CJ, Sineke T, Onoya D. HIV treatment outcomes among patients initiated on antiretroviral therapy pre and post-universal test and treat guidelines in South Africa. Ther Clin Risk Manag **2020**; 16:169–180.

159. Ambia J, Kabudula C, Risher K, et al. Outcomes of patients lost to follow-up after antiretroviral therapy initiation in rural north-eastern South Africa. Trop Med Int Health **2019**; 24:747–756.

160. National Health Laboratory Service. NHLS State Price List 2019/20. South African National Health Laboratory Service, 2019.

161. Bassett IV, Giddy J, Nkera J, et al. Routine voluntary HIV testing in Durban, South Africa: the experience from an outpatient department. J Acquir Immune Defic Syndr **2007**; 46:181–6.

162. The Global Fund. Price list. Available at: https://public.tableau.com/profile/the.global.fund#!/vizhome/PQRPricelist_English/PriceList. Accessed 27 October 2020.

163. The Global Fund. Pooled procurement mechanism reference pricing: ARVs (version: quarter 3 2020). The Global Fund, 2020. Available at: https://www.theglobalfund.org/media/5813/ppm_arvreferencepricing_table_en.pdf.

164. Clinton Health Access Initiative. Unitaid and CHAI announce agreement with Omega Diagnostics to increase access to new, instrument-free CD4 test for people living with HIV in over 130 low- and middle-income countries. 2020. Available at: https://www.clintonhealthaccess.org/unitaid-and-chai-announce-agreement-with-omega-diagnostics-to-increase-access-to-new-portable-cd4-testing-device-for-people-living-with-hiv-in-over-130-low-and-middle-income-countries/. Accessed 4 August 2020.

165. Tibuyile Sigudia (Central Medical Stores Swaziland). Personal communication. 2016;

166. Cleary S, Chitha W, Jikwana S, Okorafor OA, Boulle A. South African Health Review 2005: Financing. Durban, South Africa: Health Systems Trust, 2005. Available at: https://www.hst.org.za/publications/South%20African%20Health%20Reviews/sahr05.pdf.

167. World Health Organization. WHO-CHOICE: Country-specific unit costs. 2011. Available at: https://www.who.int/teams/health-systems-governance-and-financing/economic-analysis/costing-and-technical-efficiency/quantities-and-unit-prices-(cost-inputs)/econometric-estimation-of-who-choice-country-specific-costs-for-inpatient-and-outpatient-health-service-delivery. Accessed 18 August 2021.
